# Hypothesis-driven weight of evidence evaluation indicates ethylbenzene lacks endocrine disruption potential by EATS pathways

**DOI:** 10.17179/excli2024-7822

**Published:** 2025-03-27

**Authors:** Christopher J. Borgert

**Affiliations:** 1 Applied Pharmacology and Toxicology Inc, Gainesville FL, 32605 and University of Florida College of Veterinary Medicine, Dept. Physiological Sciences, Gainesville FL, 32610

**Keywords:** ethylbenzene, endocrine disruptor, data quality, mode of action, weight of evidence, estrogen agonist, estrogen antagonist, androgen agonist, androgen antagonist, thyroid inhibition, steroidogenesis

## Abstract

Ethylbenzene (EB) was placed on List 2 for Tier 1 endocrine screening in the U.S. EPA's two-tiered Endocrine Disruptor Screening Program (EDSP) and was scheduled for evaluation under TSCA. Results of toxicology studies on EB were used to evaluate estrogen, androgen, thyroid, and steroidogenic (EATS) endpoints by a Weight of Evidence (WoE) methodology, as required by U.S. EPA and OECD guidelines for evaluating a chemical's endocrine disruptive potential. The WoE method involved problem formulation, systematic literature search and selection, data quality evaluation, relevance weighting of endpoint data, and application of specific interpretive criteria. Data on EB were sufficient to assess its effects on endpoints that would be expected to respond to chemicals that operate via EATS modes of action (MoAs) in various screening assays (Tier 1) and toxicity tests (Tier 2) that evaluate reproduction, development, and sub-chronic and chronic toxicity. In those studies, EB produced a pattern of responses inconsistent with the responses that would be expected for hormones and chemicals known to operate via EATS MoAs. Endocrine-sensitive endpoints that respond to EB administration generally do so only at dose levels above its kinetic maximum dose, indicating a lack of relevance to potential effects at lower dose levels in either the test species or humans. This comprehensive WoE evaluation demonstrates that EB lacks the potential to exhibit endocrine disruptive properties and cannot be deemed an endocrine disruptor or potential endocrine disruptor. Because this WoE evaluation was based largely on Tier 2-level studies of the type considered by the U.S. EPA and OECD to be more definitive than results of Tier 1 EDSP screening results, no additional useful information would be obtained by subjecting EB to further endocrine screening. As such, further endocrine screening of EB would be unjustified from animal welfare perspectives. This analysis supports a regulatory decision to halt further testing of EB for endocrine disruption unless unique and compelling data to the contrary arise.

See also the graphical abstract(Fig. 1).

## Abbreviations

AMA: Amphibian Metamorphosis Assay

ARBA: Androgen Receptor Binding Assay

ATSDR: Agency for Toxic Substances and Disease Registry

CASRN: Chemical Abstracts Service Registry Number

CCL3: Third Contaminant Candidate List

CompTox: Computational Toxicology Dashboard

DART: Developmental and Reproductive Toxicology

EATS: Estrogen, Androgen, Thyroid, and Steroidogenesis

EDSP: Endocrine Disruptor Screening Program

EDSP21: Endocrine Disruptor Screening Program for the 21st Century Dashboard

ERBA: Estrogen Receptor Binding Assay

ERTA: Estrogen Receptor Transactivation Assay

EU: European Union

FSTRA: Fish Short-Term Reproduction Assay

HSDB: Hazardous Substances Data Bank

IC_25_: Inhibitory Concentration 25 %

IPCS: International Programme on Chemical Safety

KMD: Kinetically-derived maximum dose

LABC: Levator ani bulbocavernosus muscle

LOEC: Lowest observable effect concentration

NOEC: No observable effect concentration

MoA(s): Mode(s) of Action

NTP: National Toxicology Program

OECD: Organization for Economic Cooperation and Development

OSRI: Other Scientifically Relevant Information

RfD(s): Reference Dose(s)

TSCA: Toxic Substances Control Act

TSH: Thyroid Stimulating Hormone

T4: Thyroxin

U.S. EPA: United States Environmental Protection Agency

WEEL(s): Workplace Environmental Exposure Limit(s)

WHO: World Health Organization

WoE: Weight of Evidence

## Introduction

Ethylbenzene (EB) is on the second list of pesticides and industrial chemicals (“List 2”) prioritized for Tier 1 screening by the U.S. EPA [June 14, 2013, 78 FR 35922] as described previously (U.S. EPA, 2009[71]; Borgert, 2023[6]). Depending on the results of Tier 1 screening, Tier 2 testing may be required (U.S. EPA, 2011[73]). Tier 2 test results are used for human and ecological risk assessments (Borgert, 2023[6]). 

The Endocrine Disruptor Screening Program (EDSP) differs from regulatory programs in the European Union (e.g., European Parliament 2006[22], 2012[21]) in critical ways. The U.S. EPA regulates on risk rather than hazard; the EDSP does not attempt to establish a causal link between adverse effects identified in Tier 2 and endocrine modes of action (MoAs) identified in Tier 1; and the U.S. EPA does not attempt to place a special label on “endocrine disruptors,” which would require satisfying the WHO/IPCS definition (WHO/ IPCS, 2002[78]; WHO/UNEP, 2012[79]). In contrast, regulatory programs in the European Union seek to affix qualitative “hazard” labels to chemicals for purposes of classification and labeling, irrespective of whether a causal link can be established between an endocrine MoA and adverse effects, as required to satisfy the “endocrine disruptor” definition. Due to these differences, classification and labeling within the European Union may not align with the scientific data or with determinations of the U.S. EPA and other countries that regulate based on risk.

The U.S. EPA has modified the EDSP Tier 1 screening battery since 2009 when it was first applied to 52 chemicals on List 1 (List 1). The program, often referred to as “EDSP-21” (Browne et al., 2015[15]; Kleinstreuer et al., 2017[34]), may be combined with the results of exposure assessment models to further increase the speed and efficiency of endocrine assessments and prioritization of chemicals for endocrine screening (Rotroff et al., 2013[56]).

The Organisation for Economic Co-operation and Development (OECD) uses a slightly different approach. Tiers 2 through 5 of its 5-tiered Conceptual Framework (OECD, 2012[54]) includes the eleven EDSP screens and tests for potential endocrine MoAs as well as general toxicology and carcinogenicity studies that evaluate adverse effects on endpoints that could be affected by endocrine MoAs (OECD, 2012[54]). Weight of Evidence (WoE) procedures are required by the U.S. EPA (U.S. EPA, 2011[73]) and OECD (OECD, 2012[54]) programs to evaluate a chemical's potential for interaction with the endocrine system. Here, EB was evaluated by a widely used WoE approach (Borgert et al., 2011[12]), which was deemed appropriate for evaluating the available scientific data relevant to endocrine disruption (OECD, 2012[54]). It is important to appreciate that although this analysis bears several similarities to a systematic review, e.g., in searching and selecting literature according to previously defined criteria, it is properly designated a WoE analysis because it integrates data from diverse types of studies, weighted according to relevance and strength.

The U.S. EPA developed List 2, which includes 109 chemicals, through application of exposure criteria and a public comment process, as it did for List 1 (U.S. EPA 2009[72]; Borgert, 2023[6]). The TSCA reform bill (U.S. Public Law 114-182, June 22, 2016[74]) includes components of the EDSP in existing programs for industrial chemicals. The U.S. EPA recently released a prioritized plan for the EDSP to comply with requirements of the Federal Food, Drug and Cosmetics Act (U.S. EPA, 2023[70]) that will comprise a combination of Tier 1 screening assays, EDSP-21 bioactivity screening results, and more definitive studies as deemed necessary to make regulatory determinations of the potential for a chemical to interact with estrogen, androgen, thyroid, or steroidogenic pathways.

## Scope and Purpose

Irrespective of the regulatory program under which a chemical is evaluated, it is anticipated that there will be an opportunity for manufacturers and importers to submit “other scientifically-relevant information” (OSRI) as was the case for the test orders released for List 1 chemicals in 2009 and included in the Agency's near-term strategy (U.S. EPA, 2023[70]; Borgert, 2023[6]). This report constitutes an assessment of all scientifically-relevant data available regarding the potential for EB to act via EATS pathways and produce adverse effects via those MoAs. It was conducted consistent with the WoE methodology of Borgert et al. (2011[12], 2014[14]) as was used previously to evaluate styrene (Borgert, 2023[6]).

## Organization of the Evaluation

This evaluation first outlines the WoE methodology used to evaluate EB, including a description of the literature search and selection criteria, the literature evaluation, and the data compilation*. *Following the description of methods is a summary of “ToxRTool^”^ evaluations of the studies that met selection criteria, and a summary of EDSP-21 / ToxCast^TM^ results. Appendix A(Tab. 1) (References in Appendix A: Andrew et al., 1981[3]; Cragg et al., 1989[18]; Faber et al., 2006[23], 2007[24]; Li et al., 2010[40]; Mellert et al., 2007[44]; NTP 1992[52], 1999[53]; Saillenfait et al., 2006[59], 2007[60]; Stott et al., 2003[65]; Ungváry and Tátrai, 1985[69]) lists the details of the ToxRTool evaluations of each study, by number, e.g., [1], so that endpoint responses can be tracked by study number throughout the evaluation. The numbers are used in the next section to identify the studies from which data were extracted to evaluate the six EATS hypotheses, along with a concise explanation of the endpoint responses relevant to each hypothesis. The numbers also identify the studies corresponding to each endpoint response, which are listed by hypothesis in Supplementary Tables 1-6, and a summary of the results is provided in Supplementary Table 7. Additional information about this WoE analysis is found in Supplemental Materials A-C.

## Methods

### Literature search and selection

#### Literature search

The literature search strategy was conducted using the same search terms published previously (Borgert, 2023[6]), except that the CASRN (Ethylbenzene / 100-41-4) was used instead of chemical identifiers for styrene. 

#### Literature and data selection

The literature identified by the search strategy was initially triaged for separation into three categories according to whether the studies were Apparently Relevant, Possibly Relevant, or Apparently Not Relevant, as published previously (Borgert, 2023[6]). In June of 2024, a literature search update was conducted, and 56 additional studies were evaluated. A comprehensive list of literature evaluated is provided in Supplemental Material A. Only publications meeting the inclusion criteria published previously (Borgert, 2023[6]) were considered for the WoE evaluation. No studies were excluded because of low data quality however, data quality was considered in evaluating the overall WoE for each hypothesis.

The goals of data selection for this WoE evaluation were identical to those described previously for styrene (Borgert, 2023[6]). The criteria used were broad to ensure that the WoE evaluation included all of the data that might inform EATS MoA; consequently, these may be too broad to be useful for other purposes. For example, even though some routes of exposure are more relevant than others for risk assessment, route of exposure was not an exclusion or inclusion criterion for this WoE. Studies were not excluded based solely on the use of excessively high doses even though endocrine MoAs may be obscured by systemic toxicity at high doses (Marty et al., 2018[41]; Slikker et al., 2004[64]; Borgert et al., 2021[10]). Problems inherent to the use of high doses have been discussed previously (Borgert et al., 2021[10]) and are germane to WoE evaluations. This evaluation followed the logic and criteria applied previously (Borgert, 2023[6]).

The homeostatic role of the endocrine system and the fact that healthy physiological functioning is supported by wide ranges of normal endocrine organ weights and hormone levels creates a situation whereby values may be statistically significant compared to concurrent controls within a particular study, yet not indicative of adverse effects because they are within normal ranges based on historical control data for the test species. These factors and the broad inclusion criteria applied tend to bias this evaluation toward a false positive conclusion. Hence, this WoE methodology could reach only provisional conclusions that a chemical exhibits potential endocrine activity. On the other hand, this approach to data selection results in a high level of confidence in negative conclusions regarding the possibility that EB exhibits EATS activity. Few data were excluded by this literature search and data selection strategy, and the data gaps that exist did not prohibit a valid WoE determination.

### Consideration of the kinetically-derived maximum dose for EB

For purposes of this endocrine WoE evaluation, endpoint responses were not excluded or discounted based on dose-response. The conservatism of including endpoints regardless of the dose at which they respond is underscored by a consideration of the kinetically-derived maximum dose (KMD) for EB, as defined previously (Borgert et al., 2021[10]). The ability to identify a KMD for a chemical indicates that above a certain dose, a fundamental change occurs in the organism's ability to process a chemical, i.e., to absorb, distribute, detoxify, and eliminate it. Doses above the KMD often produce toxicity that is qualitatively different from toxicity produced by doses below it (Borgert et al., 2021[10]). In practice, the KMD is estimated based on a range that represents our uncertainty about the precise location of the KMD (Burgoon et al., 2022[17]). Using published kinetic data on EB, a KMD was recently identified (Burgoon et al., 2023[16]). The KMD range in rats was estimated to be 8-17 mg/L venous EB, and in humans, from 10-18 mg/L venous EB. These blood concentration ranges correspond to an inhalation concentration of approximately 200 ppm EB (Burgoon et al., 2023[16]). 

It is important to appreciate that endpoints responding only at exposure levels above 200 ppm EB are unreliable for MoA analysis and cannot be used to infer any specific MoA for EB, including an endocrine MoA. For endpoint responses observable only above the KMD, the MoA involves, and may solely depend upon, high-dose dependent changes in the kinetics of EB that do not confound endpoint responses that occur at doses well below the KMD. Although all endpoint responses were tabulated and used to evaluate the overall pattern of responses for each endocrine hypothesis, the paucity of endpoints that respond below the KMD for EB or 200 ppm underscores that the conclusions reached are highly conservative.

Of the dozens of endpoints evaluated among thirteen studies included in this WoE, only four endpoints, two in each of two studies, showed responses to EB exposure below the KMD. A fifth endpoint from a third study might be considered, but is dubious. Those included:

1. The percentage of dead or resorbed fetuses was increased in all EB-exposed groups in rats (138, 276 and 553 ppm); however, there was no significant difference in the percentage of dead or resorbed fetuses in EB-exposed mice or rabbits compared with controls (Ungváry and Tátrai, 1985[69]).
2. The mean age of acquisition of vaginal patency was reduced in all exposed groups (25, 100 and 500 ppm EB) compared to the concurrent control group in F_1_ female rat offspring; similar differences were not observed in the F_2_ female pups. All mean values were comparable to the historical control mean value, and therefore, the authors felt these differences were not biologically important (Faber et al., 2006[23]).
3. The percentage of weight-retarded male and female fetuses was significantly greater in rats exposed to EB at a concentration of 553 ppm and in female rabbit fetuses at 115 ppm compared with controls, but there was no significant difference in mean fetal weights in mice exposed to EB 3-4 hours/day intermittently at 115 ppm (Ungváry and Tátrai, 1985[69]).
4. Absolute and relative thyroid weights were increased (approximately 18-20 % and statistically significant) in the F_0_ males exposed to 100 and 500 ppm, but these increases were not observed in the F_1_ male group or in females exposed to the same EB concentrations (Faber et al., 2006[23]).
5. Statistically non-significant trends were observed in the incidences of thyroid follicular cell hyperplasia in mice in both males (control: 21:50; 75 ppm: 21:50; 250 ppm: 29:50) and females (18:50, 23:50, 25:50). Relative to chamber controls, statistically significant increased incidences were observed only in 750 ppm males (32:50) and females (35:50). There were no significant differences between control and exposed rat thyroids upon histopathological examination (NTP, 1999[53]).

### Literature evaluation & data compilation

#### Data quality assessment

Data used for testing the MoA hypotheses were subjected to a quality evaluation according to their primary, secondary, and tertiary validity as described previously (Borgert, 2023[6]; Supplemental Material in Borgert et al., 2011[12]) consistent with international (e.g., OECD; U.S. EPA) toxicological guidelines, as described (Borgert et al., 2016[8]). Klimisch et al. (1997[35]) criteria were applied using the ToxRTool scoring system created by the European Center for the Validation of Alternative Methods (Schneider et al. 2009[62]) and causal relationships were evaluated consistent with the U.S. EPA's guidance on data quality assessment (U.S. EPA 2011[73]) and concepts published previously (Borgert, 2023[6]; Borgert et al., 2011[12]). Supplemental Material B contains a brief description of each endpoint used to evaluate each hypothesis, similar to that published previously for styrene (Borgert, 2023[6]).

The literature search strategy used here attempted to identify all data that might be informative regarding an endocrine mechanism of action underlying outcomes of EB exposure. Because of its broad and exhaustive nature, the literature search identified several studies that were speculative or severely limited, and could not be ranked for relevance consistent with the method (Borgert et al., 2011[12], 2014[14]; Borgert, 2023[6]). Eight such studies (Gong et al., 2018[26], 2023[25]; Harrath et al., 2022[33]; Lei et al., 2023[39]; Nakhjirgan et al., 2019[49]; Rouget et al., 2021[57]; Werder et al., 2019[76], 2020[75]) were not used in this evaluation, but are referenced as per the rationale described previously (Borgert, 2023[6]), as explained briefly in Supplemental Materials C.

### Weight-of-evidence methodology

The methodology used for this WoE analysis was designed to be broadly applicable within any hypothesis-testing paradigm that can be tested based on objective data (Borgert et al., 2011[12]). This hypothesis-driven framework was initially applied to endpoints measured by the U.S. EPA's EDSP Tier 1 screening assays and involved hypotheses related to interactions with specific endocrine MoAs (i.e., EATS pathways). The methodology requires weighting the importance of the data with respect to their mechanistic relevance for each hypothesized MoA. For these endocrine WoE evaluations, endpoints were categorized according to three ranks, and endpoint responses to the chemical were then interpreted by an algorithm that sequentially considered them in order of their importance to the hypothesis. The rankings have been described previously (Borgert et al., 2014[14]), and have been adapted to accommodate endpoints assessed in long-term toxicity tests, consistent with previously published data and approaches (Afarinesh et al., 2020[1]; Andrews et al., 2002[4]; Biegel et al., 1998[5]; Delclos et al., 2009[19]; Borgert, 2023[6]; Mihaich and Borgert, 2018[46]; Mihaich et al., 2017[45]; Neal et al., 2017[50]; NTP, 2010[51]). Because endocrine screening assays do not determine whether a chemical produces adverse effects by the endocrine mechanism probed, they cannot determine whether a chemical possesses endocrine disruptive properties. 

The relevance rankings and their use in evaluating endpoints was applied here as described previously (Borgert, 2023[6]). The rationale and complexities are fully described therein and are not repeated here. Many endpoints evaluated by this WoE method are relevant to more than one hypothesis, consistent with prior literature on endocrine screening methods (EDSTAC, 1998[20]; U.S. EPA, 2011[73]; Borgert et al., 2011[12][13]). It is important to recognize that the relevance rankings are specific to each hypothesis, and that the relevance ranking for an endpoint may differ across hypotheses. Because EB has been subjected to extensive toxicity testing, but not to the EDSP Tier 1 screening battery, there are several data gaps for Rank 1 endpoints measured following EB exposure. However, due to the large amount of data from more definitive toxicity studies, and the fact that a lack of response in endocrine-sensitive endpoints is more informative than a response (Borgert et al., 2011[12], 2014[14]; Borgert, 2023[6]), the data gap does not diminish this WoE evaluation. 

Like styrene (Borgert, 2023[6]), the magnitude of the responses produced by EB was not informative regarding EB's potency via an endocrine pathway because the data available for this WoE evaluation comprise mostly apical endpoints that can be affected by various MoAs other than EATS. Statistically significant non-monotonicity was not discounted, but mechanistic interpretations would be extremely tenuous if based on apparent “trends” that occur within the normal biological range for hormone-sensitive endpoints, since those endpoints can fluctuate for numerous reasons. A more complete discussion of these issues was published previously (Borgert, 2023[6]).

## Results – Data Selection, Data Quality Evaluation, and High-Throughput Screening

Thirteen studies provided data useful for determining whether EB can operate through EATS MoAs (Appendix A(Tab. 1)). In Supplementary Tables 1-6, “*Assay” *(second column from the left) means the general type of toxicological study in which the endpoint was measured. Similar endpoints are measured in different types of studies, but the conditions of those studies are often different. Therefore, the same endpoint from different types of studies may be included within the same MoA table and in different MoA tables depending on its relevance for evaluating the various endocrine MoA(s) that can affect it. This is important for understanding the discussion of Supplementary Tables 1-6. Of the thirteen studies, only Ungváry and Tátrai (1985[69]) [11] differed significantly from guideline regulatory toxicology studies. Details are provided in Appendix A(Tab. 1).

### ToxRTool summary

The Toxicological data Reliability Tool known as ToxRTool (Schneider et al., 2009[62]) was applied to the thirteen studies used here. Twelve of the thirteen studies used in this evaluation [1, 2, 3, 4, 5, 6, 7, 8, 9, 10, 12, 13] met all ToxRTool reliability criteria (21 for *in vivo* and 18 for *in vitro* studies). One publication (Mellert et al., 2007[44]; [3]) lacks a description of histological findings, but those findings were made available via the original study report, and thus, this study merits a score of 21 for the WoE evaluation. ToxRTool evaluation summaries are found in Appendix A(Tab. 1), listed by study number as described above. An explanation of deficiencies for studies that did not meet all criteria is included.

No studies were eliminated from consideration or discounted based on ToxRTool results; however, ToxRTool informed the overall quality of the data selected for the WoE and the interpretation of conflicting results between studies. Because few studies reported a response to EB and ToxRTool scores were similar regardless of whether an effect was reported, interpretations were unaffected.

### Results for Ethylbenzene in U.S. EPA's ToxCast^TM^ high throughput and EDSP 21 assays 

Results available for the U.S. EPA's CompTox Chemicals Dashboard and the National Toxicology Program's [NTP] Integrated Chemical Environment [ICE]) show that EB produced no reliable activity in the Tox21 suite of high-throughput *in vitro* and *in silico* assays. Those included assays for potential agonism or antagonism via estrogen receptors, androgen receptors, thyroid hormone receptors, thyroid stimulating hormone receptors, and assays for aromatase inhibition. However, these results are unreliable due to the lack of EB detectable in the wells according to the Tox21 Program (QC Grade: FNS - no sample detected (biological activity unreliable), per ICE. 

Although the U.S. EPA's CompTox Chemicals Dashboard returns a single active hit call for the RXR receptor (not an endocrine endpoint) via the assay TOX21 RXR BLA Agonist_ratio, the Tox21 Program noted that no EB results are trustworthy due to quality concerns. Data quality flags were not available through the U.S. EPA's Chemicals Dashboard, but are provided in NTP's ICE knowledgebase. More information on the chemical QC can be found at https://ice.ntp.niehs.nih.gov/DATASETDESCRIPTION?section=cHTS and in this download file from ICE (https://ice.ntp.niehs.nih.gov/downloads/MOA/ChemicalQC.xlsx). 

Both U.S. EPA's Chemicals Dashboard and ICE were accessed February 22, 2024.

**Figure 2:**
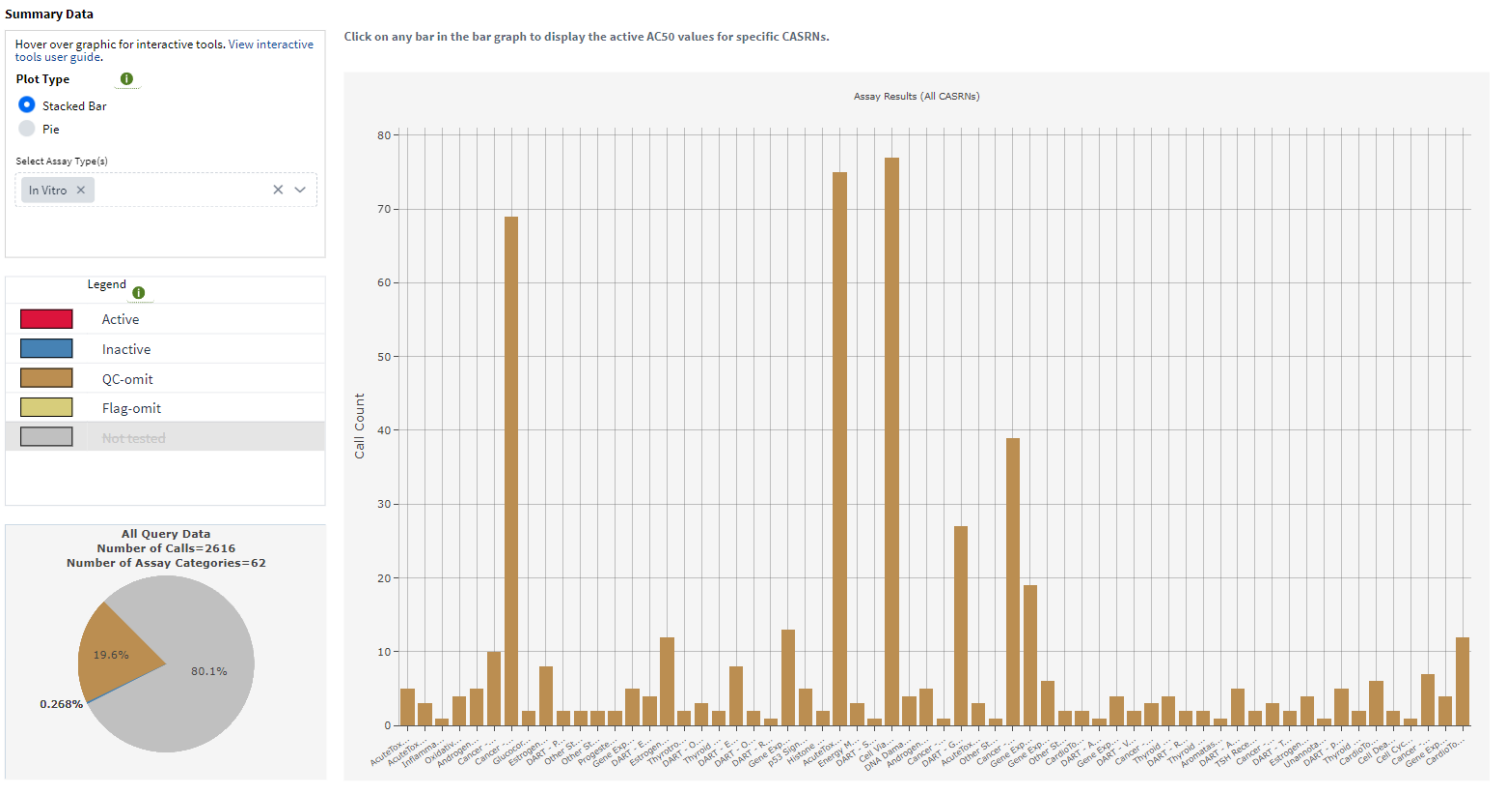
ToxCast / Tox21 data summary from the Integrated Chemical Environment (ICE) Database showing interpretable results (Active / Inactive) and uninterpretable results (QC Omit and Flag Omit) for ethylbenzene

The following charts were downloaded from the Integrated Chemical Environment (ICE) showing data quality flags for ToxCast^TM^ / Tox12 results (Figure 2(Fig. 2)).

## Results – Endocrine WoE Evaluation for EB

### Evaluation of estrogen agonist MoA

Rank 1 endpoints for the estrogen agonist MoA were not measured following exposure to EB (Supplementary Table 1). For this analysis, responses to EB were available for 20 out of 53 potential Rank 2 endpoints that are relevant for evaluating the estrogen agonist MoA among five repeat dose toxicity studies [1, 3, 9, 10], five developmental toxicity studies [4, 5, 6, 11, 13], and one reproductive toxicity study [2]. One study [2] included both reproductive and developmental endpoints, and one study [13] included both repeat dose and developmental endpoints; endpoints were counted under a single category to avoid over-counting. The analysis indicated that none of the 20 endpoints exhibited consistent responses to ethylbenzene exposure, with 16 endpoints remaining unchanged across all studies in which they were measured. Rank 2 endpoints that failed to respond in any study in which they were measured included testes atrophy and weight and histopathology of epididymides, ovaries, uterus, and vagina in repeat dose toxicity studies; number of corpora lutea and pre-implantation loss in developmental toxicity studies; and fertility, gestational length, number of implantations, litter size, mating index, ovarian follicle count in offspring, epididymal sperm counts, and time to mating in a reproductive toxicity study. Gross pathology, a Rank 3 endpoint, was unaffected in one repeat dose toxicity study.

Only four endpoints responded to EB in the direction expected for the estrogen agonist MoA, but none were consistently changed across studies (Supplementary Table 1). Epididymis weight was decreased in mice but not in rats in one repeat dose toxicity study [9]. Moreover, this occurred only at the highest exposure concentration tested (1000 ppm) and was not considered biologically significant by the study authors since spermatid counts, sperm motility, and caudal weight were normal. In this study, other Rank 2 endpoints for the estrogen agonist MoA were unchanged in either species, including testes weight and histopathology of the epididymis, uterus, and ovaries. Hence, in this study, EB did not produce a pattern of responses consistent with activity via the estrogen agonist MoA.

Supplementary Table 1 also shows that in developmental toxicity studies, post-implantation loss was unchanged in rats in three studies [4, 5, 6], was increased in rabbits but not rats in one study [13], and in rats but not in rabbits or mice in another [11]. The lack of consistency of the response of this endpoint and its occurrence at high doses suggests that, where observed, post-implantation losses were due to general toxicity rather than to changes in endocrine function. In the reproduction study [2], time to vaginal patency was decreased in the F_1_ generation of female pups for all exposed groups (25, 100 and 500 ppm EB) relative to the concurrent controls, however, the differences are unlikely to be biologically significant because the mean values were comparable to the historical control mean value [2]. No change in time to vaginal patency was observed in the F_2_ offspring [2]. That study [2] found no change in ovarian follicle counts or gestational length. Thus, the slight but statistically significant decrease in time to vaginal patency in one reproduction study [2] provides no evidence of activity via the estrogen agonist MoA. In the reproduction arm of the same study, estrous cyclicity was slightly reduced at the highest exposure concentration (500 ppm) in the parental (F_0_), but not in the F_1_ generation, however the difference is unlikely to be biologically significant because all females in the exposure group cycled normally and the cycle length was within normal values for the strain of rat used in the study [2]. This also fails to provide evidence of activity via the estrogen agonist MoA.

In summary, even if the few positive responses in Rank 2 endpoints were to be considered biologically significant, the endpoints that responded to EB are inconsistent with the response pattern expected of a chemical that acts via an estrogen agonist MoA. Supplementary Table 1 shows that most Rank 2 endpoints measured for the estrogen agonist MoA were consistently unaffected by EB, including histopathology of the vagina, uterus, ovaries, epididymides, as well as ovary weights in repeat dose toxicity studies. In reproductive toxicity studies, endpoints unresponsive to EB include gestational length, ovary weight, estrous cyclicity, time to mating, gross pathology and histopathology of vagina and prostate, and time to vaginal patency and ovarian follicle count in offspring. 

Although thirty-three of a possible fifty-three endpoints relevant to the estrogen agonist MoA were not measured in the available studies, the high degree of consistency among those endpoints that failed to respond to EB is sufficient evidence to conclude that EB lacks the potential to act via this endocrine MoA. Not only is the pattern of endpoint responses elicited by EB inconsistent with activity via the estrogen agonist MoA, but several additional observations strengthen the conclusion. A very low proportion of endpoints responded to EB in any study and the magnitude of response was small when observed. Although statistically significant, some endpoint responses were unlikely to be biologically significant because they were within the normal range for the rodent test strain. Together with the lack of consistency of any of those responses across studies, these observations confer very high confidence in the conclusion that EB lacks the potential to act as an estrogen agonist.

### Evaluation of estrogen antagonist MoA

Responses to EB were not measured in any Rank 1 endpoints for the estrogen antagonist MoA (Supplementary Table 2). Thirteen of a possible twenty-six endpoints of Rank 2 relevance for the estrogen antagonist MoA were evaluated among four repeat dose toxicity studies [1, 3, 9, 10], two developmental toxicity studies [5, 13] and one reproductive toxicity study [2] conducted with EB. Among those studies, most Rank 2 endpoints measured for the estrogen antagonist MoA were unaffected by EB. In two developmental toxicity studies, the number of corpora lutea were unchanged. Additionally, no relevant endpoints, including testes weight and histopathology of testes, epididymis, prostate, seminal vesicle, and ovaries were altered in the four repeat dose toxicity studies.

Supplementary Table 2 also shows that in a reproductive toxicity study, time to mating, litter size, epididymal sperm count, and fertility were unaffected, but two endpoints were altered in one of the two generations evaluated. Time to vaginal patency was increased in the F_1_, but not in the F_2_ generation. Estrous cyclicity was slightly reduced at the highest exposure concentration (500 ppm) in the parental, but not in the F_1_ generation, however the difference is unlikely to be biologically significant because all females in the exposure group cycled normally and the cycle length was within normal values for the strain of rat used in the study [2]. 

In summary, the available data provide sufficient evidence to conclude that EB lacks the potential to act as an antagonist in the estrogen pathway because the pattern of endpoints that responded to administration of EB is inconsistent with the pattern of responses expected of a chemical with estrogen antagonist MoA. That conclusion is strengthened by the lack of replication of responses across studies, confounding due to other MoAs operative at the excessively high doses required to elicit responses when they were observed, and the fact that many of the endpoints responded only at doses exceeding the KMD for EB. Finally, although statistical significance of the responses was established in comparison to concurrent controls, the biological significance was not typically established by a formal comparison to historical control values. Thus, there is very high confidence in the conclusion that EB does not act as an estrogen antagonist. 

### Evaluation of androgen agonist MoA

Responses to EB were not measured in any Rank 1 endpoints for the androgen agonist MoA (Supplementary Table 3). Eighteen of a possible forty-seven Rank 2 endpoints relevant for androgen agonism were measured among four repeat dose toxicity studies [1, 3, 9, 10], four developmental toxicity studies [4, 5, 6, 13], and one reproductive toxicity study [2] conducted with EB. No endpoints were altered by EB in repeat dose toxicity studies in which sperm count, testes weight, and histopathology of testes and ovary were evaluated. EB slightly reduced litter size in rabbits at the highest dose administered in one developmental toxicity study [13] but had no effect on rats or on any other endpoint in rabbits in that study. EB also did not alter any endpoints relevant to an androgen agonist MoA in four other developmental toxicity studies in which litter size, sex ratio, and number of implantations were measured. 

Supplementary Table 3 also shows that in a reproductive toxicity study [2], no change was observed in prostate weights of male offspring, sperm counts, sex ratio, time to mating, fertility, mating index, litter size, or number of implantations. Three endpoints were different from controls, but the authors of the study considered the changes too small to be biologically meaningful. Time to vaginal patency was increased in F_1_ females in all exposure groups compared with the control group, however, mean values were comparable to historical controls and thus, the change was not considered to be biologically important. No change in time to vaginal patency was observed in the F_2_ females. Estrous cyclicity for the F_0_ was reduced compared to the F_0_ control group in EB-exposed animals, however, this change was biologically insignificant because all females in this group were cycling normally, and their mean estrous cycle length (4.0 ± 0.3 days versus 4.4 ± 0.8 days) was within the 4-5 day range for estrous cycles normally exhibited by this strain of rat. Mean estrous cycle length did not differ between control and experimental F_1_ offspring. Time to balano-preputial separation (PND 44.7 + 2.0) was unaffected in F_2_ treatment groups. It was reduced in the F_1_ offspring only at the highest exposure concentration (500 ppm) compared to concurrent F_1_ controls (PND 43.5 + 2.2), however, this mean value was close to the F_2_ controls (PND 45.3) and to historical control values from the conducting laboratory (PND 44.7), and, therefore, was not considered by the authors to be biologically meaningful.

No endpoint relevant for evaluating the androgen agonist MoA consistently responded to EB in any study or across studies, and most endpoints failed to respond in any study in which they were measured. Although several endpoints were not measured that would have provided relevant information, there is sufficient evidence available to conclude that EB produces a pattern of endpoint responses inconsistent with activity via the androgen agonist MoA. Thus, there is high confidence that EB does not act as an androgen agonist.

### Evaluation of androgen antagonist MoA

Responses to EB were not measured in any Rank 1 endpoints for the androgen antagonist MoA (Supplementary Table 4). Seventeen of forty-five Rank 2 endpoints for the androgen antagonist MoA were measured among four repeat dose toxicity studies [1, 3, 9, 10] and one reproductive toxicity study [2] conducted with EB. Histopathology of the epididymis, ovary, prostate, seminal vesicles, testes, and uterus was unaffected by EB exposure in repeat dose toxicity studies. In the single repeat dose toxicity study in which it was measured [9], epididymis weight was reduced in mice, but not in rats. The decrease occurred only at the highest dose and was not considered to be biologically significant since epididymis histopathology, spermatid counts, sperm motility, and caudal weight were unchanged. 

Supplementary Table 4 also shows that EB did not affect sperm count, sperm motility, prostate weight, gross pathology, time to mating, fertility, and litter size in a reproductive toxicity study [2]. EB had no effect on time to balano-preputial separation (PND 44.7 + 2.0) in F_2_ treatment groups. EB reduced the time to balano-preputial separation in F_1_ offspring, but only at the highest exposure concentration (500 ppm) compared to concurrent F_1_ controls (PND 43.5 + 2.2). The reduced mean value, however, was close to the F_2_ controls (PND 45.3) and to historical control values from the conducting laboratory (PND 44.7), and, therefore, was not considered by the authors to be biologically meaningful. Estrous cyclicity for the F_0_ was reduced compared to the F_0_ control group in EB-exposed animals. However, this change was biologically insignificant because all females in this group were cycling normally and their mean estrous cycle length (4.0 ± 0.3 days versus 4.4 ± 0.8 days) was within the 4-5-day range for estrous cycles normally exhibited by this strain of rat. Mean estrous cycle length did not differ between control and experimental F_1_ offspring. 

No endpoint relevant for evaluating the androgen antagonist MoA consistently responded to EB in any study or across studies, and most endpoints failed to respond in any study in which they were measured. Although twenty-eight Rank 2 endpoints (of 45 total) were not measured (Supplementary Table 4), the consistency of negative responses among the seventeen endpoints that were measured shows that the pattern of endpoint responses elicited by EB is inconsistent with activity via the androgen antagonist MoA. Thus, there is high confidence that EB does not act via the androgen antagonist MoA. 

### Evaluation of thyroid inhibition MoA

EB was not evaluated in Rank 1 endpoints for the thyroid inhibition MoA, as shown in Supplementary Table 5. Effects of EB were measured in six of a possible twenty-one Rank 2 endpoints for the thyroid inhibition MoA among four repeat dose toxicity studies [1, 3, 9, 10], five developmental toxicity studies [4, 5, 6, 11, 13], and a reproductive toxicity study [2]. EB produced mixed results among Rank 2 endpoints in repeat dose and developmental toxicity studies, but the positive responses are likely to be caused by systemic toxicity rather than via a thyroid MoA. Six of a possible twenty-one Rank 3 endpoints were measured among six repeat dose toxicity studies [3, 6, 7, 8, 9, 10], the reproductive toxicity [2] and a developmental neurotoxicity [12] study. This number of Rank 3 results provide useful corroboration of Rank 2 results.

Among the Rank 2 endpoints measured, only thyroid follicular cell histopathology and thyroid weight reflect responses within the thyroid gland itself (Supplementary Table 5). Thyroid follicular cell histopathology was unchanged in rats in four [1, 3, 9, 10] repeat dose toxicity studies, unchanged in rabbits in one [10] study, and unchanged in mice in two [9, 10] studies. In one repeat dose toxicity study [1], a positive trend in follicular cell hyperplasia was identified as statistically significant with chronic exposure of mice but not rats, but only at the highest exposure concentration of 750 ppm. Liver weight, a Rank 3 endpoint for potential thyroid activity, was not measured in this chronic toxicity and carcinogenicity study [1], however, data summary tables and text from the report [1] clearly state that in addition to thyroid gland follicular cell hyperplasia, EB exposure increased the incidence of syncytial alteration of hepatocytes, hepatocellular hypertrophy, and hepatocyte necrosis, and hyperplasia of renal tubules and of the pituitary *pars distalis*, at the highest dose administered to mice. Therefore, it is likely that the thyroid follicular cell hyperplasia observed in mice in this study was caused by a general non-specific toxicity that leads to hyperplastic changes in many organs and tissues, rather than through a MoA involving the thyroid hormone pathway or feedback system. It is well established that many non-endocrine MoAs can produce changes in endocrine endpoints (Marty et al., 2018[41]). Therefore, it is highly unlikely that this endpoint response reflects a potential for thyroidal activity of EB. 

Absolute and relative thyroid weights were 18-20 % higher than concurrent controls in F_0_ males exposed to 100 and 500 ppm EB in a reproductive toxicity study [2], but these increases were not replicated in the F_1_ male group or among female animals [Supplementary Table 5, Rank 2]. Because this increase occurred only in F_1_ males, the authors attributed it to normal biological variation and not to EB exposure. Histopathological examination of the thyroid tissue was conducted but pathology was not reported, implying that pathologic changes were not observed. Therefore, it is unlikely that the increased thyroid weights were produced by a thyroid MoA. Pup growth and survival, also Rank 2 endpoints for thyroid inhibition, were not affected by EB in this study, further supporting the interpretation that EB exerted no thyroid activity in this study. 

Liver weight increase, a Rank 3 endpoint for the thyroid inhibition MoA when measured in repeat dose and reproductive toxicity studies (Supplementary Table 5), was observed with sub-chronic or short-term administration in mice and rats [9, 10], rats [3, 6, 7] and mice [8], but not in rabbits [10]. Three of these studies also evaluated histopathology of the thyroid gland, but observed no changes [3, 9, 10]. The liver weight increases observed in these studies occurred at the highest doses tested, were reversible in all three studies and were considered adaptive on two studies [6, 10]. In mice, the liver weight changes occurred secondary to regio-specific liver enzyme induction [8]. Thus, there is no evidence suggesting that EB increases liver weights via a thyroidal or thyroid hormone-related MoA. Other Rank 3 endpoints were unchanged by EB exposure in a developmental toxicity study [12], including auditory startle response, motor activity, learning and memory, and brain morphometry. 

Fetal weight and fetal survival (Rank 2) were reduced in rats and rabbits in some developmental toxicity studies with EB, but not in others. Fetal weights were decreased in rats at exposure levels of 1,000 ppm and greater secondary to reduced maternal weight gain and/or food consumption in some studies [4, 5, 6] but not in rats or rabbits in another study [13]. In rats and rabbits but not in mice, exposure to 552 ppm EB decreased fetal weights [11], however, 552 ppm and lower concentrations also resulted in aborted fetuses. Therefore, this effect is likely to have been the result of systemic toxicity rather than to a thyroid MoA. Fetal survival was slightly reduced in rabbits [11, 13] at high doses that may have produce maternal toxicity, but not in rats [4, 5, 6, 11, 13]. Thus, this consistency of negative responses among Rank 3 endpoints corroborates that EB lacks the potential for activity via the thyroid MoA.

In summary, although Supplementary Table 5 shows that some data gaps exist, the data available from repeat dose, developmental, and reproductive toxicity studies are sufficient to allow evaluation of EB's potential to act via a thyroid MoA. Where changes were elicited by EB in endpoints relevant to these hypotheses, these were observed inconsistently across studies, were within the normal ranges for the test species, and occurred at high doses. Effects on thyroid-relevant endpoints occurred secondary to high-dose, generalized liver toxicity. Therefore, the available data indicate that EB lacks the potential to cause effects via thyroid inhibition and there is high confidence in this interpretation.

### Evaluation of steroidogenic enzymes MoA

Of a possible 37 endpoints relevant for assessing the potential to act via a steroidogenic MoA, eleven were measured following exposure to EB (Supplementary Table 6). Of those ten endpoints, nine are Rank 2 endpoints and one is Rank 3. Only estrous cyclicity (Rank 2) responded to EB in a reproductive toxicity study in rats [2]. Although estrous cyclicity was slightly reduced at the highest exposure concentration (500 ppm) in the parental, but not in the F_1 _generation, the difference is unlikely to be biologically significant because all females in the exposure group cycled normally and the cycle length was within normal values for the strain of rat used in the study [2]. All other Rank 2 and 3 endpoints relevant for assessing activity via the steroidogenic MoA for which data were available were unaffected by EB in repeat dose toxicity studies, including histopathology of the ovaries, uteri and testes [1, 3, 9, 10] and gross pathology [3]. Sex ratio was unchanged in a developmental toxicity study [5] and sperm count, fertility, mating index, sex ratio, and number of live births were unchanged in a reproductive toxicity study [2]. Although several data gaps exist for the steroidogenic pathway (Supplementary Table 6), there was a consistent lack of response among the eleven endpoints that were measured. This is sufficient to provide a clear indication that EB lacks the potential to cause effects by a steroidogenic MoA.

In summary, the available data provide sufficient and strong evidence that EB lacks the potential to disrupt steroidogenesis because the pattern of endpoint responses elicited by EB is inconsistent with this MoA. Although data is lacking for several endpoints that are relevant for evaluating the steroidogenesis MoA, there is high confidence in that conclusion due to the consistency of negative responses across studies for the endpoints measured. Therefore, it is unlikely that conducting evaluations of these missing endpoints would reveal positive findings. Even if a few positive findings were identified, it is unlikely this would shift the weight-of-evidence to support a potential for endocrine-mediated adverse effects.

## Discussion

This Weight of Evidence (WoE) evaluation was carried out using an established methodology (Borgert et al., 2011[12][13]) that incorporates essential components for an unbiased, transparent, and thorough analysis of the potential for EB to act through EATS MoAs. Key elements of this evaluation include precise problem formulation, a systematic literature search and selection process based on defined criteria, assessment of data quality using published methodologies, consistent criteria for weighting data relevance, and interpretation of findings in alignment with expected response patterns produced by chemicals and hormones that operate via these MoAs. The methodology employed in data selection yielded a dataset adequate for a comprehensive WoE evaluation. While acknowledging certain data gaps, there was sufficient information available to evaluate the impact of EB on most endpoints linked to EATS MoAs in the domains of reproductive toxicity, developmental toxicity, and repeat dose toxicity studies.

Several conceptual and methodological limitations apply to WoE evaluations. A critical appraisal of WoE methods described several deficiencies that are important to avoid when developing a WoE framework (Krimsky, 2005[36]). Krimsky asserts that many WoE methodologies fall short of their intended objectives, which include improving the clarity and transparency of evaluations, enhancing the consistency of regulatory decisions, and elucidating the foundational assumptions that underpin these methodologies. He highlights that the epistemic basis of WoE approaches is often left ambiguous, leading to diminished clarity and consistency, and potentially compromising scientific integrity and validity. According to Krimsky (2005[36]), the *a priori* assumptions regarding the value of various evidentiary modalities tend to rely on expert judgments rather than empirical evidence, a situation that may decrease scientific objectivity. Frequently, the rationale behind expert evaluations concerning the relative quality and significance of different types of evidence is inadequately articulated. Nonetheless, these expert judgments are subsequently utilized to arrive at binary or ternary conclusions - “yes,” “no,” or “maybe” - which necessitate distilling complex, detailed biological data into simplistic dichotomous or triadic variables that lack both contextual nuance and clarity regarding their derivation methods. In summary, Krimsky contends that the conclusions drawn from WoE are often the product of a scientifically opaque process characterized by a lack of transparency and an overreliance on subjective assessment.

Rhomberg et al. (2013[55]) reviewed WoE methods and offered recommendations on best practices. The extent to which the methodology employed here avoids the criticisms of Krimsky (2005[36]) and achieves the practice recommendations of Rhomberg et al. (2013[55]) can be evaluated against the epistemic foundation of our method, which has been described previously (Borgert et al., 2011[12][13] Supplemental Materials). To summarize, we discussed three levels of validity targeted by the WoE methodology used here.

### Primary validity: minimal epistemic status

To ensure the validity and reliability of WoE determinations, it is essential to assess the overall quality of the data involved in the evaluation process. Initially, one must consider the minimal epistemic status, referred to as 'primary validity' of the data (Borgert et al., 2011[12]). This entails a thorough examination of each study's results against the fundamental principles of scientific validity as articulated in various commentaries and editorials by Dr. Gio B. Gori, the former Deputy Director of the Division of Cancer Cause and Prevention at the National Cancer Institute (Gori, 1999[32], 2001[31], 2002[28], 2009[27][30], 2010[29]). 

Dr. Gori emphasizes that for data to be regarded as established scientific facts, they must conform to three critical criteria that underpin the fundamental principles of scientific inquiry: First, the identity and authenticity of scientific measurements must be verifiable within a specific range of precision. Second, such measurements and observations must be free from confounding influences that could compromise their accuracy and precision. Third, these measurements and observations must be replicable by independent researchers. 

These three principles are universally acknowledged as the minimum requirements for valid regulatory science in the United States (Subcommittee on Energy and Environment, 2010[66]; Subcommittee on Health, 2010[67]). These principles offer a clear and definitive standard against which all data should be evaluated for their suitability in WoE assessments.

Although disarmingly simple, these three tenets are critically important and powerfully discriminative. To demonstrate the application of tenets, the production of vitellogenin in male fish serves as a prominent example of putative environmental endocrine disruption. The initial step is to ascertain that the study accurately measures what it claims to within a specified range of precision. This fundamental principle inherently promotes a distinction between the measurement itself, and the interpretations attributed to it. In this case, the variable of interest is vitellogenin, typically quantified in blood plasma, though it may also be detected in other tissues such as the liver. Vitellogenin, a dimeric glycolipophosphoprotein, functions as the egg yolk precursor protein across all oviparous vertebrates and can be quantified using several methodologies (e.g., Alda and Barceló, 2001[2]; Wheeler et al., 2005[77]; Wu et al., 2006[80]), each subject to defined margins of error.

Despite its utility as a biomarker, causal relationships between vitellogenin levels and reproductive impairment, or effects on populations, remain unverified. In the absence of such causal links, the presence of vitellogenin in male fish cannot be conclusively associated with "endocrine disruption" (Mills et al., 2003[48]; Mills and Chichester, 2005[47]). Establishing such causality necessitates additional experimental evidence derived from counterfactual study designs. Furthermore, the experimental conditions during which the measurements are taken can be difficult to adequately control. Apart from the methodological controls pertinent to specific analytical techniques, assessments of plasma vitellogenin in male fish must account for baseline levels of the protein within the study population, as well as the influence of viruses known to impact plasma vitellogenin concentrations in both male and female fish (Trubiroha et al., 2010[68]). Additional variables may also need consideration depending on whether the study occurs in a controlled laboratory setting or in the field.

Lastly, it is essential to evaluate whether measurements have been replicated by independent laboratories, which entails diverse investigators utilizing different instruments and personnel. As shown by the cited literature, the assessment of plasma vitellogenin in male fish is generally reproducible in independent laboratories, provided that appropriate experimental and methodological controls and consistent study designs are employed.

### Secondary validity: data reliability and transparency

WoE evaluations must prioritize the reliability of reported data, which we have referred to as 'secondary validity' (Borgert et al., 2011[12]). According to Klimisch et al. (1997[35]), reliability is defined by the transparency and comprehensiveness of data reporting. In the context of *in vivo* studies, they recommend placing more emphasis on studies that provide extensive details regarding the test species, the test substances (including purity and origin), the number of animals studied, the extent of investigations conducted per animal (e.g., clinical chemistry, organ weights, hematology, histopathology), observations of changes or lesions, and relevant control and historical control groups. Additionally, important factors such as test conditions, route of administration, dosage schedule, and dose concentration (along with analytical verification) should be meticulously documented.

For *in vitro* studies, Klimisch et al. assert that greater weight should be given to those that articulate similar particulars concerning the test substances, alongside information pertinent to *in vitro* assays. This includes details about the test system and method, positive and negative controls, potential interferences with the methodology, and data on secondary effects that could sway results (e.g., solubility, impurities, pH fluctuations, osmolarity). Moreover, they emphasize the necessity of similar information in ecotoxicity studies, as well as details regarding the life stages of the animal subjects.

Given the potential for confounding factors in endocrine activity studies, additional elements may be warranted for inclusion in Klimisch's framework. Such considerations could encompass the composition of diets, the materials used for water bottles and cages, bedding, stressors like handling and manipulation, and any other variables that might influence hormonal systems. Furthermore, it is crucial to provide insights into the mathematical and statistical algorithms employed in analyzing the data.

Klimisch et al. (1997[35]) assert that since studies performed under Good Laboratory Practices (GLP) in compliance with regulatory guidelines, referred to as "guideline studies," must document all pertinent information, they should serve as reference standards for assessing reliability. For guideline studies to serve as reference standards, they should either undergo rigorous validation processes, such as those mandated by ICCVAM or ECVAM^1^, or be extensively utilized to ensure their performance is well-documented. Klimisch et al. do not limit their highest reliability rating (code) to guideline studies, but acknowledge that any study that adequately reports on these parameters should be prioritized over studies that do not, regardless of adherence to regulatory guidelines or GLP. Research characterized by enhanced rigor, transparency, and accessibility of documentted data should be seen as more credible regardless of the origin or setting of the study (Schreider et al., 2010[63]). The implementation of uniform, objective criteria, as outlined by Schneider et al. (2009[62]), establishes a scientifically robust foundation for allocating suitable weights to all pertinent toxicity studies, regardless of whether they are GLP or non-GLP. The advantages of GLP have been examined in other sources (Borgert et al., 2016[8]).

While the U.S. EPA's endocrine screening battery includes guideline studies, certain guidelines, like the uterotrophic assay, have undergone rigorous validation programs, whereas others inadequately fulfill the necessary sensitivity and specificity to reliably distinguish between non-endocrine active agents and active ones. Moreover, while their application in the pharmaceutical sector confirms that the binding assays and the uterotrophic and Hershberger assays effectively identify chemicals with significant hormonal activity, it is yet to be established whether these EDSP assays can differentiate weakly hormonal chemicals *in vivo* from false positives, which are substances that generate a signal in the assay but do not exhibit the anticipated hormonal activity *in vivo*. The Tier 1 EDSP cannot be regarded as equally reliable as previous guideline studies until further validation data are obtained (Borgert et al., 2011[12][13]). The U.S. EPA issued testing orders for the screening battery on 67 pesticide compounds, for which substantial reproductive and developmental toxicity data already exist. The initial screening phase may be seen as a validation step, contingent upon the clarity of the data, as the predictive value of the screenings can be assessed by comparing them to the outcomes of definitive guideline studies for these substances. In several cases, the need to revise performance criteria was identified (e.g., Schapaugh et al. 2015[61]).

### Tertiary validity: relevance and probative power of study design and causality

To establish that a chemical acts via a particular endocrine mode of action to produce adverse effects, it is insufficient to rely solely on the correlation between a Tier 1 screening result and an effect observed in Tier 2 testing. The evidential strength of the study design must first be assessed, a characteristic that may be referred to as 'tertiary validity'. Establishing a general causal link between the putative change in endocrine function and a negative outcome is crucial. In this context, an initial demonstration of biological plausibility is necessary, but is itself insufficient to determine a causal mechanistic link. Due to extensive knowledge of the procedures for inducing effects in each Tier 1 EDSP assay, affirmative results might inform the formulation of a preliminary hypothesis concerning the likely mechanism of action. Due to the uncertainty surrounding these steps, it is fitting to characterize this as a "working hypothesis of the mode of action." This will enable the formulation of plausible working hypotheses on the agent's impact on critical processes. After formulating working hypotheses, the causal linkages among the suggested mechanistic processes must be validated by counterfactual experimental procedures. Counterfactual concepts and methodologies for determining causality in pharmacology, toxicology, and epidemiology have been integrated into the assessment of studies utilized in this WoE; however, significant uncertainty persists due to the various issues associated with the necessity of employing excessively high and irrelevant dose levels in toxicology studies (Borgert et al., 2021[10]).

No statistical analysis of literature on EB was conducted to control for publication bias. Tools such as Egger's regression test and funnel plots are commonly employed in systematic reviews and meta-analyses to account for the possibility that negative studies may go unpublished, or that small sample sizes produced false-positives, which would leave the impression that only positive or expected results were obtained. However, this WoE evaluation is not a meta-analysis or a meta-review. Rather, it is a WoE analysis, as required by both the OECD^2^ and the U.S. EPA^3^ for the evaluation of endocrine disruptive potential, and was conducted by a well-established WoE methodology. Much of the data used in this WoE evaluation were from guideline toxicology studies commissioned by the industrial producers of EB in response to regulatory requirements. By statute, the results of such studies must be reported to regulatory agencies irrespective of whether the results are considered “positive” or “negative,” or whether the data are also published in journals. For toxicology studies in general, publication bias towards positive results is the greater risk (McCarty et al., 2012[43]). The possibility of unpublished negative results for EB are a moot issue here because the extant data yield a negative result, notwithstanding. Thus, publication bias towards the negative, as would be addressed by Egger's regressions tests and funnel plots, would not have affected the conclusions of this WoE evaluation.

The assessment of data quality involved the careful selection of studies that fulfilled the inclusion criteria and provided endpoint data that could be ranked by relevance in accordance with the WoE methodology utilized. The studies included underwent evaluation using the established ToxRTool scoring system. In the WoE evaluation, only a single study [11] received a score below 21 (see Appendix A(Tab. 1)). The study's results were categorized based on test species, distinguishing between responses and non-responses for each endpoint. The literature assessed in this WoE evaluation of EB demonstrates adequate quality, allowing for a credible determination with a reasonable level of certainty that the findings were not skewed towards a false-negative conclusion. The conclusions of this evaluation are supported by the systematic parameters applied in the literature and data selection process, the limited data gaps identified in the existing literature, and the objective methodology employed in this WoE assessment, resulting in a high degree of confidence in the findings. Supplementary Table 7 presents a comprehensive overview of the results pertaining to endpoint responses across all six mechanisms of action assessed.

The WoE methodology utilized in this evaluation is distinctive as it ranks the evaluated endpoints based on their significance for testing each hypothesis. Optimally, relevance rankings would rely on empirical evidence that is adequate to compute both positive and negative predictive values. In the absence of such evidence, the rankings used herein represent the interpretations made by an expert panel, grounded in empirical observations of endpoint responses to established positive and negative controls for each mechanism of action (Borgert et al., 2014[10]). The rankings vary mainly in terms of the specificity and sensitivity of the endpoints related to the hypothesis being examined. The assessment of specificity was based on two criteria: first, the extent to which the endpoint accurately represents a response of the physiological system to the mechanism of action (MoA) being evaluated; and second, the extent to which the endpoint can respond to MoAs that differ from the one under investigation, particularly those that are non-endocrine in nature. This is a significant factor to consider in any assessment of endocrine mechanisms of action, as the endocrine system plays a crucial homeostatic role in nearly every system it regulates, including primary biological functions like growth, development, reproduction, and metabolism. As a result, endocrine pathways influence and are influenced by various non-endocrine physiological and biochemical processes. Identifying direct and indirect effects fundamentally depends on comprehending the specificity of the response.

This WoE methodology addresses specificity by requiring an evaluation of the response patterns associated with each MoA. Patterns that deviate from the expected responses of known effectors and inhibitors are interpreted as unlikely to signify activity through that pathway. It is theoretically possible for an agonist or antagonist of a specific hormonal pathway to demonstrate a novel pattern of endpoint responses, aligning with the “selective response modifier” concept of hormone receptor interactions. However, a limited number of response types have been identified across various hormone receptor systems, each corresponding to ligand-receptor affinities, potencies, and the tissue distribution of hormone receptors (see Borgert et al., 2018[11] and references therein). Assessment approaches that do not evaluate this fundamental aspect of hormonal action, such as the key characteristic approach proposed by La Merrill et al. (2020[38]), have little utility for identifying potential endocrine disruptors (Borgert, 2023[7]).

It is reasonable to consider the methodology's effectiveness for a chemical that elicits responses in more endpoints pertinent to a specific hormonal mechanism of action compared to EB, especially in Rank 1 and Rank 2 endpoints. The response patterns anticipated for each mechanism of action necessitate detailed examination, as do the possible influences of competing mechanisms on the endpoints, including hormonal mechanisms beyond the one being evaluated. While there exists potential for ligand-receptor interactions among various hormonal receptors, receptor specificity remains a significant characteristic of the endocrine system. For example, although androgens can bind and activate estrogen receptors at high concentrations, they do not function as potent estrogens, and therefore, it is unlikely for a chemical to demonstrate action by both modes concurrently. When responses arise from different hormonal MoAs, it is essential to consider the possibility of systemic toxicity as a common underlying factor.

A thorough examination of response weightings, as suggested by Borgert et al. (2014[14]), is likely essential for evaluation of chemicals that produce responses in many endpoints, despite the limited discussion here due to the absence of a relevant response pattern to EB. The application of response weighting to elucidate the endocrine-disruptive potential of a chemical exhibiting weak responses in Rank 1 and Rank 2 endpoints has been documented for octamethylcyclotetrasiloxane (Borgert and Burgoon, 2025[9]; Borgert et al., 2018[11]; Matthews, 2021[42]). Response weighting pertains to mechanistic potency, a core principle of receptor, enzyme, and transport kinetics relevant to the interactions between biological macromolecules and all small molecules, regardless of their endogenous or exogenous nature.

The data concerning the potential endocrine activity of EB assessed in this study indicate that it does not exhibit a pattern of results aligned with EATS MoAs and demonstrates minimal or negligible potential for interaction with EATS pathways. The lack of potential for EB to operate through EATS MoAs is evidenced by the inconsistent responses observed in endpoints pertinent to each EATS hypothesis, as well as the absence of a response pattern suggestive of any MoA. Supplementary Tables 1 to 6 present a range of results concerning endpoints associated with potential EATS activity. Endpoint responses are rarely reported in isolation across studies; when they are, such responses typically occur at high doses, which are complicated by systemic toxicity and other MoAs. The endpoint response patterns to EB, as indicated in Supplementary Tables 1-6, do not align with EATS activity. The absence of a discernible pattern suggesting an endocrine response among the MoAs assessed is significant. While some variability among endocrine modulators is anticipated due to the potential for selective endocrine response modifiers (e.g., Kuiper et al. 1999[37]), even selective responses should not be random or inconsistent with an endocrine mechanism, unlike the responses noted with EB.

The data employed in this WoE evaluation of EB are primarily from repeat dose, sub-chronic, and chronic toxicity studies, i.e., OSRI, rather than from the mechanistic screening assays included in the U.S. EPA's original EDSP Tier 1 and the lower tiers of the OECD Toolbox (U.S. EPA, 2009[71]; OECD, 2012[54]). This WoE evaluation for EB indicates that further EDSP Tier Screens or OECD screening-level assays would not provide additional valuable information, as any responses observed would merely prompt the types of studies and endpoint measurements assessed in this evaluation, which demonstrate a lack of potential endocrine activity through EATS MoAs. Moreover, as only four studies examining the reproductive and developmental effects of EB were conducted prior to 2001, the dataset includes endpoints that are sensitive to adverse effects potentially arising from endocrine mechanisms. Consequently, the Tier 2 data remain relevant and comprehensive. The existing studies on reproductive, developmental, and repeat dose toxicology have evaluated the life stages deemed most pertinent for assessing the adverse effects of EB that may result from any mode of action, including endocrine disruption. Ecological effects are not expected based on the applications of EB, its physicochemical characteristics, and its environmental behavior and degradation. The limited potential for obtaining useful information from further endocrine screening and testing of EB suggests that justifying the use of additional animals for this purpose is unwarranted.

The measurement of apical endpoints in the repeat dose, sub-chronic, and chronic reproductive toxicity studies with EB is of particular significance, as it encompasses many Rank 2 endpoints evaluated in this context. Responses in highly specific endocrine screening assays (Rank 1) are not conclusive; they suggest potential activity that requires further investigation through definitive, long-term studies. Apical endpoints, on the other hand, indicate possible adverse effects that may arise from either endocrine or non-endocrine pathways. A lack of response in apical endpoints is more informative and should be prioritized over a response. The absence of response in apical endpoints anticipated to react to endocrine modulators operating through EATS pathways rules out both an endocrine mechanism of action (MoA) and a non-endocrine MoA that indirectly influences the endocrine system. Conversely, a response suggests the potential existence of an underlying endocrine MoA for the observed effect. The significant absence of response to EB in these apical endpoints further emphasizes the strong evidence indicating that EB does not possess endocrine activity or endocrine disruptive potential.

Finally, the fact that only four endpoints responded to EB exposure at levels below the KMD for EB of 200 ppm (Burgoon et al., 2023[16]) underscores that the conclusions reached here are highly conservative. Most endpoint responses were observable only above the KMD, making it highly likely that the MoA by which they arise involves high-dose-dependent changes in the kinetics of EB, in effect, kinetic overload of the organism. Since exposure levels below the KMD do not produce kinetic overload, responses observed only at exposure levels higher than the KMD do not portend similar effects at doses below the KMD, nor should they be used for risk assessment or for making inferences about endocrine disruptive potential.

## Conclusions

Using an objective, pre-defined WoE methodology, there is high confidence in the conclusion that EB lacks the potential to produce adverse effects through (anti)estrogen, (anti)androgen, thyroid, or steroidogenic pathways. This conclusion is obligate because there is an inconsistency across studies among the few endpoints that responded to EB, but a high consistency among endpoints that did not respond to EB:

100 % failed to respond as an estrogen agonist; 19 % responded inconsistently.

100 % failed to respond as an estrogen antagonist; 14.3 % responded inconsistently.

100 % failed to respond as an androgen agonist; 21.1 % responded inconsistently.

100 % failed to respond as an androgen antagonist; 16.7 % responded inconsistently.

91 % failed to respond as a thyroid inhibitor; 50.0 % responded inconsistently.

100 % failed to respond as steroidogenesis inhibitor; 9.1 % responded inconsistently.

Given that EB demonstrates no potential to act through EATS pathways, it is biologically implausible for any adverse effects of EB to arise via these endocrine MoAs. While various endocrine mechanisms of action exist, there is insufficient evidence to support a contention that EB functions through endocrine mechanisms not assessed in this study. Consequently, EB cannot be classified as an endocrine disruptor, a potential endocrine disruptor, or as possessing endocrine disruptive properties according to an objective assessment of the available data. Thus, additional endocrine screening of EB would be an inefficient use of resources and animals, raising ethical concerns regarding animal welfare at this time. Further endocrine testing of EB is unnecessary unless new and compelling evidence for alternative endocrine mechanisms of action is presented. Although this evaluation for EB concentrated on OSRI as defined by the U.S. EPA's EDSP, the findings are also pertinent to the regulatory assessment of endocrine disruptors in the EU, which aims to identify chemicals that meet the WHO/IPCS definition for labeling as “endocrine disruptors.”

## Notes

^1^ Interagency Coordinating Committee on the Validation of Alternative Methods (ICCVAM) and European Centre for Validation of Alternative Methods (ECVAM)

^2^ OECD (2018[54]), Revised Guidance Document 150 on Standardised Test Guidelines for Evaluating Chemicals for Endocrine Disruption, OECD Series on Testing and Assessment, OECD Publishing, Paris. https://doi.org/10.1787/9789264304741-en

^3^ EPA US. (2011[73]). Weight-of-Evidence: Evaluating Results of EDSP Tier 1 Screening to Identify the Need for Tier 2 Testing. Office of Chemical Safety and Pollution Prevention.

## Declaration

### Acknowledgments

The author thanks Kathleen C. Findlay, M.S., PharmD, for initial extraction of data, ToxRTool evaluations, and compilation of data summaries by endpoint; Susan A. Borgert, R.Ph., M.S., CPh, for compilation of data tables, manuscript formatting and proof-reading; Janice R. Ballo, M.A., MLS. for consultation on literature search strategies, conducting literature searches, and manuscript proof-reading and formatting.

### Author roles

C.J. Borgert devised the methodologies used (see previous Borgert et al., publications, as cited), directed the literature searches, selected the literature, evaluated the literature, directed data compilation into data tables, directed literature summaries contained in Supplemental Materials, composed the manuscript, and responded to peer-review of the manuscript submissions.

### Declaration of interest

Disclosures for this publication are identical to those for the WoE evaluation of styrene, published previously (Borgert, 2023[6]).

Disclosure: Funding and conflict of interest disclosures for this publication are identical to those published previously (Borgert, 2023[6]).

## Supplementary Material

Supplementary information

## Figures and Tables

**Table 1:**
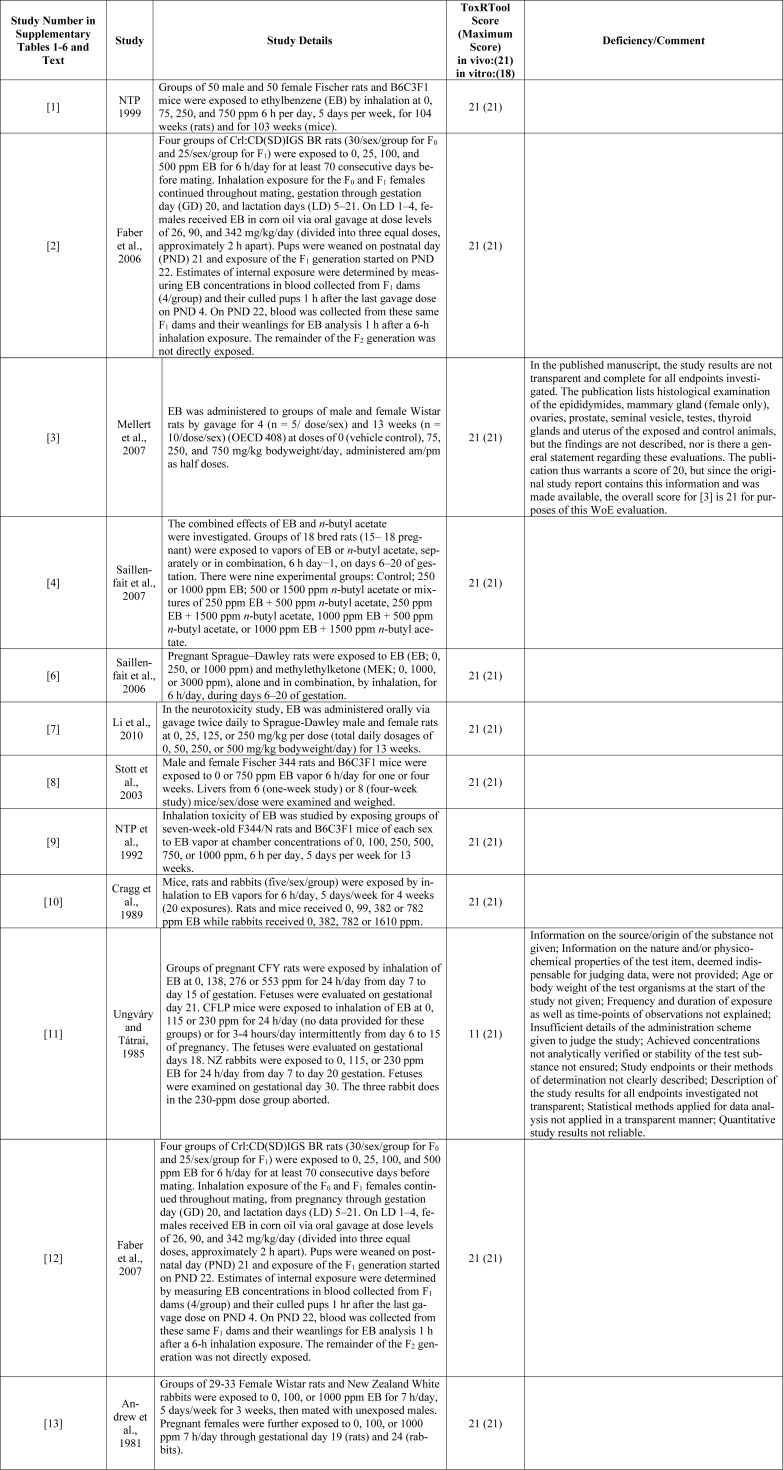
Appendix A: ToxRTool Summary

**Figure 1:**
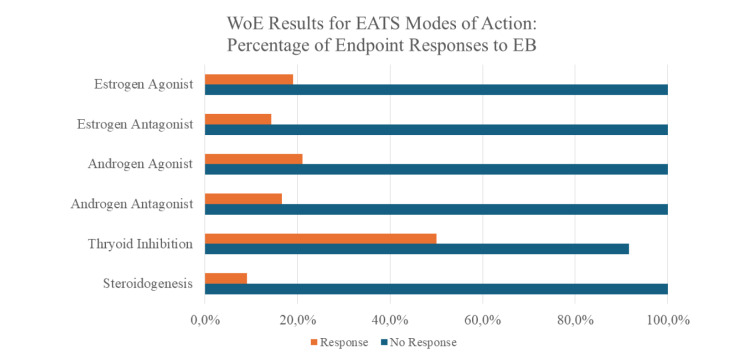
Graphical abstract

## References

[1] Afarinesh MR, Shafiei F, Sabzalizadeh M, Haghpanah T, Taheri M, Parsania S (2020). Effect of mild and chronic neonatal hypothyroidism on sensory information processing in a rodent model: A behavioral and electrophysiological study. Brain Res Bull.

[2] Alda M, Barceló D (2001). Review of analytical methods for the determination of estrogens and progestogens in waste waters. Fresenius J Anal Chem.

[3] Andrew FD, Buschbom RL, Cannon WC, RA M, Montgomery LF, Phelp DW, et al. (1981). Teratologic assessment of ethylbenzene and 2-ethoxyethanol.

[4] Andrews P, Freyberger A, Hartmann E, Eiben R, Loof I, Schmidt U (2002). Sensitive detection of the endocrine effects of the estrogen analogue ethinylestradiol using a modified enhanced subacute rat study protocol (OECD Test Guideline no. 407). Arch Toxicol.

[5] Biegel LB, Flaws JA, Hirshfield AN, O’Connor JC, Elliott GS, Ladics GS (1998). 90-day feeding and one-generation reproduction study in Crl:CD BR rats with 17 beta-estradiol. Toxicol Sci.

[6] Borgert CJ (2023). Hypothesis-driven weight of evidence evaluation indicates styrene lacks endocrine disruption potential. Crit Rev Toxicol.

[7] Borgert CJ (2023). Issue analysis: key characteristics approach for identifying endocrine disruptors. Arch Toxicol.

[8] Borgert CJ, Becker RA, Carlton BD, Hanson M, Kwiatkowski PL, Sue Marty M (2016). Does GLP enhance the quality of toxicological evidence for regulatory decisions?. Toxicol Sci.

[9] Borgert CJ, Burgoon LD (2025). Octamethylcyclotetrasiloxane (D4) lacks endocrine disruptive potential via estrogen pathways. Arch Toxicol.

[10] Borgert CJ, Fuentes C, Burgoon LD (2021). Principles of dose-setting in toxicology studies: the importance of kinetics for ensuring human safety. Arch Toxicol.

[11] Borgert CJ, Matthews JC, Baker SP (2018). Human-relevant potency threshold (HRPT) for ERα agonism. Arch Toxicol.

[12] Borgert CJ, Mihaich EM, Ortego LS, Bentley KS, Holmes CM, Levine SL (2011). Hypothesis-driven weight of evidence framework for evaluating data within the US EPA’s Endocrine Disruptor Screening Program. Regul Toxicol Pharmacol.

[13] Borgert CJ, Mihaich EM, Quill TF, Marty MS, Levine SL, Becker RA (2011). Evaluation of EPA’s Tier 1 Endocrine Screening Battery and recommendations for improving the interpretation of screening results. Regul Toxicol Pharmacol.

[14] Borgert CJ, Stuchal LD, Mihaich EM, Becker RA, Bentley KS, Brausch JM (2014). Relevance weighting of Tier 1 endocrine screening endpoints by rank order. Birth Defects Res Dev Reprod Toxicol.

[15] Browne P, Judson RS, Casey WM, Kleinstreuer NC, Thomas RS (2015). Screening chemicals for estrogen receptor bioactivity using a computational model. Environ Sci Technol.

[16] Burgoon LD, Borgert CJ, Fuentes C, Klaunig JE (2023). Kinetically-derived maximal dose (KMD) indicates lack of human carcinogenicity of ethylbenzene. Arch Toxicol.

[17] Burgoon LD, Fuentes C, Borgert CJ (2022). A novel approach to calculating the kinetically derived maximum dose. Arch Toxicol.

[18] Cragg ST, Clarke EA, Daly IW, Miller RR, Terrill JB, Ouellette RE (1989). Subchronic inhalation toxicity of ethylbenzene in mice, rats, and rabbits. Fund Appl Toxicol.

[19] Delclos KB, Weis CC, Bucci TJ, Olson G, Mellick P, Sadovova N (2009). Overlapping but distinct effects of genistein and ethinyl estradiol (EE(2)) in female Sprague-Dawley rats in multigenerational reproductive and chronic toxicity studies. Reprod Toxicol.

[20] EDSTAC, Endocrine Disruptor Screening and Testing Advisory Committee (1998). Endocrine Disruptor Screening and Testing Advisory Committee (EDSTAC) Final Report.

[21] European Parliament (2012). Council of the European Union. BPR (Biocidal Products Regulation), Regulation (EU). 2012;528. Off J Eur Union.

[22] European Parliament (2006). Council of the European Union. REACH (Registration, Evaluation, Authorization and Restriction of Chemicals), Regulation (EC) No 1907/ 2006. Off J Eur Union.

[23] Faber WD, Roberts LS, Stump DG, Tardif R, Krishnan K, Tort M (2006). Two generation reproduction study of ethylbenzene by inhalation in crl-cd rats. Birth Defects Res B Dev Reprod Toxicol.

[24] Faber, WD, Roberts LS, Stump, DG, Beck, M, Kirkpatrick D, Regan KS (2007). Inhalation developmental neurotoxicity study of ethylbenzene in Crl-CD rats. Birth Defects Res B Dev Reprod Toxicol.

[25] Gong X, Huang Y, Duong J, Leng S, Zhan FB, Guo Y (2023). Industrial air pollution and low birth weight in New Mexico, USA. J Environ Manage.

[26] Gong X, Lin Y, Bell ML, Zhan FB (2018). Associations between maternal residential proximity to air emissions from industrial facilities and low birth weight in Texas, USA. Environ Int.

[27] Gori GB (2009). Conflict of interest and public policy. Regul Toxicol Pharmacol.

[28] Gori GB (2002). Considerations on guidelines of epidemiologic practice. Ann Epidemiol.

[29] Gori GB (2010). Regulating unknown risk. Regulation.

[30] Gori GB (2009). Scientific integrity. Regul Toxicol Pharmacol.

[31] Gori GB (2001). The costly illusion of regulating unknowable risks. Regul Toxicol Pharmacol.

[32] Gori GB (1999). The EPA and the courts: inching toward a showdown. Regul Toxicol Pharmacol.

[33] Harrath AH, Alrezaki A, Jalouli M, Aldawood N, Aldahmash W, Mansour L (2022). Ethylbenzene exposure disrupts ovarian function in Wistar rats via altering folliculogenesis and steroidogenesis-related markers and activating autophagy and apoptosis. Ecotoxicol Environ Saf.

[34] Kleinstreuer NC, Ceger P, Watt ED, Martin M, Houck K, Browne P (2017). Development and validation of a computational model for androgen receptor activity. Chem Res Toxicol.

[35] Klimisch HJ, Andreae M, Tillmann U (1997). A systematic approach for evaluating the quality of experimental toxicological and ecotoxicological data. Regul Toxicol Pharmacol.

[36] Krimsky S (2005). The weight of scientific evidence in policy and law. Am J Public Health.

[37] Kuiper GG, van den Bemd GJ, van Leeuwen JP (1999). Estrogen receptor and the SERM concept. J Endocrinol Invest.

[38] La Merrill MA, Vandenberg LN, Smith MT, Goodson W, Browne P, Patisaul, HB (2020). Consensus on the key characteristics of endocrine-disrupting chemicals as a basis for hazard identification. Nat Rev Endocrinol.

[39] Lei T, Qian H, Yang J, Hu Y (2023). The association analysis between exposure to volatile organic chemicals and obesity in the general USA population: A cross-sectional study from NHANES program. Chemosphere.

[40] Li AA, Maurissen JP, Barnett JF, Foss J, Freshwater L, Garman RH (2010). Oral gavage subchronic neurotoxicity and inhalation subchronic immunotoxicity studies of ethylbenzene in the rat. Neurotoxicology.

[41] Marty MS, Borgert C, Coady K, Green R, Levine SL, Mihaich E (2018). Distinguishing between endocrine disruption and non-specific effects on endocrine systems. Regul Toxicol Pharmacol.

[42] Matthews JC (2021). A mechanistic evaluation of the potential for octamethylcyclotetrasiloxane to produce effects via endocrine modes of action. Crit Rev Toxicol.

[43] McCarty LS, Borgert CJ, Mihaich EM (2012). Information quality in regulatory decision-making: peer review versus good laboratory practice. Environ Health Perspect.

[44] Mellert W, Deckardt K, Kaufmann W, van Ravenzwaay B (2007). Ethylbenzene: 4-and 13-week rat oral toxicity. Arch Toxicol.

[45] Mihaich E, Capdevielle M, Urbach-Ross D, Slezak B (2017). Hypothesis-driven weight-of-evidence analysis of endocrine disruption potential: a case study with triclosan. Crit Rev Toxicol.

[46] Mihaich EM, Borgert CJ (2018). Hypothesis-driven weight-of-evidence analysis for the endocrine disruption potential of benzene. Regul Toxicol Pharmacol.

[47] Mills LJ, Chichester C (2005). Review of evidence: Are endocrine-disrupting chemicals in the aquatic environment impacting fish populations?. Sci Total Environ.

[48] Mills LJ, Gutjahr-Gobell RE, Horowitz DB, Denslow ND, Chow MC, Zaroogian GE (2003). Relationship between reproductive success and male plasma vitellogenin concentrations in cunner, Tautogolabrus adspersus. Environ Health Perspect.

[49] Nakhjirgan P, Kashani H, Naddafi K, Nabizadeh R, Amini H, Yunesian M (2019). Maternal exposure to air pollutants and birth weight in Tehran, Iran. J Environ Health Sci Eng.

[50] Neal BH, Bus J, Marty MS, Coady K, Williams A, Staveley J (2017). Weight-of-the-evidence evaluation of 2,4-D potential for interactions with the estrogen, androgen and thyroid pathways and steroidogenesis. Crit Rev Toxicol.

[51] NTP (National Toxicology Program) (Research). Multigenerational reproductive toxicology study of ethinyl estradiol (CAS No. 57-63-6) in Sprague-Dawley rats. NTP TR 547.

[52] NTP (National Toxicology Program) (1992). Toxicity studies of ethylbenzene (CAS No. 100-41-4) in F344/N rats and B6C3F1 mice (inhalation studies). NTP Tox. 10. NIH Publication No. 92-3129. PB93-149722.

[53] NTP (National Toxicology Program) (1999). Toxicology and carcinogenesis studies of ethylbenzene (CAS No. 100-41-4) in F344/N rats and B6C3F1 mice (Inhalation Studies). NTP Tech Rep Ser.

[54] OECD (Organisation for Economic Co-operation and Development) (Paris). Guidance document on standardised test guidelines for evaluating chemicals for endocrine disruption. ENV/JM/MONO(2012)22.

[55] Rhomberg LR, Goodman JE, Bailey LA, Prueitt RL, Beck NB, Bevan C (2013). A survey of frameworks for best practices in weight-of-evidence analyses. Crit Rev Toxicol.

[56] Rotroff DM, Dix DJ, Houck KA, Knudsen TB, Martin MT, McLaurin KW (2013). Using in vitro high throughput screening assays to identify potential endocrine-disrupting chemicals. Environ Health Perspect.

[57] Rouget F, Bihannic A, Cordier S, Multigner L, Meyer-Monath M, Mercier F (2021). Petroleum and chlorinated solvents in meconium and the risk of hypospadias: a pilot study. Front Pediatr.

[58] Saillenfait AM, Gallissot F, Morel G, Bonnet P (2003). Developmental toxicities of ethylbenzene, ortho-, meta-, para-xylene and technical xylene in rats following inhalation exposure. Food Chem Toxicol.

[59] Saillenfait AM, Gallissot F, Sabaté JP, Bourges-Abella N, Cadot R, Morel G (2006). Developmental toxicity of combined ethylbenzene and methylethylketone administered by inhalation to rats. Food Chem Toxicol.

[60] Saillenfait AM, Gallissot F, Sabate JP, Bourges-Abella N, Muller S (2007). Developmental toxic effects of ethylbenzene or toluene alone and in combination with butyl acetate in rats after inhalation exposure. J Appl Toxicol.

[61] Schapaugh AW, McFadden LG, Zorrilla LM, Geter DR, Stuchal LD, Sunger N (2015). Analysis of EPA’s endocrine screening battery and recommendations for further review. Regul Toxicol Pharmacol.

[62] Schneider K, Schwarz M, Burkholder I, Kopp-Schneider A, Edler L, Kinsner-Ovaskainen A (2009). “ToxRTool”, a new tool to assess the reliability of toxicological data. Toxicol Lett.

[63] Schreider J, Barrow C, Birchfield N, Dearfield K, Devlin D, Henry S (2010). . Enhancing the credibility of decisions based on scientific conclusions: transparency is imperative. Toxicol Sci.

[64] Slikker W, Andersen ME, Bogdanffy MS, Bus JS, Cohen SD, Conolly RB (2004). Dose-dependent transitions in mechanisms of toxicity. Toxicol Appl Pharmacol.

[65] Stott WT, Johnson KA, Bahnemann R, Day SJ, McGuirk RJ (2003). Evaluation of potential modes of action of inhaled ethylbenzene in rats and mice. Toxicol Sci.

[66] Subcommittee on Energy and Environment, US House of Representatives, Committee on Energy and Commerce (2010). Endocrine-disrupting chemicals in drinking water: risks to human health and the environment. Hearing before the Subcommittee on Energy and Environment of the Committee on Energy and Commerce, House of Representatives, 111 Congress, 2nd session, February 25, 2010. Serial No. 111-99. https://www.govinfo.gov/content/pkg/CHRG-111hhrg76011/pdf/CHRG-111hhrg76011.pdf.

[67] Subcommittee on Health, US House of Representatives Committee on Energy and Commerce (2012). Hearing report of 4/22/2010; Hearing Transcript at pages 79 & 80. 111 Congress, 2nd session, April 22, 2010. Serial No. 111-112. https://www.govinfo.gov/content/pkg/CHRG-111hhrg76567/pdf/CHRG-111hhrg76567.pdf.

[68] Trubiroha A, Kroupova H, Wuertz S, Frank SN, Sures B, Kloas W (2010). Naturally-induced endocrine disruption by the parasite Ligula intestinalis (Cestoda) in roach (Rutilus rutilus). Gen Comp Endocrinol.

[69] Ungváry G, Tátrai E (1985). On the embryotoxic effects of benzene and its alkyl derivatives in mice, rats and rabbits. Arch Toxicol. Arch Toxicol.

[70] US EPA, Environmental Protection Agency (2023). Endocrine Disruptor Screening Program (EDSP); Near-Term Strategies for Implementation; Notice of Availability and Request for Comment. EPA-HQ-OPP-2023-0474; FRL-11384-01-OCSPP.

[71] US EPA, Environmental Protection Agency (2009). Environmental Protection Agency. Endocrine Disruptor Screening Program: Tier 1 Screening Order Issuing Announcement. 74 FR 54422, Doc. No. E9-25352, Oct. 21, 2009. https://www.federalregister.gov/documents/2009/10/21/E9-25352/endocrine-disruptor-screening-program-tier-1-screening-order-issuing-announcement.

[72] US EPA, Environmental Protection Agency (2009). Environmental Protection Agency. Final contaminant candidate list 3 chemicals: Screening to a PCCL. EPA 815-R-09-007, August 2009. https://www.epa.gov/sites/default/files/2014-05/documents/ccl3chem_screening_to_pccl_08-31-09_508v2.pdf.

[73] US EPA, Environmental Protection Agency (2011). Evaluating results of EDSP Tier 1 Screening to Identify the Need for Tier 2 Testing. EPA-HQ-OPPT-2013-0275-0004.

[74] US Public Law 114-182 (2016). Frank R. Lautenberg Chemical Safety for the 21st Century Act, 15 U.S.C. § 2601 et seq. 15 U.S.C. 2603, Sec. 4. Testing of Chemical substances and Mixtures. https://www.congress.gov/114/plaws/publ182/PLAW-114publ182.pdf.

[75] Werder EJ, Beier JI, Sandler DP, Falkner KC, Gripshover T, Wahlang B (2020). Blood BTEXS and heavy metal levels are associated with liver injury and systemic inflammation in Gulf states residents. Food Chem Toxicol.

[76] Werder EJ, Engel LS, Blair A, Kwok RK, McGrath JA, Sandler DP (2019). Blood BTEX levels and neurologic symptoms in Gulf states residents. Environ Res.

[77] Wheeler JR, Gimeno S, Crane M, Lopez-Juez E, Morritt D (2005). Vitellogenin: A review of analytical methods to detect (anti) estrogenic activity in fish. Toxicol Mech Meth.

[78] WHO, World Health Organization, IPCS, International Program on Chemical Safety (2002). Global assessment of the state-of-the-science of endocrine disruptors. WHO/ PCS/EDC/02.2.

[79] WHO, World Health Organization, UNEP, United Nations Environment Program (2012). State of the science of endocrine disrupting chemicals.

[80] Wu C, Yuan D, Liu B (2006). Rapid determination of vitellogenin in fish plasma by anion exchange high performance liquid chromatography using postcolumn fluorescence derivatization with o-phthalaldehyde. Anal Sci.

