# Supplementary material for: Hypothesis-driven weight of evidence evaluation indicates ethylbenzene lacks endocrine disruption potential by EATS pathways
Source: EXCLI J. 2025 Mar 27;24:479–507. doi: 10.17179/excli2024-7822 (PMC12078780; doi:10.17179/excli2024-7822)
Supplement: Supplementary information [file EXCLI-24-479-s-001.pdf]

EXCLI Journal 2025;24:479-507 – ISSN 1611-2156
Received: September 13, 2024, accepted: February 25, 2025, published: March 27, 2025

Supplementary information to:

Original article:

HYPOTHESIS-DRIVEN WEIGHT OF EVIDENCE EVALUATION INDICATES ETHYLBENZENE LACKS ENDOCRINE DISRUPTION POTENTIAL BY EATS PATHWAYS

Christopher J. Borgert, PhD

Applied Pharmacology and Toxicology Inc, Gainesville FL, 32605 and
University of Florida College of Veterinary Medicine, Dept. Physiological Sciences, Gainesville FL, 32610. Tel.: +1 352-219-8551

https://dx.doi.org/10.17179/excli2024-7822

This is an Open Access article distributed under the terms of the Creative Commons Attribution License (http://creativecommons.org/licenses/by/4.0/).

Supplemental material A: Ethylbenzene references reviewed
Supplemental material B: OSRI evaluation for ethylbenzene – Summaries of studies
Supplemental material C: Rationale for excluding studies
Supplementary Table 1: Estrogen agonist hypothesis; guideline toxicity studies
Supplementary Table 2: Estrogen antagonist hypothesis; guideline toxicity studies
Supplementary Table 3: Androgen agonist hypothesis; guideline toxicity studies
Supplementary Table 4: Androgen antagonist hypothesis; guideline toxicity studies
Supplementary Table 5: Thyroid inhibition hypothesis; guideline toxicity studies
Supplementary Table 6: Interaction with steroidogenesis enzymes hypothesis; guideline toxicity studies
Supplementary Table 7: Summary of endpoints from all tables

Ethylbenzene References Reviewed
Supplemental Material A

Acuna-Askar, K., Villarreal-Chiu, J.F., Gracia-Lozano, M.V., Garza-Gonzalez, M.T., Chavez-Gomez, B., Rodriguez-Sanchez, I.P., and Barrera-Saldana, H.A. (2004). BTE-OX biodegradation kinetics with MTBE through bioaugmentation. Water Sci Technol 50, 85-92

Adebambo, T. H., Fox, D. T., & Otitoloju, A. A. (2020). Toxicological Study and Genetic Basis of BTEX Susceptibility in Drosophila melanogaster. Front Genet, 11, 594179. https://doi.org/10.3389/fgene.2020.594179

Aguilera, I., Garcia-Esteban, R., Iñiguez, C., Nieuwenhuijsen, M.J., Rodríguez, A., Paez, M., Ballester, F., and Sunyer, J. (2010). Prenatal exposure to traffic-related air pollution and ultrasound measures of fetal growth in the INMA Sabadell cohort. Environ Health Perspect 118, 705-711.

Aguilera, I., Guxens, M., Garcia-Esteban, R., Corbella, T., Nieuwenhuijsen, M.J., Foradada, C.M., and Sunyer, J. (2009). Association between GIS-based exposure to urban air pollution during pregnancy and birth weight in the INMA Sabadell Cohort. Environ Health Perspect 117, 1322-27.

Ahmadi, Z., Moradabadi, A., Abdollahdokht, D., Mehrabani, M., & Nematollahi, M. H. (2019). Association of environmental exposure with hematological and oxidative stress alteration in gasoline station attendants. Environ Sci Pollut Res Int, 26(20), 20411-20417.

Akdeniz, N., Jacobson, L.D., and Hetchler, B.P. (2013). Health risk assessment of occupational exposure to hazardous volatile organic compounds in swine gestation, farrowing and nursery barns. Environ Sci Process Impacts 15, 563-572.

Alabdulhadi, A., Ramadan, A., Devey, P., Boggess, M., & Guest, M. (2019). Inhalation exposure to volatile organic compounds in the printing industry. J Air Waste Manag Assoc, 69(10), 1142-1169.

Andrew, F. D; Buschbom, R.L.; Cannon, W.C.; Miller, R.A.; Montgomery, L.F.; Phelp, D.W.; Sikov, M.R. 1981: Teratologic Assessment of Ethylbenzene and 2-Ethoxyethanol (publication), NTIS Report No. PB83-208074.

ATSDR 2010. Toxicological Profile for Ethylbenzene. November 2010. U.S. department of health and human services Public Health Service Agency for Toxic Substances and Disease Registry.

Baines, C.J., McKeown-Eyssen, G.E., Riley, N., Cole, D.E., Marshall, L., Loescher, B., and Jazmaji, V. (2004). Case-control study of multiple chemical sensitivity, comparing haematology, biochemistry, vitamins and serum volatile organic compound measures. Occup Med (Lond) 54, 408-418.

Berthet, A., de Batz, A., Tardif, R., Charest-Tardif, G., Truchon, G., Vernez, D., and Droz, P.O. (2010). Impact of biological and environmental variabilities on biological monitoring--an approach using toxicokinetic models. J Occup Environ Hyg 7, 177-184.

Bolden AL, Kwiatkowski CF, Colborn T. 2015. New Look at BTEX: Are Ambient Levels a Problem. Environ Sci Technol 49: 5261–76.

Bolden AL, Schultz K, Pelch KE, Kwiatkowski CF. 2018. Exploring the endocrine activity of air pollutants associated with unconventional oil and gas extraction. Environ Health 17: 26.

Boyle EB, Viet SM, Wright DJ, Merrill LS, Alwis KU, Blount BC, Mortensen ME, Moye JJ, Dellarco M. 2016. Assessment of Exposure to VOCs among Pregnant Women in the National Children's Study. Int J Environ Res Public Health 13: 376.

Brajenovic N, Karaconji IB, Bulog A. 2015. Evaluation of Urinary Btex, Nicotine & Cotinine as Biomarkers of Airborne Pollutants in Nonsmokers & Smokers. J Toxicol Environ Health A 78:1133-6.

Ethylbenzene References Reviewed
Supplemental Material A

Brennan, R.J., Kandikonda, S., Khrimian, A.P., DeMilo, A.B., Liquido, N.J., and Schiestl, R.H. (1996). Saturated and monofluoro analogs of the oriental fruit fly attractant methyl eugenol show reduced genotoxic activities in yeast. Mutat Res 369, 175-181.

Brown DR, Lewis C, Weinberger BI. 2015. Human exposure to unconventional natural gas development: A public health demonstration of periodic high exposure to chemical mixtures in ambient air. J Environ Sci Health A Tox Hazard Subst Environ Eng 50: 460–72.

Cao, Y. M., Gao, W. M., & Liu, J. (2018). [Study on the health effects of occupational exposure to low concentrations of benzene]. Zhonghua Lao Dong Wei Sheng Zhi Ye Bing Za Zhi, 36(6), 435-438.

Capella, K. M., Roland, K., Geldner, N., Rey deCastro, B., De Jesús, V. R., van Bemmel, D., & Blount, B. C. (2019). Ethylbenzene and styrene exposure in the United States based on urinary mandelic acid and phenylglyoxylic acid: NHANES 2005-2006 and 2011-2012. Environ Res, 171, 101-110.

Cardozo, T.R., Rosa, D.P., Feiden, I.R., Rocha, J.A., de Oliveira, N.C., da Silva Pereira, T., Pastoriza, T.F., da Motta Marques, D., de Lemos, C.T., et al. (2006). Genotoxicity and toxicity assessment in urban hydrographic basins. Mutat Res 603, 83-96.

Caron-Beaudoin, É., Whitworth, K. W., Bosson-Rieutort, D., Wendling, G., Liu, S., & Verner, M. A. (2021). Density and proximity to hydraulic fracturing wells and birth outcomes in Northeastern British Columbia, Canada. J Expo Sci Environ Epidemiol, 31(1), 53-61.

Caron-Beaudoin, É., Whyte, K. P., Bouchard, M. F., Chevrier, J., Haddad, S., Copes, R., Frohlich, K. L., Dokkie, D., Juul, S., Bouchard, M., & Verner, M. A. (2022). Volatile organic compounds (VOCs) in indoor air and tap water samples in residences of pregnant women living in an area of unconventional natural gas operations: Findings from the EXPERIVA study. Sci Total Environ, 805, 150242.

Casey, J. A., Goin, D. E., Rudolph, K. E., Schwartz, B. S., Mercer, D., Elser, H., Eisen, E. A., & Morello-Frosch, R. (2019). Unconventional natural gas development and adverse birth outcomes in Pennsylvania: The potential mediating role of antenatal anxiety and depression. Environ Res, 177, 108598.

Cassidy-Bushrow, A. E., Burmeister, C., Birbeck, J., Chen, Y., Lamerato, L., Lemke, L. D., Li, J., Mor, G., O'Leary, B. F., Peters, R. M., Reiners, J. J. J., Sperone, F. G., Westrick, J., Wiewiora, E., & Straughen, J. K. (2021). Ambient BTEX exposure and mid-pregnancy inflammatory biomarkers in pregnant African American women. J Reprod Immunol, 145, 103305.

Cassidy-Bushrow, A. E., Burmeister, C., Lamerato, L., Lemke, L. D., Mathieu, M., O'Leary, B. F., Sperone, F. G., Straughen, J. K., & Reiners, J. J. J. (2020). Prenatal airshed pollutants and preterm birth in an observational birth cohort study in Detroit, Michigan, USA. Environ Res, 189, 109845.

Chambers, D.M., McElprang, D.O., Waterhouse, M.G., and Blount, B.C. (2006). An improved approach for accurate quantitation of benzene, toluene, ethylbenzene, xylene, and styrene in blood. Anal Chem 78, 5375-383.

Chan, P.C., Hasemani, J.K., Mahleri, J., and Aranyi, C. (1998). Tumor induction in F344/N rats and B6C3F1 mice following inhalation exposure to ethylbenzene. Toxicol Lett 99, 23-32.

Ethylbenzene References Reviewed
Supplemental Material A

Chang M, Lee D, Park H, Ha M, Hong YC, Kim Y, Kim BN, Kim Y, Lim YH, Ha EH. 2018. Prenatal TVOCs exposure negatively influences postnatal neurobehavioral development. Sci Total Environ 618: 977–81.

Chang M, Park H, Ha M, Hong YC, Lim YH, Kim Y, Kim YJ, Lee D, Ha EH. 2017. The effect of prenatal TVOC exposure on birth and infantile weight: the Mothers and Children's Environmental Health study. Pediatr Res 82: 423–8.

Chen X, Feng L, Luo H, Cheng H. 2016. Health risk equations and risk assessment of airborne benzene homologues exposure to drivers and passengers in taxi cabins. Environ Sci Pollut Res Int 23: 4797–811.

Chen, C.S., Hseu, Y.C., Liang, S.H., Kuo, J.Y., and Chen, S.C. (2008). Assessment of genotoxicity of methyl-tert-butyl ether, benzene, toluene, ethylbenzene, and xylene to human lymphocytes using comet assay. J Hazard Mater 153, 351-56.

Cragg, S.T., Clarke, E.A., Daly, I.W., Miller, R.R., Terrill, J.B., and Ouellette, R.E. (1989). Subchronic inhalation toxicity of ethylbenzene in mice, rats, and rabbits. Fundam Appl Toxicol 13, 399-408.

Cruz SL, Gauthereau-Torres MY, Rivera-Garcia MT. 2016. Structure-activity relationship for the anticonvulsant effects of organic solvents. Neurotoxicology 57: 121–7.

Cushing, L. J., Vavra-Musser, K., Chau, K., Franklin, M., & Johnston, J. E. (2020). Flaring from Unconventional Oil and Gas Development and Birth Outcomes in the Eagle Ford Shale in South Texas. Environ Health Perspect, 128(7), 77003.

da Silva, M.L., and Alvarez, P.J. (2010). Indole-based assay to assess the effect of ethanol on Pseudomonas putida F1 dioxygenase activity. Biodegradation 21, 425-430.

Dai H, Jing S, Wang H, Ma Y, Li L, Song W, Kan H. 2017. VOC characteristics and inhalation health risks in newly renovated residences in Shanghai, China. Sci Total Environ 577: 73–83.

Davidson, C. J., Hannigan, J. H., & Bowen, S. E. (2021). Effects of inhaled combined Benzene, Toluene, Ethylbenzene, and Xylenes (BTEX): Toward an environmental exposure model. Environ Toxicol Pharmacol, 81, 103518.

De Celis, R., Feria-Velasco, A., Gonzalez-Unzaga, M., Torres-Calleja, J., and Pedron-Nuevo, N. (2000). Semen quality of workers occupationally exposed to hydrocarbons. Fertil Steril 73, 221-28.

Dehghani F, Omidi F, Heravizadeh O, Barati Chamgordani S, Gharibi V, Sotoudeh Manesh A. 2018. Occupational health risk assessment of volatile organic compounds emitted from the coke production unit of a steel plant. Int J Occup Saf Ergon 1–6.

Dehghani M, Fazlzadeh M, Sorooshian A, Tabatabaee HR, Miri M, Baghani AN, Delikhoon M, Mahvi AH, Rashidi M. 2018. Characteristics and health effects of BTEX in a hot spot for urban pollution. Ecotoxicol Environ Saf 155: 133–43.

Dellefratte, K., Stingone, J. A., & Claudio, L. (2019). Combined association of BTEX and material hardship on ADHD-suggestive behaviours among a nationally representative sample of US children. Paediatr Perinat Epidemiol, 33(6), 482-489.

Ding, N., Batterman, S., & Park, S. K. (2020). Exposure to Volatile Organic Compounds and Use of Feminine Hygiene Products Among Reproductive-Aged Women in the United States. J Womens Health (Larchmt), 29(1), 65-73.

Doherty BT, Kwok RK, Curry MD, Ekenga C, Chambers D, Sandler DP, Engel LS. 2017. Associations between blood BTEXS concentrations and hematologic parameters among adult residents of the U.S. Gulf States. Environ Res 156: 579–87.

Du Z, Mo J, Zhang Y. 2014. Risk assessment of population inhalation exposure to volatile organic compounds and carbonyls in urban China. Environ Int 73: 33–45.

El-Metwally D, Chain K, Stefanak MP, Alwis U, Blount BC, LaKind JS, Bearer CF. 2018. Urinary metabolites of volatile organic compounds of infants in the neonatal intensive care unit. Pediatr Res

Elliott EG, Ettinger AS, Leaderer BP, Bracken MB, Deziel NC. 2017. A systematic evaluation of chemicals in hydraulic-fracturing fluids and wastewater for reproductive and developmental toxicity. J Expo Sci Environ Epidemiol 27: 90–9.

Elliott EG, Trinh P, Ma X, Leaderer BP, Ward MH, Deziel NC. 2017. Unconventional oil and gas development and risk of childhood leukemia: Assessing the evidence. Sci Total Environ 576: 138–47.

Engelhardt, G. (2006). In vivo micronucleus test in mice with 1-phenylethanol. Arch Toxicol 80, 868-872.

Ethylbenzene. IARC Monogr Eval Carcinog Risks Hum. 2000, 77, 227-266.

Faber, W.D., Roberts, L.S., Stump, D.G., Beck, M., Kirkpatrick, D., Regan, K.S., Tort, M., Moran, E., and Banton, M. (2007). Inhalation developmental neurotoxicity study of ethylbenzene in Crl-CD rats. Birth Defects Res B Dev Reprod Toxicol 80, 34-48.

Faber, W.D., Roberts, L.S., Stump, D.G., Tardif, R., Krishnan, K., Tort, M., Dimond, S., Dutton, D., Moran, E., and Lawrence, W. (2006). Two generation reproduction study of ethylbenzene by inhalation in Crl-CD rats.Birth Defects Res B Dev Reprod Toxicol 77, 10-21.

Fabian E, Bordag N, Herold M, Kamp H, Krennrich G, Looser R, Ma-Hock L, Mellert W, Montoya G, Peter E, Prokudin A, Spitzer M, Strauss V, Walk T, Zbranek R, van Ravenzwaay B. 2016. Metabolite profiles of rats in repeated dose toxicological studies after oral and inhalative exposure. Toxicol Lett 255: 11–23.

Francioni, E., Fillmann, G., Hamacher, C., Wagener, A.d.e. .L., Depledge, M.H., Readman, J.W., and Meniconi, M.d.e. .F. (2003). Evaluation of a commercially available ELISA kit as a tool to determine BTEX in groundwater. Environ Technol 24, 665-670.

Franck U, Weller A, Roder SW, Herberth G, Junge KM, Kohajda T, von Bergen M, Rolle-Kampczyk U, Diez U, Borte M, Lehmann I. 2014. Prenatal VOC exposure and redecoration are related to wheezing in early infancy. Environ Int 73: 393–401.

Fustinoni, S., Giampiccolo, R., Pulvirenti, S., Buratti, M., and Colombi, A. (1999). Headspace solid-phase microextraction for the determination of benzene, toluene, ethylbenzene and xylenes in urine. J Chromatogr B Biomed Sci Appl 723, 105-115.

Gherardi, M., Gordiani, A., and Gatto, M. (2010). Development and validation of method for analysis of some ototoxic solvents in saliva matrix by headspace gas chromatography/mass spectrometry. J Chromatogr B Analyt Technol Biomed Life Sci 878, 2391-96.

Ghosh JK, Wilhelm M, Su J, Goldberg D, Cockburn M, Jerrett M, Ritz B. 2012. Assessing the influence of traffic-related air pollution on risk of term low birth weight on the basis of land-use-based regression models and measures of air toxics. Am J Epidemiol 175: 1262–74.

Gill, R., Hatchett, S.E., Osselton, M.D., Wilson, H.K., and Ramsey, J.D. (1988). Sample handling and storage for the quantitative analysis of volatile compounds in blood: the determination of toluene by headspace gas chromatography. J Anal Toxicol 12, 141-46.

Gong, X., Lin, Y., Bell, M. L., & Zhan, F. B. (2018). Associations between maternal residential proximity to air emissions from industrial facilities and low birth weight in Texas, USA. Environ Int, 120, 181-198.

Gong, X., Huang, Y., Duong, J., Leng, S., Zhan, F. B., Guo, Y., Lin, Y., & Luo, L. (2023). Industrial air pollution and low birth weight in New Mexico, USA. J Environ Manage, 348, 119236. https://doi.org/10.1016/j.jenvman.2023.119236

Gonzalez JL, Pell A, Lopez-Mesas M, Valiente M. 2017. Simultaneous determination of BTEX and their metabolites using solid-phase microextraction followed by HPLC or GC/MS: An application in teeth as environmental biomarkers. Sci Total Environ 603-604: 109–17.

Groth C, Banerjee S, Ramachandran G, Stenzel MR, Sandler DP, Blair A, Engel LS, Kwok RK, Stewart PA. 2017. Bivariate Left-Censored Bayesian Model for Predicting Exposure: Preliminary Analysis of Worker Exposure during the Deepwater Horizon Oil Spill. Ann Work Expo Health 61: 76–86.

Gunsch, C.K., Kinney, K.A., Szaniszlo, P.J., and Whitman, C.P. (2006). Quantification of homogentisate-1,2-dioxygenase expression in a fungus degrading ethylbenzene. J Microbiol Methods 67, 257-265.

Haigler, B.E., and Spain, J.C. (1989). Degradation of p-chlorotoluene by a mutant of Pseudomonas sp. strain JS6. Appl Environ Microbiol 55, 372-79.

Hardin BD, Bond GP, Sikov MR, Andrew FD, Beliles RP, Niemeier RW. 1981. Testing of selected workplace chemicals for teratogenic potential. Scand J Work Environ Health 7 Suppl 4: 66–75.

Harrath, A. H., Alrezaki, A., Jalouli, M., Aldawood, N., Aldahmash, W., Mansour, L., & Alwasel, S. (2022). Ethylbenzene exposure disrupts ovarian function in Wistar rats via altering folliculogenesis and steroidogenesis-related markers and activating autophagy and apoptosis. Ecotoxicol Environ Saf, 229, 113081.

HEI-Energy Research Committee. Potential human health effects associated with unconventional Oil and gas development: a systematic review of the epidemiology literature. Special Report 1. https://www.heienergy.org/system/files/hei-energy-epi-lit-review.pdf

Heibati B, Godri Pollitt KJ, Charati JY, Ducatman A, Shokrzadeh M, Karimi A, Mohammadyan M. 2018. Biomonitoring-based exposure assessment of benzene, toluene, ethylbenzene and xylene among workers at petroleum distribution facilities. Ecotoxicol Environ Saf 149: 19–25.

Heibati B, Pollitt KJG, Karimi A, Yazdani Charati J, Ducatman A, Shokrzadeh M, Mohammadyan M. 2017. BTEX exposure assessment and quantitative risk assessment among petroleum product distributors. Ecotoxicol Environ Saf 144: 445–9.

Henderson, L., Brusick, D., Ratpan, F., and Veenstra, G. (2007). A review of the genotoxicity of ethylbenzene. Mutat Res 635, 81-89.

Hill, M., Stabile, C., Steffen, L.K., and Hill, A. (2002). Toxic effects of endocrine disrupters on freshwater sponges: common developmental abnormalities. Environ Pollut 117, 295-300.

Hong JY, Yu SY, Kim GW, Ahn JJ, Kim Y, Lim S, Son SW, Hwang SY. 2016. Identification of time-dependent biomarkers and effects of exposure to volatile organic compounds using high-throughput analysis. Environ Toxicol 31: 1563–70.

Hongyan, L., Zexiong, Z., Shiwei, X., He, X., Yinian, Z., Haiyun, L., & Zhongsheng, Y. (2019). Study on transformation and degradation of bisphenol A by Trametes versicolor laccase and simulation of molecular docking. Chemosphere, 224, 743-750.

Huff, J. (2002). Chemicals studied and evaluated in long-term carcinogenesis bioassays by both the Ramazzini Foundation and the National Toxicology Program: in tribute to Cesare Maltoni and David Rall. Ann N Y Acad Sci 982, 208-230.

Inayat-Hussain SH, Fukumura M, Muiz Aziz A, Jin CM, Jin LW, Garcia-Milian R, Vasiliou V, Deziel NC. 2018. Prioritization of reproductive toxicants in unconventional oil and gas operations using a multi-country regulatory data-driven hazard assessment. Environ Int 117: 348–58.

Jain RB. 2015. Levels of selected urinary metabolites of volatile organic compounds among children aged 6-11 years. Environ Res 142: 461–70.

Janitz, A. E., Dao, H. D., Campbell, J. E., Stoner, J. A., & Peck, J. D. (2019). The association between natural gas well activity and specific congenital anomalies in Oklahoma, 1997-2009. Environ Int, 122, 381-388.

Jephcote, C., Brown, D., Verbeek, T., & Mah, A. (2020). A systematic review and meta-analysis of haematological malignancies in residents living near petrochemical facilities. Environ Health, 19(1), 53.

Kasemy, Z. A., Kamel, G. M., Abdel-Rasoul, G. M., & Ismail, A. A. (2019). Environmental and Health Effects of Benzene Exposure among Egyptian Taxi Drivers. J Environ Public Health, 2019, 7078024.

Kassotis CD, Klemp KC, Vu DC, Lin CH, Meng CX, Besch-Williford CL, Pinatti L, Zoeller RT, Drobnis EZ, Balise VD, Isiguzo CJ, Williams MA, Tillitt DE, Nagel SC. 2015. Endocrine-Disrupting Activity of Hydraulic Fracturing Chemicals and Adverse Health Outcomes After Prenatal Exposure in Male Mice. Endocrinology 156: 4458–73.

Kassotis CD, Tillitt DE, Lin CH, McElroy JA, Nagel SC. 2016. Endocrine-Disrupting Chemicals and Oil and Natural Gas Operations: Potential Environmental Contamination and Recommendations to Assess Complex Environmental Mixtures. Environ Health Perspect 124: 256–64.

Kassotis CD, Vu DC, Vo PH, Lin CH, Cornelius-Green JN, Patton S, Nagel SC. 2018. Endocrine-Disrupting Activities and Organic Contaminants Associated with Oil and Gas Operations in Wyoming Groundwater. Arch Environ Contam Toxicol

Kim, B.M., Park, E.k., LeeAn, S.Y., Ha, M., Kim, E.J., Kwon, H., Hong, Y.C., Jeong, W.C., Hur, J., et al. (2009). [BTEX exposure and its health effects in pregnant women following the Hebei Spirit oil spill]. J Prev Med Public Health 42, 96-103.

Kim, J.H., Moon, J.Y., Park, E.-Y., Lee, K.-H., and Hong, Y.-C. (2011). Changes in Oxidative Stress Biomarker and Gene Expression Levels in Workers Exposed to Volatile Organic Compounds. Ind Health 49, 8-14.

Kim, M.N., Park, H.H., Lim, W.K., and Shin, H.J. (2005). Construction and comparison of Escherichia coli whole-cell biosensors capable of detecting aromatic compounds. J Microbiol Methods 60, 235-245.

Kljaković-Gašpić, Z., Herceg Romanić, S., Bituh, T., Kašuba, V., Brčić Karačonji, I., Brajenović, N., Franulović, I., Jurasović, J., Klinčić, D., Kopjar, N., Marović, G., Milić, M., Orct, T., Sekovanić, A., & Želježić, D. (2018). Assessment of multiple anthropogenic contaminants and their potential genotoxicity in the aquatic environment of Plitvice Lakes National Park, Croatia. Environ Monit Assess, 190(11), 694.

Kuster, M., Díaz-Cruz, S., Rosell, M., López de Alda, M., and Barceló, D. (2010). Fate of selected pesticides, estrogens, progestogens and volatile organic compounds during artificial aquifer recharge using surface waters. Chemosphere 79, 880-86.

Lee, E.G., Slaven, J., Bowen, R.B., and Harper, M. (2011). Evaluation of the COSHH Essentials model with a mixture of organic chemicals at a medium-sized paint producer. Ann Occup Hyg 55, 16-29.

Lei, T., Qian, H., Yang, J., & Hu, Y. (2023). The association analysis between exposure to volatile organic chemicals and obesity in the general USA population: A cross-sectional study from NHANES program. Chemosphere, 315, 137738. https://doi.org/10.1016/j.chemosphere.2023.137738

Li J, Lu S, Liu G, Zhou Y, Lv Y, She J, Fan R. 2015. Co-exposure to polycyclic aromatic hydrocarbons, benzene and toluene and their dose-effects on oxidative stress damage in kindergarten-aged children in Guangzhou, China. Sci Total Environ 524-525: 74–80.

Li, A.A., Maurissen, J.P., Barnett, J.F., Foss, J., Freshwater, L., Garman, R.H., Peachee, V.L., Hong, S.J., Stump, D.G., and Bus, J.S. (2010). Oral gavage subchronic neurotoxicity and inhalation subchronic immunotoxicity studies of ethylbenzene in the rat. Neurotoxicology 31, 247-258.

Lim SK, Shin HS, Yoon KS, Kwack SJ, Um YM, Hyeon JH, Kwak HM, Kim JY, Kim TY, Kim YJ, Roh TH, Lim DS, Shin MK, Choi SM, Kim HS, Lee BM. 2014. Risk assessment of volatile organic compounds benzene, toluene, ethylbenzene, and xylene (BTEX) in consumer products. J Toxicol Environ Health A 77: 1502–21.

Liu B, Jia C. 2015. Effects of exposure to mixed volatile organic compounds on the neurobehavioral test performance in a cross-sectional study of US adults. Int J Environ Health Res 25: 349–63.

Liu FF, Escher BI, Were S, Duffy L, Ng JC. 2014. Mixture effects of benzene, toluene, ethylbenzene, and xylenes (BTEX) on lung carcinoma cells via a hanging drop air exposure system. Chem Res Toxicol 27: 952–9.

Liu FF, Peng C, Ng JC. 2015. BTEX in vitro exposure tool using human lung cells: trips and gains. Chemosphere 128: 321–6.

Liu Y, Li H, Fu X, Guo H, Meng R, Lu W, Zhao M, Wang H. 2016. Health risk impacts analysis of fugitive aromatic compounds emissions from the working face of a municipal solid waste landfill in China. Environ Int 97: 15–27.

Liu, K. P., Su, Y. W., Zhang, J. W., Wang, Z., Ma, Y. Y., Liu, Y. M., & Xiao, Y. M. (2021). [The effects of ethylbenzene on HEI-OC1 cells proliferation and oxidative stress level]. Zhonghua Lao Dong Wei Sheng Zhi Ye Bing Za Zhi, 39(1), 44-47.

Liu, X.T., Yang, D.Y., Wang, Y.R., Wang, Q., Kuang, D., Zhang, M., Qiao, L.J., Li, J.G., Yang, X.Y., and Zhao, S.L. (2013). [Influence of ethylbenzene on oxidative damage and apoptosis in rat renal epithelial cells NRK-52e]. Zhonghua Lao Dong Wei Sheng Zhi Ye Bing Za Zhi 31, 133-36.

Liu, Y., Zhou, Q., Xie, X., Lin, D., and Dong, L. (2010). Oxidative stress and DNA damage in the earthworm Eisenia fetida induced by toluene, ethylbenzene and xylene. Ecotoxicology 19, 1551-59.

López-Vargas, R., Méndez-Serrano, A., Albores-Medina, A., Oropeza-Hernández, F., Hernández-Cadena, L., Mercado-Calderón, F., Alvarado-Toledo, E., Herrera-Morales, S., Arellano-Aguilar, O., García-Vargas, G., & Montero-Montoya, R. (2018). Oxidative stress index is increased in children exposed to industrial discharges and is inversely correlated with metabolite excretion of voc. Environ Mol Mutagen, 59(7), 639-652.

Lu, F., Li, S., Shen, B., Zhang, J., Liu, L., Shen, X., & Zhao, R. (2020). The emission characteristic of VOCs and the toxicity of BTEX from different mosquito-repellent incenses. J Hazard Mater, 384, 121428.

Madaniyazi, L., Jung, C. R., Fook Sheng Ng, C., Seposo, X., Hashizume, M., & Nakayama, S. F. (2022). Early life exposure to indoor air pollutants and the risk of neurodevelopmental delays: The Japan Environment and Children's Study. Environ Int, 158, 107004.

Maiolini, E., Knopp, D., Niessner, R., Eremin, S., Bolelli, L., Ferri, E.N., and Girotti, S. (2010). Chemiluminescent ELISA for the BTEX determination in water and soil. Anal Sci 26, 773-77.

Mannisto T, Mendola P, Laughon Grantz K, Leishear K, Sundaram R, Sherman S, Ying Q, Liu D. 2015. Acute and recent air pollution exposure and cardiovascular events at labour and delivery. Heart 101: 1491–8.

Marchand A, Aranda-Rodriguez R, Tardif R, Nong A, Haddad S. 2015. Human inhalation exposures to toluene, ethylbenzene, and m-xylene and physiologically based pharmacokinetic modeling of exposure biomarkers in exhaled air, blood, and urine. Toxicol Sci 144: 414–24.

Marchand A, Aranda-Rodriguez R, Tardif R, Nong A, Haddad S. 2016. Evaluation and modeling of the impact of coexposures to VOC mixtures on urinary biomarkers. Inhal Toxicol 28: 260–73.

Martínez, C., Ramírez, N., Gómez, V., Pocurull, E., and Borrull, F. (2013). Simultaneous determination of 76 micropollutants in water samples by headspace solid phase microextraction and gas chromatography-mass spectrometry. Talanta 116, 937-945.

Martins EM, Borba PF, Dos Santos NE, Dos Reis PT, Silveira RS, Correa SM. 2016. The relationship between solvent use and BTEX concentrations in occupational environments. Environ Monit Assess 188: 608.

Mazzeo, D.E., Matsumoto, S.T., Levy, C.E., de Angelis, D.d.e. .F., and Marin-Morales, M.A. (2013). Application of micronucleus test and comet assay to evaluate BTEX biodegradation. Chemosphere 90, 1030-36.

Mellert, W., Deckardt, K., Kaufmann, W., & van Ravenzwaay, B. (2007). Ethylbenzene: 4-and 13-week rat oral toxicity. Archives of Toxicology, 81(5), 361-370.

Mendola P, Wallace M, Liu D, Robledo C, Mnnist T, Grantz KL. 2016. Air pollution exposure and preeclampsia among US women with and without asthma. Environ Res 148: 248–55.

Meyer-Monath M, Beaumont J, Morel I, Rouget F, Tack K, Lestremau F. 2014. Analysis of BTEX and chlorinated solvents in meconium by headspace-solid-phase microextraction gas chromatography coupled with mass spectrometry. Anal Bioanal Chem 406: 4481–90.

Meyer-Monath M, Chatellier C, Rouget F, Morel I, Lestremau F. 2014. Development of a multi-residue method in a fetal matrix: analysis of meconium. Anal Bioanal Chem 406: 7785–97.

Miri M, Rostami Aghdam Shendi M, Ghaffari HR, Ebrahimi Aval H, Ahmadi E, Taban E, Gholizadeh A, Yazdani Aval M, Mohammadi A, Azari A. 2016. Investigation of outdoor BTEX: Concentration, variations, sources, spatial distribution, and risk assessment. Chemosphere 163: 601–9.

Montero-Montoya, R., López-Vargas, R., & Arellano-Aguilar, O. (2018). Volatile Organic Compounds in Air: Sources, Distribution, Exposure and Associated Illnesses in Children. Ann Glob Health, 84(2), 225-238.

Moolla R, Curtis CJ, Knight J. 2015. Occupational exposure of diesel station workers to BTEX compounds at a bus depot. Int J Environ Res Public Health 12: 4101–15.

Mueller, S., Dennison, G., & Liu, S. (2021). An Assessment on Ethanol-Blended Gasoline/Diesel Fuels on Cancer Risk and Mortality. Int J Environ Res Public Health, 18(13).

Mullen, K. R., Rivera, B. N., Tidwell, L. G., Ivanek, R., Anderson, K. A., & Ainsworth, D. M. (2020). Environmental surveillance and adverse neonatal health outcomes in foals born near unconventional natural gas development activity. Sci Total Environ, 731, 138497.

Nakhjirgan, P., Kashani, H., Naddafi, K., Nabizadeh, R., Amini, H., & Yunesian, M. (2019). Maternal exposure to air pollutants and birth weight in Tehran, Iran. J Environ Health Sci Eng, 17(2), 711-717.

National Toxicology Program (1999). NTP Toxicology and Carcinogenesis Studies of Ethylbenzene (CAS No. 100-41-4) in F344/N Rats and B6C3F1 Mice (Inhalation Studies). Natl Toxicol Program Tech Rep Ser 466, 1-231.

Neghab, M., Nourozi, M. A., Shahtaheri, S. J., Mansoori, Y., Bazzaz, J. T., & Nedjat, S. (2018). Effects of Genetic Polymorphism on Susceptibility to Nephrotoxic Properties of BTEXs Compounds. J Occup Environ Med, 60(8), e377-e382.

Nicole, W. (2020). On Wells and Wellness: Oil and Gas Flaring as a Potential Risk Factor for Preterm Birth. Environ Health Perspect, 128(11), 114004. https://doi.org/10.1289/EHP7952

Niederlehner, B.R., Cairns, J., and Smith, E.P. (1998). Modeling acute and chronic toxicity of nonpolar narcotic chemicals and mixtures to Ceriodaphnia dubia. Ecotoxicol Environ Saf 39, 136-146.

North MA, Rodriguez-Estival J, Smits JEG. 2017. Biomarker Sensitivity to Vehicle Exhaust in Experimentally Exposed European Starlings. Environ Sci Technol 51: 13427–35.

OECD SIDS Ethylbenzene - SIDS Initial Assessment Report for SIAM 14. March 2002.

Okamoto, Y., Hayashi, T., Matsunami, S., Ueda, K., Toda, C., Kawanishi, S., and Kojima, N. (2006). Formation of DNA damaging product from light-irradiated nonylphenol. JOURNAL OF HEALTH SCIENCE 52, 91-95.

Onink, F., Meindersma, W., Burghoff, B., Weggemans, W., Aerts, G., and de Haan, A. (2014). Ion Chromatography as a Novel Method to Quantify the Solubility of Pyridinium Ionic Liquids in Organic Solvents. J Chromatogr Sci

Paciência, I., Cavaleiro Rufo, J., Silva, D., Martins, C., Mendes, F., Farraia, M., Delgado, L., de Oliveira Fernandes, E., Padrão, P., Moreira, P., Severo, M., Barros, H., & Moreira, A. (2019).

Exposure to indoor endocrine-disrupting chemicals and childhood asthma and obesity. Allergy, 74(7), 1277-1291.

Paez-Martinez, N., Lopez-Rubalcava, C., and Cruz, S.L. (2003). Basic research advances on the in vivo effects of abused solvents. SALUD MENTAL 26, 8-16.

Pankiewicz-Sperka M, Stanczyk K, Plaza GA, Kwasniewska J, Nalecz-Jawecki G. 2014. Assessment of the chemical, microbiological and toxicological aspects of post-processing water from underground coal gasification. Ecotoxicol Environ Saf 108: 294–301.

Peng C, Lee JW, Sichani HT, Ng JC. 2015. Toxic effects of individual and combined effects of BTEX on Euglena gracilis. J Hazard Mater 284: 10–8.

Philibert DA, Philibert CP, Lewis C, Tierney KB. 2016. Comparison of Diluted Bitumen (Dilbit) and Conventional Crude Oil Toxicity to Developing Zebrafish. Environ Sci Technol 50: 6091–8.

Phoenix, P., Keane, A., Patel, A., Bergeron, H., Ghoshal, S., and Lau, P.C. (2003). Characterization of a new solvent-responsive gene locus in Pseudomonas putida F1 and its functionalization as a versatile biosensor. Environ Microbiol 5, 1309-327.

Price, K., and Krishnan, K. (2011). An integrated QSAR-PBPK modelling approach for predicting the inhalation toxicokinetics of mixtures of volatile organic chemicals in the rat. SAR QSAR Environ Res 22, 107-128.

Quiros-Alcala L, Wilson S, Witherspoon N, Murray R, Perodin J, Trousdale K, Raspanti G, Sapkota A. 2016. Volatile organic compounds and particulate matter in child care facilities in the District of Columbia: Results from a pilot study. Environ Res 146: 116–24.

Rafiee, A., Delgado-Saborit, J. M., Sly, P. D., Amiri, H., & Hoseini, M. (2022). Exploring urinary biomarkers to assess oxidative DNA damage resulting from BTEX exposure in street children. Environ Res, 203, 111725.

Rajendran, R., Ragavan, R. P., Al-Sehemi, A. G., Uddin, M. S., Aleya, L., & Mathew, B. (2022). Current understandings and perspectives of petroleum hydrocarbons in Alzheimer’s disease and Parkinson’s disease: a global concern. Environ Sci Pollut Res Int, 29(8), 10928-10949.

Ramakrishnan, A., Lupo, P.J., Agopian, A.J., Linder, S.H., Stock, T.H., Langlois, P.H., and Craft, E. (2013). Evaluating the effects of maternal exposure to benzene, toluene, ethyl benzene, and xylene on oral clefts among offspring in Texas: 1999-2008. Birth Defects Res A Clin Mol Teratol 97, 532-37.

Ran J, Qiu H, Sun S, Tian L. 2018. Short-term effects of ambient benzene and TEX (toluene, ethylbenzene, and xylene combined) on cardiorespiratory mortality in Hong Kong. Environ Int 117: 91–8.

Reutman, S.R., LeMasters, G.K., Knecht, E.A., Shukla, R., Lockey, J.E., Burroughs, G.E., and Kesner, J.S. (2002). Evidence of reproductive endocrine effects in women with occupational fuel and solvent exposures. Environ Health Perspect 110, 805-811.

Riedel TP, DeMarini DM, Zavala J, Warren SH, Corse EW, Offenberg JH, Kleindienst TE, Lewandowski M. 2018. Mutagenic atmospheres resulting from the photooxidation of aromatic hydrocarbon and NOx mixtures. Atmos Environ (1994) 178: 164–72.

Robrock, K.R., Mohn, W.W., Eltis, L.D., and Alvarez-Cohen, L. (2011). Biphenyl and ethylbenzene dioxygenases of Rhodococcus jostii RHA1 transform PBDEs. Biotechnol Bioeng 108, 313-321.

Rouget, F., Bihannic, A., Cordier, S., Multigner, L., Meyer-Monath, M., Mercier, F., Pladys, P., & Garlantezec, R. (2021). Petroleum and Chlorinated Solvents in Meconium and the Risk of Hypospadias: A Pilot Study. Front Pediatr, 9, 640064.

Ruiz, P., Emond, C., McLanahan, E. D., Joshi-Barr, S., & Mumtaz, M. (2020). Exploring Mechanistic Toxicity of Mixtures Using PBPK Modeling and Computational Systems Biology. Toxicol Sci, 174(1), 38-50.

Saalberg Y, Wolff M. 2016. VOC breath biomarkers in lung cancer. Clin Chim Acta 459: 5–9.

Saillenfait, A.-M., Gallissot, F., Sabate, J.-P., Bourges-Abella, N., and Muller, S. (2007). Developmental toxic effects of ethylbenzene or toluene alone and in combination with butyl acetate in rats after inhalation exposure. J Appl Toxicol 27, 32-42.

Saillenfait, A.M., Gallissot, F., Morel, G., and Bonnet, P. (2003). Developmental toxicities of ethylbenzene, ortho-, meta-, para-xylene and technical xylene in rats following inhalation exposure. Food Chem Toxicol 41, 415-429.

Saillenfait, A.M., Gallissot, F., Sabaté, J.P., Bourges-Abella, N., Cadot, R., Morel, G., and Lambert, A.M. (2006). Developmental toxicity of combined ethylbenzene and methylethylketone administered by inhalation to rats. Food Chem Toxicol 44, 1287-298.

Sammarco PW, Kolian SR, Warby RA, Bouldin JL, Subra WA, Porter SA. 2016. Concentrations in human blood of petroleum hydrocarbons associated with the BP/Deepwater Horizon oil spill, Gulf of Mexico. Arch Toxicol 90: 829–37.

Sapouckey SA, Kassotis CD, Nagel SC, Vandenberg LN. 2018. Prenatal Exposure to Unconventional Oil and Gas Operation Chemical Mixtures Altered Mammary Gland Development in Adult Fetonie Mice. Endocrinology 159: 1277–89.

Serrano-Lomelin, J., Nielsen, C. C., Jabbar, M. S. M., Wine, O., Bellinger, C., Villeneuve, P. J., Stieb, D., Aelicks, N., Aziz, K., Buka, I., Chandra, S., Crawford, S., Demers, P., Erickson, A. C., Hystad, P., Kumar, M., Phipps, E., Shah, P. S., Yuan, Y., . . . Osornio-Vargas, A. R. (2019). Interdisciplinary-driven hypotheses on spatial associations of mixtures of industrial air pollutants with adverse birth outcomes. Environ Int, 131, 104972.

Silvestre, R. T., Bravo, M., Santiago, F., Delmonico, L., Scherrer, L., Otero, U. B., Liehr, T., Alves, G., Chantre-Justino, M., & Ornellas, M. H. (2020). Hypermethylation in Gene Promoters Are Induced by Chronic Exposure to Benzene, Toluene, Ethylbenzene and Xylenes. Pak J Biol Sci, 23(4), 518-525.

Sirotkin AV, Harrath AH. 2017. Influence of oil-related environmental pollutants on female reproduction. Reprod Toxicol 71: 142–5.

Sisto, R., Cavallo, D., Ursini, C. L., Fresegna, A. M., Ciervo, A., Maiello, R., Paci, E., Pigini, D., Gherardi, M., Gordiani, A., L'Episcopo, N., Tranfo, G., Capone, P., Carbonari, D., Balzani, B., & Chiarella, P. (2020). Direct and Oxidative DNA Damage in a Group of Painters Exposed to VOCs: Dose - Response Relationship. Front Public Health, 8, 445.

Skurský, L., Khan, A.N., Saleem, M.N., and al-Tamer, Y.Y. (1992). A new potent inhibitor of horse liver alcohol dehydrogenase: p-methylbenzyl hydroperoxide. Biochem Int 26, 899-904.

Song MK, Ryu JC. 2015. Blood miRNAs as sensitive and specific biological indicators of environmental and occupational exposure to volatile organic compound (VOC). Int J Hyg Environ Health 218: 590–602.

Spinder, N., Prins, J. R., Bergman, J. E. H., Smidt, N., Kromhout, H., Boezen, H. M., & de Walle, H. E. K. (2019). Congenital anomalies in the offspring of occupationally exposed mothers: a systematic review and meta-analysis of studies using expert assessment for occupational exposures. Hum Reprod, 34(5), 903-919.

Sreng L, Temime-Roussel B, Wortham H, Mourre C. 2017. Chemical Identification of “Maternal Signature Odors” in Rat. Chem Senses 42: 211–22.

Staudt, A. M., Whitworth, K. W., Chien, L. C., Whitehead, L. W., & Gimeno Ruiz de Porras, D. (2019). Association of organic solvents and occupational noise on hearing loss and tinnitus among adults in the U.S., 1999-2004. Int Arch Occup Environ Health, 92(3), 403-413.

Stephens, S.M., Alkindi, A.Y.A., Waring, C.P., and Brown, J.A. (1997). Corticosteroid and thyroid responses of larval and juvenile turbot exposed to the water-soluble fraction of crude oil. JOURNAL OF FISH BIOLOGY 50, 953-964.

Stingone JA, McVeigh KH, Claudio L. 2017. Early-life exposure to air pollution and greater use of academic support services in childhood: a population-based cohort study of urban children. Environ Health 16: 2.

Stott WT, Day SJ, McGuirk RJ, Johnson KA. 2001. Ethylbenzene: Four-Week Mechanism of Tumorigenicity Study in Fischer 344 Rats and B6C3F1 Mice. Report of Toxicology & Environmental Research and Consulting The Dow Chemical Company, Midland, Michigan .

Stott WT, Johnson KA, Bahnemann R, Day SJ, McGuirk RJ. 2003. Evaluation of potential modes of action of inhaled ethylbenzene in rats and mice. Toxicol Sci 71: 53–66.

Stott WT, Johnson KA, Day SJ, McGuirk RJ. 1999. Ethylbenzene: Mechanism of tumorigenicity in Fischer 344 rats and B6C3F1 mice. Report of Toxicology & Environmental Research and Consulting The Dow Chemical Company, Midland, Michigan

Sweeney LM, Kester JE, Kirman CR, Gentry PR, Banton MI, Bus JS, Gargas ML. 2015. Risk assessments for chronic exposure of children and prospective parents to ethylbenzene (CAS No. 100-41-4). Crit Rev Toxicol 45: 662–726.

Szaleniec, M., Witko, M., Tadeusiewicz, R., and Goclon, J. (2006). Application of artificial neural networks and DFT-based parameters for prediction of reaction kinetics of ethylbenzene dehydrogenase. J Comput Aided Mol Des 20, 145-157.

Takagi, S., Sato, Y., Kokubun, A., Inomata, E., & Agatsuma, Y. (2020). Odor-active compounds from the gonads of Mesocentrotus nudus sea urchins fed Saccharina japonica kelp. PLoS One, 15(4), e0231673.

Take, M., Takeda, T., Ishikawa, H., Matsumoto, M., Nagano, K., & Fukushima, S. (2020). Area under the blood concentration-time curve (AUC) of ethylbenzene concentration in rats: relationship to inhalation and oral administration route-dose. J Environ Sci Health A Tox Hazard Subst Environ Eng, 55(14), 1596-1603.

Takeda, H., Yamada, A., Miyauchi, K., Masai, E., and Fukuda, M. (2004). Characterization of transcriptional regulatory genes for biphenyl degradation in Rhodococcus sp. strain RHA1. J Bacteriol 186, 2134-146.

Tallandier, V., Chalansonnet, M., Merlen, L., Boucard, S., Thomas, A., Campo, P., & Pouyatos, B. (2021). An in vitro model to assess the peripheral vestibulotoxicity of aromatic solvents. Neurotoxicology, 84, 105-113.

The Committee for Recommendation of Occupational Exposure Limits, Japan Society for Occupational Health. (2020). Occupational exposure limits for ethyl benzene, dimethyl terephthalate and hydrogen fluoride, and carcinogenicity and reproductive toxicant classifications. J Occup Health, 62(1), e12151.

The Committee for Recommendation of Occupational Exposure Limits, Japan Society for Occupational Health. (2021). Occupational exposure limits for acetaldehyde, 2-bromopropane, glyphosate, manganese and inorganic manganese compounds, and zinc oxide nanoparticle, and the biological exposure indices for cadmium and cadmium compounds and ethylbenzene, and carcinogenicity, occupational sensitizer, and reproductive toxicant classifications. J Occup Health, 63(1), e12294.

Tizzard, A.C., and Lloyd-Jones, G. (2007). Bacterial oxygenases: in vivo enzyme biosensors for organic pollutants. Biosens Bioelectron 22, 2400-07.

Toda, C., Uchida, T., Midorikawa, K., Murata, M., Hiraku, Y., Okamoto, Y., Ueda, K., Kojima, N., and Kawanishi, S. (2003). DNA damage by ethylbenzenehydroperoxide formed from carcinogenic ethylbenzene by sunlight irradiation. Biochem Biophys Res Commun 304, 638-642.

Tran, K. V., Casey, J. A., Cushing, L. J., & Morello-Frosch, R. (2020). Residential Proximity to Oil and Gas Development and Birth Outcomes in California: A Retrospective Cohort Study of 2006-2015 Births. Environ Health Perspect, 128(6), 67001.

Tsangari X, Andrianou XD, Agapiou A, Mochalski P, Makris KC. 2017. Spatial characteristics of urinary BTEX concentrations in the general population. Chemosphere 173: 261–6.

Ueda, K. (2009). Effect of Environmental Chemicals on Genes and the Expression. Yakugaku Zasshi 129, 1501-06.

Ungváry, G., and Tátrai, E. (1985). On the embryotoxic effects of benzene and its alkyl derivatives in mice, rats and rabbits. Arch Toxicol Suppl 8, 425-430.

Valcke M, Haddad S. 2015. Assessing human variability in kinetics for exposures to multiple environmental chemicals: a physiologically based pharmacokinetic modeling case study with dichloromethane, benzene, toluene, ethylbenzene, and m-xylene. J Toxicol Environ Health A 78: 409–31.

Vigano, L. (1993). Reproductive strategy of Daphnia magna and toxicity of organic compounds. Water Res 27, 903-09.

Wang T, Bo P, Bing T, Zhaoyun Z, Liyu D, Yonglong L. 2014. Benzene homologues in environmental matrixes from a pesticide chemical region in China: Occurrence, health risk and management. Ecotoxicol Environ Saf 104: 357–64.

Wathier L, Venet T, Thomas A, Nunge H, Bonfanti E, Cosnier F, Parietti-Winkler C, Campo P, Tsan P, Bouguet-Bonnet S, Gansmuller A. 2016. Membrane fluidity does not explain how solvents act on the middle-ear reflex. Neurotoxicology 57: 13–21.

Ethylbenzene References Reviewed
Supplemental Material A

Webb E, Bushkin-Bedient S, Cheng A, Kassotis CD, Balise V, Nagel SC. 2014. Developmental and reproductive effects of chemicals associated with unconventional oil and natural gas operations. Rev Environ Health 29: 307–18.

Webb E, Hays J, Dyrszka L, Rodriguez B, Cox C, Huffling K, Bushkin-Bedient S. 2016. Potential hazards of air pollutant emissions from unconventional oil and natural gas operations on the respiratory health of children and infants. Rev Environ Health 31: 225–43.

Webb E, Moon J, Dyrszka L, Rodriguez B, Cox C, Patisaul H, Bushkin S, London E. 2018. Neurodevelopmental and neurological effects of chemicals associated with unconventional oil and natural gas operations and their potential effects on infants and children. Rev Environ Health 33: 3–29.

Weinstein JR, Asteria-Penaloza R, Diaz-Artiga A, Davila G, Hammond SK, Ryde IT, Meyer JN, Benowitz N, Thompson LM. 2017. Exposure to polycyclic aromatic hydrocarbons and volatile organic compounds among recently pregnant rural Guatemalan women cooking and heating with solid fuels. Int J Hyg Environ Health 220: 726–35.

Werder, E. J., Beier, J. I., Sandler, D. P., Falkner, K. C., Gripshover, T., Wahlang, B., Engel, L. S., & Cave, M. C. (2020). Blood BTEXS and heavy metal levels are associated with liver injury and systemic inflammation in Gulf states residents. Food Chem Toxicol, 139, 111242.

Werder, E. J., Engel, L. S., Blair, A., Kwok, R. K., McGrath, J. A., & Sandler, D. P. (2019). Blood BTEX levels and neurologic symptoms in Gulf states residents. Environ Res, 175, 100-107.

Wickliffe, J. K., Stock, T. H., Howard, J. L., Frahm, E., Simon-Friedt, B. R., Montgomery, K., Wilson, M. J., Lichtveld, M. Y., & Harville, E. (2020). Increased long-term health risks attributable to select volatile organic compounds in residential indoor air in southeast Louisiana. Sci Rep, 10(1), 21649.

Xiong F, Li Q, Zhou B, Huang J, Liang G, Zhang L, Ma S, Qing L, Liang L, Su J, Peng X, Li Q, Zou Y. 2016. Oxidative Stress and Genotoxicity of Long-Term Occupational Exposure to Low Levels of BTEX in Gas Station Workers. Int J Environ Res Public Health 13:

Xu, J., Zheng, L., Yan, Z., Huang, Y., Feng, C., Li, L., & Ling, J. (2020). Effective extrapolation models for ecotoxicity of benzene, toluene, ethylbenzene, and xylene (BTEX). Chemosphere, 240, 124906.

Xu, Z., Mulchandani, A., and Chen, W. (2003). Detection of benzene, toluene, ethyl benzene, and xylenes (BTEX) using toluene dioxygenase-peroxidase coupling reactions. Biotechnol Prog 19, 1812-15.

Yamada, A., Kishi, H., Sugiyama, K., Hatta, T., Nakamura, K., Masai, E., and Fukuda, M. (1998). Two nearly identical aromatic compound hydrolase genes in a strong polychlorinated biphenyl degrader, Rhodococcus sp. strain RHA1. Appl Environ Microbiol 64, 2006-012.

Yang, R. (1993). NTP technical report on the toxicity studies of a Chemical Mixture of 25 Groundwater Contaminants Administered in Drinking Water to F344/N Rats and B6C3F(1) Mice. Toxic Rep Ser 35, 1-I12.

Yousefian, F., Mahvi, A. H., Yunesian, M., Hassanvand, M. S., Kashani, H., & Amini, H. (2018). Long-term exposure to ambient air pollution and autism spectrum disorder in children: A case-control study in Tehran, Iran. Sci Total Environ, 643, 1216-1222.

Yu, S. Y., Koh, E. J., Kim, S. H., Lee, S. Y., Lee, J. S., Son, S. W., & Hwang, S. Y. (2021). Integrated analysis of multi-omics data on epigenetic changes caused by combined exposure to environmental hazards. Environ Toxicol, 36(6), 1001-1010.

Zapór, L., Skowroń, J., and Gołofit-Szymczak, M. (2002). The cytotoxicity of some organic solvents on isolated hepatocytes in monolayer culture. Int J Occup Saf Ergon 8, 121-29.

Zhang M, Wang Y, Wang X, Liu J, Zhang J, Gu Q. 2016. Roles of oxidative stress, apoptosis, and heme oxygenase-1 in ethylbenzene-induced renal toxicity in NRK-52E cells. Toxicol Ind Health 32: 1952–60.

Zhang M, Wang Y, Yang D, Zhang J, Gu Q. 2015. Roles of oxidative damage and mitochondria-mediated apoptosis in ethylbenzene-induced hepatotoxic effects in rat. Inhal Toxicol 27: 64–73.

Zhang, L., Zhang, C., Cheng, Z., Yao, Y., and Chen, J. (2013). Biodegradation of benzene, toluene, ethylbenzene, and o-xylene by the bacterium Mycobacterium cosmeticum byf-4. Chemosphere 90, 1340-47.

Zhang, M., Wang, Y., Wang, Q., Yang, J., Yang, D., Liu, J., and Li, J. (2010). Involvement of mitochondria-mediated apoptosis in ethylbenzene-induced renal toxicity in rat. Toxicol Sci 115, 295-303.

Zhang, S., Cawley, G.F., Eyer, C.S., and Backes, W.L. (2002). Altered ethylbenzene-mediated hepatic CYP2E1 expression in growth hormone-deficient dwarf rats. Toxicol Appl Pharmacol 179, 74-82.

Zheng S, Zhou Q. 2017. Intoxication and biochemical responses of freshwater snail Bellamya aeruginosa to ethylbenzene. Environ Sci Pollut Res Int 24: 189–98.

Zheng, S., Wang, Y., Zhou, Q., and Chen, C. (2013). Responses of oxidative stress biomarkers and DNA damage on a freshwater snail (Bellamya aeruginosa) stressed by ethylbenzene. Arch Environ Contam Toxicol 65, 251-59.

Table of Contents

Supplemental Material B 5

OSRI Evaluation for Ethylbenzene 5

Summaries of Studies 5

1. Estrogen Agonist Hypothesis 5

1.1 Rank 2: Repeat Dose Toxicity – Epididymis histopathology 5

1.2 Rank 2: Repeat Dose Toxicity – Epididymis weight 6

1.3 Rank 2: Repeat Dose Toxicity – Ovary histopathology 6

1.4 Rank 2: Repeat Dose Toxicity – Testis histopathology (atrophy) 7

1.5 Rank 2: Repeat Dose Toxicity – Testis weight 8

1.6 Rank 2: Repeat Dose Toxicity – Uterus histopathology 8

1.7 Rank 2: Repeat Dose Toxicity – Vaginal histopathology 9

1.8 Rank 2: Developmental Toxicity – Corpora lutea 9

1.9 Rank 2: Developmental Toxicity – Post-implantation loss 10

1.10 Rank 2: Developmental Toxicity – Pre-implantation loss 11

1.11 Rank 2: Reproductive Toxicity – Estrous cyclicity 12

1.12 Rank 2: Reproductive Toxicity – Fertility 12

1.13 Rank 2: Developmental Toxicity – Gestational length 13

1.14 Rank 2: Reproductive Toxicity – Implantations 13

1.15 Rank 2: Reproductive Toxicity – Litter size 14

1.16 Rank 2: Reproductive Toxicity – Mating index 14

1.17 Rank 2: Reproductive Toxicity – Ovarian follicle count in offspring 14

1.18 Rank 2: Reproductive Toxicity – Sperm count 15

1.19 Rank 2: Reproductive Toxicity – Time to mating 15

1.20 Rank 2: Reproductive Toxicity – Time to vaginal patency 16

1.21 Rank 3: Repeat Dose Toxicity – Gross pathology 16

2. Estrogen Antagonist Hypothesis 17

2.1 Rank 2: Repeat Dose Toxicity – Epididymis histopathology 17

2.2 Rank 2: Repeat Dose Toxicity – Ovary histopathology 18

2.3 Rank 2: Repeat Dose Toxicity – Prostate histopathology 19

2.4 Rank 2: Repeat Dose Toxicity – Seminal vesicle histopathology 19

2.5 Rank 2: Repeat dose toxicity – Testis histopathology (atrophy) 20

2.6 Rank 2: Repeat Dose Toxicity – Testis weight ... 21
2.7 Rank 2: Developmental Toxicity – Corpora lutea ... 22
2.8 Rank 2: Reproductive Toxicity – Estrous cyclicity ... 22
2.9 Rank 2: Reproductive Toxicity – Fertility ... 23
2.10 Rank 2: Reproductive Toxicity – Litter size ... 23
2.11 Rank 2: Reproductive Toxicity – Sperm count ... 23
2.12 Rank 2: Reproductive Toxicity – Time to mating ... 24
2.13 Rank 2: Reproductive Toxicity – Time to vaginal patency in offspring ... 24
2.14 Rank 3: Repeat Dose Toxicity – Gross Pathology ... 25
3. Androgen Agonist Hypothesis ... 25
3.1 Rank 2: Repeat Dose Toxicity – Ovary histopathology ... 25
3.2 Rank 2: Repeat Dose Toxicity – Sperm count ... 26
3.3 Rank 2: Repeat dose toxicity – Testis histopathology (atrophy) ... 26
3.4 Rank 2: Repeat Dose Toxicity – Testis weight ... 27
3.5 Rank 2: Developmental Toxicity – Implantations ... 28
3.6 Rank 2: Developmental Toxicity – Litter size ... 29
3.7 Rank 2: Developmental Toxicity – Sex ratio ... 30
3.8 Rank 2: Reproductive Toxicity – Estrous cyclicity ... 30
3.9 Rank 2: Reproductive Toxicity – Fertility ... 31
3.10 Rank 2: Reproductive Toxicity – Implantations ... 31
3.11 Rank 2: Reproductive Toxicity – Litter size ... 32
3.12 Rank 2: Reproductive Toxicity – Mating index ... 32
3.13 Rank 2: Reproductive Toxicity – Prostate weight ... 33
3.14 Rank 2: Reproductive Toxicity – Sex ratio ... 33
3.15 Rank 2: Reproductive Toxicity – Sperm count ... 33
3.16 Rank 2: Reproductive Toxicity – Time to balano-preputial separation ... 34
3.17 Rank 2: Reproductive Toxicity – Time to mating ... 34
3.18 Rank 2: Reproductive Toxicity – Time to vaginal patency ... 35
3.19 Rank 3: Repeat Dose Toxicity – Gross pathology ... 35
4. Androgen Antagonist Hypothesis ... 36
4.1 Rank 2: Repeat Dose Toxicity – Epididymal weight ... 36
4.2 Rank 2: Repeat Dose Toxicity – Epididymis histopathology ... 36
4.3 Rank 2: Repeat Dose Toxicity – Ovary histopathology ... 37
4.4 Rank 2: Repeat Dose Toxicity – Prostate histopathology ... 38

4.5 Rank 2: Repeat Dose Toxicity – Seminal vesicle histopathology ... 39
4.6 Rank 2: Repeat Dose – Testis histopathology (atrophy) ... 40
4.7 Rank 2: Repeat Dose Toxicity – Testis weight ... 41
4.8 Rank 2: Repeat Dose Toxicity – Uterus histopathology ... 41
4.9 Rank 2: Reproductive Toxicity – Estrous cyclicity ... 42
4.10 Rank 2: Reproductive Toxicity – Fertility ... 42
4.11 Rank 2: Reproductive Toxicity – Gross pathology ... 43
4.12 Rank 2: Reproductive Toxicity – Litter size ... 43
4.13 Rank 2: Reproductive Toxicity – Prostate weight ... 44
4.14 Rank 2: Reproductive Toxicity – Sperm count ... 44
4.15 Rank 2: Reproductive Toxicity – Sperm motility ... 45
4.16 Rank 2: Reproductive Toxicity – Time to balano-preputial separation ... 45
4.17 Rank 2: Reproductive Toxicity – Time to mating ... 46
4.18 Rank 3: Repeat Dose Toxicity – Gross pathology ... 46
5. Thyroid Inhibition Hypothesis ... 46
5.1 Rank 2: Repeat Dose Toxicity – Thyroid follicular cell histopathology ... 46
5.2 Rank 2: Developmental Toxicity – Fetal survival ... 47
5.3 Rank 2: Developmental Toxicity – Fetal weight ... 49
5.4 Rank 2: Reproductive Toxicity – Pup growth ... 50
5.5 Rank 2: Reproductive Toxicity – Pup survival ... 51
5.6 Rank 2: Reproductive Toxicity – Thyroid weight ... 51
5.7 Rank 3: Repeat Dose Toxicity – Liver weight ... 52
5.8 Rank 3: Reproductive Toxicity – Liver weight ... 53
5.9 Rank 3: Developmental Neurotoxicity – Auditory startle ... 54
5.10 Rank 3: Developmental Neurotoxicity – Brain morphometry ... 55
5.11 Rank 3: Developmental Neurotoxicity – Learning and memory ... 55
5.12 Rank 3: Developmental Neurotoxicity – Motor activity ... 56
6. Interaction with Steroidogenesis Enzymes Hypothesis ... 56
6.1 Rank 2: Repeat Dose Toxicity – Ovary histopathology ... 56
6.2 Rank 2: Repeat Dose toxicity – Testis histopathology ... 57
6.3 Rank 2: Repeat Dose Toxicity – Uterus histopathology ... 58
6.4 Rank 2: Developmental Toxicity – Sex ratio ... 59
6.5 Rank 2: Reproductive Toxicity – Estrous cyclicity ... 60
6.6 Rank 2: Reproductive Toxicity – Fertility ... 60

6.7 Rank 2: Reproductive Toxicity – Live births **61**
6.8 Rank 2: Reproductive Toxicity – Mating index **61**
6.9 Rank 2: Reproductive Toxicity – Sex ratio **62**
6.10 Rank 2: Reproductive Toxicity –Sperm count **62**
6.11 Rank 3: Repeat Dose Toxicity – Gross Pathology **63**

Supplemental Material B
OSRI Evaluation for Ethylbenzene

Summaries of Studies

1. Estrogen Agonist Hypothesis

1.1 Rank 2: Repeat Dose Toxicity – Epididymis histopathology

[1] ***NTP (National Toxicology Program). (1999)*** Groups of 50 male and 50 female Fischer rats and B6C3F1 mice were exposed to ethylbenzene by inhalation at 0, 75, 250, and 750 ppm 6 h per day, 5 days per week, for 104 weeks (rats) and for 103 week (mice).

Limitations: This study was not specifically designed to investigate an endocrine mode of action. Although a negative result supports the conclusion that the response is not indicative of the hypothesis, a positive result could be due to several mechanisms.

Results included in WOE: There were no significant histological differences between the chamber control animals and treatment group animals, in either specie, for uterine, vaginal or ovarian tissues in females or for testes, prostate, epididymides or seminal vesicles for males.

[3] ***Mellert and colleagues (2007)*** Ethylbenzene was administered to groups of male and female Wistar rats by gavage for 4 (*n* = 5/ dose/sex) and 13 weeks (*n* = 10/dose/sex) (OECD 408) at doses of 0 (vehicle control), 75, 250, and 750 mg/kg bw/day, administered am/pm as half doses.

Limitations: This study was not specifically designed to investigate an endocrine mode of action. Although a negative result supports the conclusion that the response is not indicative of the hypothesis, a positive result could be due to several mechanisms.

Results included in WOE: Histopathological evaluation of the epididymis, mammary glands, ovaries, prostate, seminal vesicles, testes, thyroid, uterus, and vagina did not identify any adverse effects.

[9] ***NTP (National Toxicology Program). Toxicity studies of ethylbenzene (CAS No. 100-41-4) in F344/N rats and B6C3F1 mice (inhalation studies) (1992)*** Inhalation toxicity of ethylbenzene was studied by exposing groups of seven-week-old F344/N rats and B6C3Fi mice of each sex to ethylbenzene vapor at chamber concentrations of 0, 100, 250, 500, 750, or 1000 ppm, 6 h per day, 5 days per week for 13 weeks.

Limitations: This study was not specifically designed to investigate an endocrine mode of action. Although a negative result supports the conclusion that the response is not indicative of the hypothesis, a positive result could be due to several mechanisms.

Results included in WOE: There were no histologic changes in the epididymides of ethylbenzene-exposed mice or rats.

[10] Cragg and colleagues (1989) Mice, rats and rabbits (five/sex/group) were exposed by inhalation to ethylbenzene vapors for 6 h/day, 5 days/week for 4 weeks (20 exposures). Rats and mice received 0, 99, 382 or 782 ppm EB while rabbits received 0, 382, 782 or 1610 ppm.

Limitations: This study was not specifically designed to investigate an endocrine mode of action. Although a negative result supports the conclusion that the response is not indicative of the hypothesis, a positive result could be due to several mechanisms.

Results included in WOE: The gonads (ovaries or testes with epididymides) and thyroids of high-exposure and controls animals of all species were subjected to histopathological examination. There were no gross or microscopic changes attributable to ethylbenzene in any of these tissues.

1.2 Rank 2: Repeat Dose Toxicity – Epididymis weight

[9] ***NTP (National Toxicology Program). Toxicity studies of ethylbenzene (CAS No. 100-41-4) in F344/N rats and B6C3F1 mice (inhalation studies) (1992)*** Inhalation toxicity of ethylbenzene was studied by exposing groups of seven-week-old F344/N rats and B6C3Fi mice of each sex to ethylbenzene vapor at chamber concentrations of 0, 100, 250, 500, 750, or 1000 ppm, 6 h per day, 5 days per week for 13 weeks.

Limitations: This study was not specifically designed to investigate an endocrine mode of action. Although a negative result supports the conclusion that the response is not indicative of the hypothesis, a positive result could be due to several mechanisms. Additionally, high doses were used. It is unclear if the endpoint responses are secondary to general toxicity.

Results included in WOE: The tabular data indicate there was a significant decrease in the epididymal weight in mice exposed to ethylbenzene in the 1000-ppm group. The authors note that this was not considered biologically significant since spermatid counts, sperm motility, and caudal weight were normal. The narrative portion of the report states that this significant difference was found in the epididymal weight of rats, not mice – a likely error (p. 17). The tabular data show that there was no difference in the epididymal weight of rats at any ethylbenzene exposure level.

1.3 Rank 2: Repeat Dose Toxicity – Ovary histopathology

[1] ***NTP (National Toxicology Program). (1999)*** Groups of 50 male and 50 female Fischer rats and B6C3F1 mice were exposed to ethylbenzene by inhalation at 0, 75, 250, and 750 ppm 6 h per day, 5 days per week, for 104 weeks (rats) and for 103 week (mice).

Limitations: This study was not specifically designed to investigate an endocrine mode of action. Although a negative result supports the conclusion that the response is not indicative of the hypothesis, a positive result could be due to several mechanisms.

Results included in WOE: There were no significant histological differences between the chamber control animals and treatment group animals, in either specie, for uterine, vaginal or ovarian tissues in females or for testes, prostate, epididymides or seminal vesicles for males.

[3] ***Mellert and colleagues (2007)*** Ethylbenzene was administered to groups of male and female Wistar rats by gavage for 4 (n = 5/ dose/sex) and 13 weeks (n = 10/dose/sex) (OECD 408) at doses of 0 (vehicle control), 75, 250, and 750 mg/kg bw/day, administered am/pm as half doses.

Limitations: This study was not specifically designed to investigate an endocrine mode of action. Although a negative result supports the conclusion that the response is not indicative of the hypothesis, a positive result could be due to several mechanisms.

Results included in WOE: Histopathological evaluation of the epididymis, mammary glands, ovaries, prostate, seminal vesicles, testes, thyroid, uterus, and vagina did not identify any adverse effects.

[9] ***NTP (National Toxicology Program). Toxicity studies of ethylbenzene (CAS No. 100-41-4) in F344/N rats and B6C3F1 mice (inhalation studies) (1992)*** Inhalation toxicity of ethylbenzene was studied by exposing groups of seven-week-old F344/N rats and B6C3Fi mice of each sex to ethylbenzene vapor at chamber concentrations of 0, 100, 250, 500, 750, or 1000 ppm, 6 h per day, 5 days per week for 13 weeks.

Limitations: This study was not specifically designed to investigate an endocrine mode of action. Although a negative result supports the conclusion that the response is not indicative of the hypothesis, a positive result could be due to several mechanisms.

Results included in WOE: There were no histopathologic lesions and no chemically related histopathologic changes identified in the ovaries of mice or rats compared with controls.

[10] Cragg and colleagues (1989) Mice, rats and rabbits (five/sex/group) were exposed by inhalation to ethylbenzene vapors for 6 h/day, 5 days/week for 4 weeks (20 exposures). Rats and mice received 0, 99, 382 or 782 ppm EB while rabbits received 0, 382, 782 or 1610 ppm.

Limitations: This study was not specifically designed to investigate an endocrine mode of action. Although a negative result supports the conclusion that the response is not indicative of the hypothesis, a positive result could be due to several mechanisms.

Results included in WOE: The gonads (ovaries or testes with epididymides) and thyroids of high-exposure and controls animals of all species were subjected to histopathological examination. There were no gross or microscopic changes attributable to ethylbenzene in any of these tissues.

1.4 Rank 2: Repeat Dose Toxicity – Testis histopathology (atrophy)

[1] ***NTP (National Toxicology Program). (1999)*** Groups of 50 male and 50 female Fischer rats and B6C3F1 mice were exposed to ethylbenzene by inhalation at 0, 75, 250, and 750 ppm 6 h per day, 5 days per week, for 104 weeks (rats) and for 103 week (mice).

Limitations: This study was not specifically designed to investigate an endocrine mode of action. Although a negative result supports the conclusion that the response is not indicative of the hypothesis, a positive result could be due to several mechanisms.

Results included in WOE: There were no significant histological differences between the chamber control animals and treatment group animals, in either specie, for uterine, vaginal or ovarian tissues in females or for testes, prostate, epididymides or seminal vesicles for males.

[9] ***NTP (National Toxicology Program). Toxicity studies of ethylbenzene (CAS No. 100-41-4) in F344/N rats and B6C3F1 mice (inhalation studies) (1992)*** Inhalation toxicity of ethylbenzene was studied by exposing groups of seven-week-old F344/N rats and B6C3Fi mice of each sex to ethylbenzene vapor at chamber concentrations of 0, 100, 250, 500, 750, or 1000 ppm, 6 h per day, 5 days per week for 13 weeks.

Limitations: This study was not specifically designed to investigate an endocrine mode of action. Although a negative result supports the conclusion that the response is not indicative of the hypothesis, a positive result could be due to several mechanisms.

Results included in WOE: There were no effects observed on sperm or testicular morphology in rats exposed to ethylbenzene.

1.5 Rank 2: Repeat Dose Toxicity – Testis weight

[9] ***NTP (National Toxicology Program). Toxicity studies of ethylbenzene (CAS No. 100-41-4) in F344/N rats and B6C3F1 mice (inhalation studies) (1992)*** Inhalation toxicity of ethylbenzene was studied by exposing groups of seven-week-old F344/N rats and B6C3Fi mice of each sex to ethylbenzene vapor at chamber concentrations of 0, 100, 250, 500, 750, or 1000 ppm, 6 h per day, 5 days per week for 13 weeks.

Limitations: This study was not specifically designed to investigate an endocrine mode of action. Although a negative result supports the conclusion that the response is not indicative of the hypothesis, a positive result could be due to several mechanisms.

Results included in WOE: Weights of testes in mice and rats were not affected by ethylbenzene.

1.6 Rank 2: Repeat Dose Toxicity – Uterus histopathology

[1] ***NTP (National Toxicology Program). (1999)*** Groups of 50 male and 50 female Fischer rats and B6C3F1 mice were exposed to ethylbenzene by inhalation at 0, 75, 250, and 750 ppm 6 h per day, 5 days per week, for 104 weeks (rats) and for 103 week (mice).

Limitations: This study was not specifically designed to investigate an endocrine mode of action. Although a negative result supports the conclusion that the response is not indicative of the hypothesis, a positive result could be due to several mechanisms.

Results included in WOE: There were no significant histological differences between the chamber control animals and treatment group animals, in either specie, for uterine, vaginal or ovarian tissues in females or for testes, prostate, epididymides or seminal vesicles for males.

[3] ***Mellert and colleagues (2007)*** Ethylbenzene was administered to groups of male and female Wistar rats by gavage for 4 (n = 5/ dose/sex) and 13 weeks (n = 10/dose/sex) (OECD 408) at doses of 0 (vehicle control), 75, 250, and 750 mg/kg bw/day, administered am/pm as half doses.

Limitations: This study was not specifically designed to investigate an endocrine mode of action. Although a negative result supports the conclusion that the response is not indicative of the hypothesis, a positive result could be due to several mechanisms.

Results included in WOE: Histopathological evaluation of the epididymis, mammary glands, ovaries, prostate, seminal vesicles, testes, thyroid, uterus, and vagina did not identify any adverse effects.

[9] ***NTP (National Toxicology Program). Toxicity studies of ethylbenzene (CAS No. 100-41-4) in F344/N rats and B6C3F1 mice (inhalation studies) (1992)*** Inhalation toxicity of ethylbenzene was studied by exposing groups of seven-week-old F344/N rats and B6C3Fi mice of each sex to

ethylbenzene vapor at chamber concentrations of 0, 100, 250, 500, 750, or 1000 ppm, 6 h per day, 5 days per week for 13 weeks.

Limitations: This study was not specifically designed to investigate an endocrine mode of action. Although a negative result supports the conclusion that the response is not indicative of the hypothesis, a positive result could be due to several mechanisms.

Results included in WOE: There were no histopathologic lesions and no chemically related histopathologic changes identified in the uterus of mice or rats compared with controls.

[10] ***Cragg and colleagues (1989)*** Mice, rats and rabbits (five/sex/group) were exposed by inhalation to ethylbenzene vapors for 6 h/day, 5 days/week for 4 weeks (20 exposures). Rats and mice received 0, 99, 382 or 782 ppm EB while rabbits received 0, 382, 782 or 1610 ppm.

Limitations: This study was not specifically designed to investigate an endocrine mode of action. Although a negative result supports the conclusion that the response is not indicative of the hypothesis, a positive result could be due to several mechanisms.

Results included in WOE: The uterus of high-exposure and controls animals of all species was subjected to histopathological examination. There were no gross or microscopic changes attributable to ethylbenzene in this organ.

1.7 Rank 2: Repeat Dose Toxicity – Vaginal histopathology

[1] NTP (National Toxicology Program). (1999) Groups of 50 male and 50 female Fischer rats and B6C3F1 mice were exposed to ethylbenzene by inhalation at 0, 75, 250, and 750 ppm 6 h per day, 5 days per week, for 104 weeks (rats) and for 103 week (mice).

Limitations: This study was not specifically designed to investigate an endocrine mode of action. Although a negative result supports the conclusion that the response is not indicative of the hypothesis, a positive result could be due to several mechanisms.

Results used for WOE: The histological examination of vaginal tissue did not reveal significant differences between the chamber controls and any of the exposure groups in rats or mice.

[3] Mellert and colleagues (2007) Ethylbenzene was administered to groups of male and female Wistar rats by gavage for 4 (*n* = 5/ dose/sex) and 13 weeks (*n* = 10/dose/sex) (OECD 408) at doses of 0 (vehicle control), 75, 250, and 750 mg/kg bw/day, administered am/pm as half doses.

Limitations: This study was not specifically designed to investigate an endocrine mode of action. Although a negative result supports the conclusion that the response is not indicative of the hypothesis, a positive result could be due to several mechanisms.

Results included in WOE: Histopathological evaluation of the epididymis, mammary glands, ovaries, prostate, seminal vesicles, testes, thyroid, uterus, and vagina did not identify any adverse effects.

1.8 Rank 2: Developmental Toxicity – Corpora lutea

[5] ***Saillenfait and colleagues (2003)*** The developmental toxicity of ethylbenzene was studied in

Sprague–Dawley rats after inhalation exposure. Animals were exposed to ethylbenzene at 100, 500, 1000 or 2000 ppm, for 6 h/day, during days 6–20 of gestation.

Limitations: This study was not specifically designed to investigate an endocrine mode of action. Although a negative result supports the conclusion that the response is not indicative of the hypothesis, a positive result could be due to several mechanisms.

Results included in WOE: The mean number of corpora lutea per dam did not differ between dams in any of the treatment groups and control dams.

[13] ***Andrew et al., (1981); Hardin et al. (1981)*** Groups of 29-33 Female Wistar rats and New Zealand White rabbits were exposed to 0, 100, or 1000 ppm ethylbenzene for 7 h/day, 5 days/week for 3 weeks, then mated with unexposed males. Pregnant females were further exposed to 0, 100, or 1000 ppm 7 h/day through Gestational Day 19 (rats) and 24 (rabbits).

Limitations: This study was not specifically designed to investigate an endocrine mode of action. Although a negative result supports the conclusion that the response is not indicative of the hypothesis, a positive result could be due to several mechanisms.

Results included in the WOE: The number of Corpora Lutea was unchanged by exposure to 100 and 1,000 ppm ethylbenzene relative to controls.

1.9 Rank 2: Developmental Toxicity – Post-implantation loss

[4] ***Saillenfait and colleagues (2007)*** The combined effects of EB and butyl acetate (BA) were investigated. Groups of 18 bred rats (15– 18 pregnant) were exposed to vapors of EB or BA, separately or in combination, 6 h day−1, on days 6–20 of gestation. There were nine experimental groups: Control; 250 or 1000 ppm EB; 500 or 1500 ppm BA or mixtures of 250 ppm EB + 500 ppm BA, 250 ppm EB + 1500 ppm BA, 1000 ppm EB + 500 ppm BA, or 1000 ppm EB + 1500 ppm BA

Limitations: This study was not specifically designed to investigate an endocrine mode of action. Although a negative result supports the conclusion that the response is not indicative of the hypothesis, a positive result could be due to several mechanisms.

Results included in WOE: There was no effect of treatment on the mean number of implantations and of live fetuses, and on the incidence of non-live implants and resorptions.

[5] ***Saillenfait and colleagues (2003)*** The developmental toxicity of ethylbenzene was studied in Sprague–Dawley rats after inhalation exposure. Animals were exposed to ethylbenzene at 100, 500, 1000 or 2000 ppm, for 6 h/day, during days 6–20 of gestation.

Limitations: Clinical signs of toxicity (ataxia, decreased motor activity) were seen at 2000 ppm. Maternal weight was significantly reduced on GD 21 at 1000 ppm and on GD 13 and 21 at 2000 ppm. Dams exposed to 1000 or 2000 ppm showed significant decreases in maternal weight gain and food consumption throughout exposure, and in corrected weight gain

Results included in WOE: The number of implantations was comparable among groups.
Although the difference was not statistically significant, the incidence of non-live implants and resorptions was higher at 2000 ppm than in the control group. This was likely due to the 100% postimplantation loss seen in three of the 21 pregnant females exposed to 2000 ppm (0 in other

groups).

[6] ***Saillenfait and colleagues (2006)*** Pregnant Sprague–Dawley rats were exposed to ethylbenzene (EB; 0, 250, or 1000 ppm) and methylethylketone (MEK; 0, 1000, or 3000 ppm), alone and in combination, by inhalation, for 6 h/day, during days 6–20 of gestation.

Limitations: This study was not specifically designed to investigate an endocrine mode of action. Although a negative result supports the conclusion that the response is not indicative of the hypothesis, a positive result could be due to several mechanisms.

Results included in WOE: EB and MEK, alone or in combination, have no effect on the average number of implantations and live fetuses, and in the incidence of non-live implants and resorptions.

[11] ***Ungváry, G. & Tátrai, E. (1985)*** Groups of CFY rats were exposed to inhalation of ethylbenzene at 0, 138, 276 or 553 ppm for 24 h/day from day 7 to day 15 of pregnancy. Fetuses were evaluated on pregnancy day 21. CFLP mice were exposed to inhalation of ethylbenzene at 0, 115 or 230 ppm for 24 h/day (no data provided for these groups) or for 3-4 hours/day intermittently from day 6 to 15 of pregnancy. The fetuses were evaluated on pregnancy days 18. NZ rabbits were exposed to 0, 115, or 230 ppm ethylbenzene for 24 h/day from day 7 to day 20 gestation. Fetuses were examined on pregnancy day 30. The three rabbit does in the 230-ppm dose group aborted.

Limitations: The data for mice was only provided for the animals in the 115-ppm exposure group and maternal toxicity information was lacking. The authors mention that the maternal toxic effects of ethylbenzene in rats were "moderate and dose-dependent" but fail to describe or quantify these effects. The contribution of general toxicity effects to all study findings should be considered.

Results included in WOE: The percentage of dead or resorbed fetuses was significantly increased in all ethylbenzene-exposed groups in rats (138, 276 and 553 ppm). There was no significant difference in the percentage of dead or resorbed fetuses in ethylbenzene exposed mice or rabbits compared with controls.

[13] ***Andrew et al., (1981); Hardin et al. (1981)*** Groups of 29-33 Female Wistar rats and New Zealand White rabbits were exposed to 0, 100, or 1000 ppm ethylbenzene for 7 h/day, 5 days/week for 3 weeks, then mated with unexposed males. Pregnant females were further exposed to 0, 100, or 1000 ppm 7 h/day through Gestational Day 19 (rats) and 24 (rabbits).

Limitations: This study was not specifically designed to investigate an endocrine mode of action. Although a negative result supports the conclusion that the response is not indicative of the hypothesis, a positive result could be due to several mechanisms.

Results included in the WOE: The number of implantations was comparable among groups. Post-implantation loss was inferred from the number of live fetuses, which was slightly reduced in rabbits, but not in rats, exposed to ethylbenzene at 1,000 ppm, a concentration that produced some indications of maternal systemic effects. This finding is therefore unlikely to have been produced by and endocrine mode of action.

1.10 Rank 2: Developmental Toxicity – Pre-implantation loss

[13] ***Andrew et al., (1981); Hardin et al. (1981)*** Groups of 29-33 Female Wistar rats and New Zealand White rabbits were exposed to 0, 100, or 1000 ppm ethylbenzene for 7 h/day, 5 days/week for

3 weeks, then mated with unexposed males. Pregnant females were further exposed to 0, 100, or 1000 ppm 7 h/day through Gestational Day 19 (rats) and 24 (rabbits).

Limitations: This study was not specifically designed to investigate an endocrine mode of action. Although a negative result supports the conclusion that the response is not indicative of the hypothesis, a positive result could be due to several mechanisms.

Results included in the WOE: Pre-implantation loss was inferred from the number of implantations per corpora lutea, which was comparable between control groups and groups exposed to 100 and 1,000 ppm ethylbenzene in both rats and rabbits.

1.11 Rank 2: Reproductive Toxicity – Estrous cyclicity

[2] ***Faber and colleagues (2006)*** Four groups of Crl:CD(SD)IGS BR rats (30/sex/group for F_0 and 25/sex/group for F_1) were exposed to 0, 25, 100, and 500ppm EB for 6 h/day for at least 70 consecutive days before mating. Inhalation exposure for the F_0 and F_1 females continued throughout mating, gestation through gestation day (GD) 20, and lactation days (LD) 5–21. On LD 1–4, females received EB in corn oil via oral gavage at dose levels of 26, 90, and 342 mg/kg/day (divided into three equal doses, approximately 2 h apart). Pups were weaned on postnatal day (PND) 21 and exposure of the F_1 generation started on PND 22. Estimates of internal exposure were determined by measuring EB concentrations in blood collected from F_1 dams (4/group) and their culled pups 1 h after the last gavage dose on PND 4. On PND 22, blood was collected from these same F_1 dams and their weanlings for EB analysis 1 h after a 6-h inhalation exposure. The remainder of the F_2 generation was not directly exposed.

Limitations: This study was not specifically designed to investigate an endocrine mode of action. Although a negative result supports the conclusion that the response is not indicative of the hypothesis, a positive result could be due to several mechanisms.

Results included in WOE: The mean estrous cycle length (4.0 ± 0.3 days) was significantly reduced for the F_0, 500 ppm group when compared to the F_0 control group value (4.4 ± 0.8 days). However, the authors felt this difference was not biologically important because all females in this group were cycling normally and this strain of rat normally exhibits 4- to 5-day estrous cycles. Mean estrous cycle length did not differ between control and experimental F_1 offspring.

1.12 Rank 2: Reproductive Toxicity – Fertility

[2] ***Faber and colleagues (2006)*** Four groups of Crl:CD(SD)IGS BR rats (30/sex/group for F_0 and 25/sex/group for F_1) were exposed to 0, 25, 100, and 500ppm EB for 6 h/day for at least 70 consecutive days before mating. Inhalation exposure for the F_0 and F_1 females continued throughout mating, gestation through gestation day (GD) 20, and lactation days (LD) 5–21. On LD 1–4, females received EB in corn oil via oral gavage at dose levels of 26, 90, and 342 mg/kg/day (divided into three equal doses, approximately 2 h apart). Pups were weaned on postnatal day (PND) 21 and exposure of the F_1 generation started on PND 22. Estimates of internal exposure were determined by measuring EB concentrations in blood collected from F_1 dams (4/group) and their culled pups 1 h after the last gavage dose on PND 4. On PND 22, blood was collected from these same F_1 dams and their weanlings for EB analysis 1 h after a 6-h inhalation exposure. The remainder of the F_2 generation was not directly exposed.

Limitations: This study was not specifically designed to investigate an endocrine mode of action. Although a negative result supports the conclusion that the response is not indicative of the hypothesis, a positive result could be due to several mechanisms.

Results included in WOE: There was no impairment of fertility or increased time to mating in the F_0 or F_1 animals.

1.13 Rank 2: Developmental Toxicity – Gestational length

[2] ***Faber and colleagues (2006)*** Four groups of Crl:CD(SD)IGS BR rats (30/sex/group for F_0 and 25/sex/group for F_1) were exposed to 0, 25, 100, and 500ppm EB for 6 h/day for at least 70 consecutive days before mating. Inhalation exposure for the F_0 and F_1 females continued throughout mating, gestation through gestation day (GD) 20, and lactation days (LD) 5–21. On LD 1–4, females received EB in corn oil via oral gavage at dose levels of 26, 90, and 342 mg/kg/day (divided into three equal doses, approximately 2 h apart). Pups were weaned on postnatal day (PND) 21 and exposure of the F_1 generation started on PND 22. Estimates of internal exposure were determined by measuring EB concentrations in blood collected from F_1 dams (4/group) and their culled pups 1 h after the last gavage dose on PND 4. On PND 22, blood was collected from these same F_1 dams and their weanlings for EB analysis 1 h after a 6-h inhalation exposure. The remainder of the F_2 generation was not directly exposed.

Limitations: This study was not specifically designed to investigate an endocrine mode of action. Although a negative result supports the conclusion that the response is not indicative of the hypothesis, a positive result could be due to several mechanisms.

Results included in WOE: No effects from ethylbenzene exposure of F_0 or F_1 animals were observed on reproductive performance parameters (mating and fertility indices, gestation lengths, former implantation sites and unaccounted sites).

1.14 Rank 2: Reproductive Toxicity – Implantations

[2] ***Faber and colleagues (2006)*** Four groups of Crl:CD(SD)IGS BR rats (30/sex/group for F_0 and 25/sex/group for F_1) were exposed to 0, 25, 100, and 500ppm EB for 6 h/day for at least 70 consecutive days before mating. Inhalation exposure for the F_0 and F_1 females continued throughout mating, gestation through gestation day (GD) 20, and lactation days (LD) 5–21. On LD 1–4, females received EB in corn oil via oral gavage at dose levels of 26, 90, and 342 mg/kg/day (divided into three equal doses, approximately 2 h apart). Pups were weaned on postnatal day (PND) 21 and exposure of the F_1 generation started on PND 22. Estimates of internal exposure were determined by measuring EB concentrations in blood collected from F_1 dams (4/group) and their culled pups 1 h after the last gavage dose on PND 4. On PND 22, blood was collected from these same F_1 dams and their weanlings for EB analysis 1 h after a 6-hr inhalation exposure. The remainder of the F_2 generation was not directly exposed.

Limitations: This study was not specifically designed to investigate an endocrine mode of action. Although a negative result supports the conclusion that the response is not indicative of the hypothesis, a positive result could be due to several mechanisms.

Results included in WOE: No effects from ethylbenzene exposure of F_0 or F_1 rats were observed on reproductive performance parameters (mating and fertility indices, gestation lengths, former implantation sites and unaccounted sites).

1.15 Rank 2: Reproductive Toxicity – Litter size

[2] ***Faber and colleagues (2006)*** Four groups of Crl:CD(SD)IGS BR rats (30/sex/group for F_0 and 25/sex/group for F_1) were exposed to 0, 25, 100, and 500ppm EB for 6 h/day for at least 70 consecutive days before mating. Inhalation exposure for the F_0 and F_1 females continued throughout mating, gestation through gestation day (GD) 20, and lactation days (LD) 5–21. On LD 1–4, females received EB in corn oil via oral gavage at dose levels of 26, 90, and 342 mg/kg/day (divided into three equal doses, approximately 2 h apart). Pups were weaned on postnatal day (PND) 21 and exposure of the F_1 generation started on PND 22. Estimates of internal exposure were determined by measuring EB concentrations in blood collected from F_1 dams (4/group) and their culled pups 1 h after the last gavage dose on PND 4. On PND 22, blood was collected from these same F_1 dams and their weanlings for EB analysis 1 h after a 6-h inhalation exposure. The remainder of the F_2 generation was not directly exposed.

Limitations: This study was not specifically designed to investigate an endocrine mode of action. Although a negative result supports the conclusion that the response is not indicative of the hypothesis, a positive result could be due to several mechanisms.

Results included in WOE: The mean number of F_1 and F_2 pups born, live litter size, percentage of males per litter at birth, and postnatal survival were unaffected by ethylbenzene exposure.

1.16 Rank 2: Reproductive Toxicity – Mating index

[2] ***Faber and colleagues (2006)*** Four groups of Crl:CD(SD)IGS BR rats (30/sex/group for F_0 and 25/sex/group for F_1) were exposed to 0, 25, 100, and 500ppm EB for 6 h/day for at least 70 consecutive days before mating. Inhalation exposure for the F_0 and F_1 females continued throughout mating, gestation through gestation day (GD) 20, and lactation days (LD) 5–21. On LD 1–4, females received EB in corn oil via oral gavage at dose levels of 26, 90, and 342 mg/kg/day (divided into three equal doses, approximately 2 h apart). Pups were weaned on postnatal day (PND) 21 and exposure of the F_1 generation started on PND 22. Estimates of internal exposure were determined by measuring EB concentrations in blood collected from F_1 dams (4/group) and their culled pups 1 h after the last gavage dose on PND 4. On PND 22, blood was collected from these same F_1 dams and their weanlings for EB analysis 1 h after a 6-h inhalation exposure. The remainder of the F_2 generation was not directly exposed.

Limitations: This study was not specifically designed to investigate an endocrine mode of action. Although a negative result supports the conclusion that the response is not indicative of the hypothesis, a positive result could be due to several mechanisms.

Results included in WOE: The male and female mating indices (%) were not different between any of the treatment animals and controls.

1.17 Rank 2: Reproductive Toxicity – Ovarian follicle count in offspring

[2] ***Faber and colleagues (2006)*** Four groups of Crl:CD(SD)IGS BR rats (30/sex/group for F_0 and 25/sex/group for F_1) were exposed to 0, 25, 100, and 500ppm EB for 6 h/day for at least 70

consecutive days before mating. Inhalation exposure for the F_0 and F_1 females continued throughout mating, gestation through gestation day (GD) 20, and lactation days (LD) 5–21. On LD 1–4, females received EB in corn oil via oral gavage at dose levels of 26, 90, and 342 mg/kg/day (divided into three equal doses, approximately 2 h apart). Pups were weaned on postnatal day (PND) 21 and exposure of the F_1 generation started on PND 22. Estimates of internal exposure were determined by measuring EB concentrations in blood collected from F_1 dams (4/group) and their culled pups 1 h after the last gavage dose on PND 4. On PND 22, blood was collected from these same F_1 dams and their weanlings for EB analysis 1 h after a 6-h inhalation exposure. The remainder of the F_2 generation was not directly exposed.

Limitations: This study was not specifically designed to investigate an endocrine mode of action. Although a negative result supports the conclusion that the response is not indicative of the hypothesis, a positive result could be due to several mechanisms.

Results included in WOE: In the F_1 females, the mean number of primordial follicles in the 500-ppm dose group was no significantly different from controls.

1.18 Rank 2: Reproductive Toxicity – Sperm count

[2] ***Faber and colleagues (2006)*** Four groups of Crl:CD(SD)IGS BR rats (30/sex/group for F_0 and 25/sex/group for F_1) were exposed to 0, 25, 100, and 500ppm EB for 6 h/day for at least 70 consecutive days before mating. Inhalation exposure for the F_0 and F_1 females continued throughout mating, gestation through gestation day (GD) 20, and lactation days (LD) 5–21. On LD 1–4, females received EB in corn oil via oral gavage at dose levels of 26, 90, and 342 mg/kg/day (divided into three equal doses, approximately 2 h apart). Pups were weaned on postnatal day (PND) 21 and exposure of the F_1 generation started on PND 22. Estimates of internal exposure were determined by measuring EB concentrations in blood collected from F_1 dams (4/group) and their culled pups 1 h after the last gavage dose on PND 4. On PND 22, blood was collected from these same F_1 dams and their weanlings for EB analysis 1 h after a 6-h inhalation exposure. The remainder of the F_2 generation was not directly exposed.

Limitations: This study was not specifically designed to investigate an endocrine mode of action. Although a negative result supports the conclusion that the response is not indicative of the hypothesis, a positive result could be due to several mechanisms.

Results included in WOE: The mean sperm number (millions/g tissue) in the left cauda epididymis for F_0 and F_1 males were not different between any treatment group and controls.

1.19 Rank 2: Reproductive Toxicity – Time to mating

[2] ***Faber and colleagues (2006)*** Four groups of Crl:CD(SD)IGS BR rats (30/sex/group for F_0 and 25/sex/group for F1) were exposed to 0, 25, 100, and 500ppm EB for 6 h/day for at least 70 consecutive days before mating. Inhalation exposure for the F_0 and F_1 females continued throughout mating, gestation through gestation day (GD) 20, and lactation days (LD) 5–21. On LD 1–4, females received EB in corn oil via oral gavage at dose levels of 26, 90, and 342 mg/kg/day (divided into three equal doses, approximately 2 h apart). Pups were weaned on postnatal day (PND) 21 and exposure of the F_1 generation started on PND 22.

Estimates of internal exposure were determined by measuring EB concentrations in blood collected from F_1 dams (4/group) and their culled pups 1 h after the last gavage dose on PND 4. On PND 22, blood was collected from these same F_1 dams and their weanlings for EB analysis 1 h after a 6-hr inhalation exposure. The remainder of the F_2 generation was not directly exposed.

Limitations: This study was not specifically designed to investigate an endocrine mode of action. Although a negative result supports the conclusion that the response is not indicative of the hypothesis, a positive result could be due to several mechanisms.

Results included in WOE: There was no impairment of fertility or increased time to mating in the F_0 or F_1 animals.

1.20 Rank 2: Reproductive Toxicity – Time to vaginal patency

[2] ***Faber and colleagues (2006)*** Four groups of Crl:CD(SD)IGS BR rats (30/sex/group for F_0 and 25/sex/group for F_1) were exposed to 0, 25, 100, and 500ppm EB for 6 h/day for at least 70 consecutive days before mating. Inhalation exposure for the F_0 and F_1 females continued throughout mating, gestation through gestation day (GD) 20, and lactation days (LD) 5–21. On LD 1–4, females received EB in corn oil via oral gavage at dose levels of 26, 90, and 342 mg/kg/day (divided into three equal doses, approximately 2 h apart). Pups were weaned on postnatal day (PND) 21 and exposure of the F_1 generation started on PND 22. Estimates of internal exposure were determined by measuring EB concentrations in blood collected from F_1 dams (4/group) and their culled pups 1 h after the last gavage dose on PND 4. On PND 22, blood was collected from these same F_1 dams and their weanlings for EB analysis 1 h after a 6-h inhalation exposure. The remainder of the F_2 generation was not directly exposed.

Limitations: This study was not specifically designed to investigate an endocrine mode of action. Although a negative result supports the conclusion that the response is not indicative of the hypothesis, a positive result could be due to several mechanisms.

Results included in WOE: The mean age of acquisition of vaginal patency for all exposed groups (25, 100 and 500 ppm ethylbenzene) was statistically significantly lower than the mean for the concurrent control group value in F_1 female offspring; similar differences were not observed in the F_2 female pups. The authors felt these differences were not biologically important because the mean values were comparable to the historical control mean value.

1.21 Rank 3: Repeat Dose Toxicity – Gross pathology

[3] ***Mellert and colleagues (2007)*** Ethylbenzene was administered to groups of male and female Wistar rats by gavage for 4 (n = 5/ dose/sex) and 13 weeks (n = 10/dose/sex) (OECD 408) at doses of 0 (vehicle control), 75, 250, and 750 mg/kg bw/day, administered am/pm as half doses.

Limitations: This study was not specifically designed to investigate an endocrine mode of action. Although a negative result supports the conclusion that the response is not indicative of the hypothesis, a positive result could be due to several mechanisms.

Results included in WOE: Gross pathological evaluation of the epididymis, mammary glands, ovaries, prostate, seminal vesicles, testes, thyroid, uterus, and vagina did not identify any adverse effects.

2. Estrogen Antagonist Hypothesis

2.1 Rank 2: Repeat Dose Toxicity – Epididymis histopathology

[1] ***NTP (National Toxicology Program). (1999)*** Groups of 50 male and 50 female Fischer rats and B6C3F1 mice were exposed to ethylbenzene by inhalation at 0, 75, 250, and 750 ppm 6 h per day, 5 days per week, for 104 weeks (rats) and for 103 week (mice).

Limitations: This study was not specifically designed to investigate an endocrine mode of action. Although a negative result supports the conclusion that the response is not indicative of the hypothesis, a positive result could be due to several mechanisms.

Results included in WOE: There were no significant histological differences between the chamber control animals and treatment group animals, in either specie, for uterine, vaginal or ovarian tissues in females or for testes, prostate, epididymides or seminal vesicles for males.

[3] ***Mellert and colleagues (2007)*** Ethylbenzene was administered to groups of male and female Wistar rats by gavage for 4 (*n* = 5/ dose/sex) and 13 weeks (*n* = 10/dose/sex) (OECD 408) at doses of 0 (vehicle control), 75, 250, and 750 mg/kg bw/day, administered am/pm as half doses.

Limitations: This study was not specifically designed to investigate an endocrine mode of action. Although a negative result supports the conclusion that the response is not indicative of the hypothesis, a positive result could be due to several mechanisms.

Results included in WOE: Histopathological evaluation of the epididymis, mammary glands, ovaries, prostate, seminal vesicles, testes, thyroid, uterus, and vagina did not identify any adverse effects.

[9] ***NTP (National Toxicology Program). Toxicity studies of ethylbenzene (CAS No. 100-41-4) in F344/N rats and B6C3F1 mice (inhalation studies) (1992)*** Inhalation toxicity of ethylbenzene was studied by exposing groups of seven-week-old F344/N rats and B6C3Fi mice of each sex to ethylbenzene vapor at chamber concentrations of 0, 100, 250, 500, 750, or 1000 ppm, 6 h per day, 5 days per week for 13 weeks.

Limitations: This study was not specifically designed to investigate an endocrine mode of action. Although a negative result supports the conclusion that the response is not indicative of the hypothesis, a positive result could be due to several mechanisms.

Results included in WOE: There were no histopathologic lesions and no chemically related histopathologic changes identified in the epididymides of mice or rats compared with controls.

[10] ***Cragg and colleagues (1989)*** Mice, rats and rabbits (five/sex/group) were exposed by inhalation to ethylbenzene vapors for 6 h/day, 5 days/week for 4 weeks (20 exposures). Rats and mice received 0, 99, 382 or 782 ppm EB while rabbits received 0, 382, 782 or 1610 ppm.

Limitations: This study was not specifically designed to investigate an endocrine mode of action. Although a negative result supports the conclusion that the response is not indicative of the hypothesis, a positive result could be due to several mechanisms.

Results included in WOE: The gonads (ovaries or testes with epididymides) and thyroids of high-exposure and controls animals of all species were subjected to histopathological examination. There were no gross or microscopic changes attributable to ethylbenzene in any of these tissues.

2.2 Rank 2: Repeat Dose Toxicity – Ovary histopathology

[1] ***NTP (National Toxicology Program). (1999)*** Groups of 50 male and 50 female Fischer rats and B6C3F1 mice were exposed to ethylbenzene by inhalation at 0, 75, 250, and 750 ppm 6 h per day, 5 days per week, for 104 weeks (rats) and for 103 week (mice).

Limitations: This study was not specifically designed to investigate an endocrine mode of action. Although a negative result supports the conclusion that the response is not indicative of the hypothesis, a positive result could be due to several mechanisms.

Results included in WOE: There were no significant histological differences between the chamber control animals and treatment group animals, in either specie, for uterine, vaginal or ovarian tissues in females or for testes, prostate, epididymides or seminal vesicles for males.

[3] ***Mellert and colleagues (2007)*** Ethylbenzene was administered to groups of male and female Wistar rats by gavage for 4 (n = 5/ dose/sex) and 13 weeks (n = 10/dose/sex) (OECD 408) at doses of 0 (vehicle control), 75, 250, and 750 mg/kg bw/day, administered am/pm as half doses.

Limitations: This study was not specifically designed to investigate an endocrine mode of action. Although a negative result supports the conclusion that the response is not indicative of the hypothesis, a positive result could be due to several mechanisms.

Results included in WOE: Histopathological evaluation of the epididymis, mammary glands, ovaries, prostate, seminal vesicles, testes, thyroid, uterus, and vagina did not identify any adverse effects.

[9] ***NTP (National Toxicology Program). Toxicity studies of ethylbenzene (CAS No. 100-41-4) in F344/N rats and B6C3F1 mice (inhalation studies) (1992)*** Inhalation toxicity of ethylbenzene was studied by exposing groups of seven-week-old F344/N rats and B6C3Fi mice of each sex to ethylbenzene vapor at chamber concentrations of 0, 100, 250, 500, 750, or 1000 ppm, 6 h per day, 5 days per week for 13 weeks.

Limitations: This study was not specifically designed to investigate an endocrine mode of action. Although a negative result supports the conclusion that the response is not indicative of the hypothesis, a positive result could be due to several mechanisms.

Results included in WOE: There were no histopathologic lesions and no chemically related histopathologic changes identified in the ovaries of mice or rats compared with controls.

[10] Cragg and colleagues (1989) Mice, rats and rabbits (five/sex/group) were exposed by inhalation to ethylbenzene vapors for 6 h/day, 5 days/week for 4 weeks (20 exposures). Rats and mice received 0, 99, 382 or 782 ppm EB while rabbits received 0, 382, 782 or 1610 ppm.

Limitations: This study was not specifically designed to investigate an endocrine mode of action. Although a negative result supports the conclusion that the response is not indicative of the hypothesis, a positive result could be due to several mechanisms.

Results included in WOE: The gonads (ovaries or testes with epididymides) and thyroids of high-exposure and controls animals of all species were subjected to histopathological examination. There were no gross or microscopic changes attributable to ethylbenzene in any of these tissues.

2.3 Rank 2: Repeat Dose Toxicity – Prostate histopathology

[1] NTP ***(National Toxicology Program). (1999)*** Groups of 50 male and 50 female Fischer rats and B6C3F1 mice were exposed to ethylbenzene by inhalation at 0, 75, 250, and 750 ppm 6 h per day, 5 days per week, for 104 weeks (rats) and for 103 week (mice).

Limitations: This study was not specifically designed to investigate an endocrine mode of action. Although a negative result supports the conclusion that the response is not indicative of the hypothesis, a positive result could be due to several mechanisms.

Results included in WOE: There were no significant histological differences between the chamber control animals and treatment group animals, in either specie, for uterine, vaginal or ovarian tissues in females or for testes, prostate, epididymides or seminal vesicles for males.

[3] ***Mellert and colleagues (2007)*** Ethylbenzene was administered to groups of male and female Wistar rats by gavage for 4 (n = 5/ dose/sex) and 13 weeks (n = 10/dose/sex) (OECD 408) at doses of 0 (vehicle control), 75, 250, and 750 mg/kg bw/day, administered am/pm as half doses.

Limitations: This study was not specifically designed to investigate an endocrine mode of action. Although a negative result supports the conclusion that the response is not indicative of the hypothesis, a positive result could be due to several mechanisms.

Results included in WOE: Histopathological evaluation of the epididymis, mammary glands, ovaries, prostate, seminal vesicles, testes, thyroid, uterus, and vagina did not identify any adverse effects.

[9] ***NTP (National Toxicology Program). Toxicity studies of ethylbenzene (CAS No. 100-41-4) in F344/N rats and B6C3F1 mice (inhalation studies) (1992)*** Inhalation toxicity of ethylbenzene was studied by exposing groups of seven-week-old F344/N rats and B6C3Fi mice of each sex to ethylbenzene vapor at chamber concentrations of 0, 100, 250, 500, 750, or 1000 ppm, 6 h per day, 5 days per week for 13 weeks.

Limitations: This study was not specifically designed to investigate an endocrine mode of action. Although a negative result supports the conclusion that the response is not indicative of the hypothesis, a positive result could be due to several mechanisms.

Results included in WOE: There were no histopathologic lesions and no chemically related histopathologic changes identified in the prostates of mice or rats compared with controls.

2.4 Rank 2: Repeat Dose Toxicity – Seminal vesicle histopathology

[1] ***NTP (National Toxicology Program). (1999)*** Groups of 50 male and 50 female Fischer rats and B6C3F1 mice were exposed to ethylbenzene by inhalation at 0, 75, 250, and 750 ppm 6 h per day, 5 days per week, for 104 weeks (rats) and for 103 week (mice).

Limitations: This study was not specifically designed to investigate an endocrine mode of action. Although a negative result supports the conclusion that the response is not indicative of the hypothesis, a positive result could be due to several mechanisms.

Results included in WOE: There were no significant histological differences between the chamber control animals and treatment group animals, in either specie, for uterine, vaginal or ovarian tissues in females or for testes, prostate, epididymides or seminal vesicles for males.

[3] ***Mellert and colleagues (2007)*** Ethylbenzene was administered to groups of male and female Wistar rats by gavage for 4 (*n* = 5/ dose/sex) and 13 weeks (*n* = 10/dose/sex) (OECD 408) at doses of 0 (vehicle control), 75, 250, and 750 mg/kg bw/day, administered am/pm as half doses.

Limitations: This study was not specifically designed to investigate an endocrine mode of action. Although a negative result supports the conclusion that the response is not indicative of the hypothesis, a positive result could be due to several mechanisms.

Results included in WOE: Histopathological evaluation of the epididymis, mammary glands, ovaries, prostate, seminal vesicles, testes, thyroid, uterus, and vagina did not identify any adverse effects.

[9] ***NTP (National Toxicology Program). Toxicity studies of ethylbenzene (CAS No. 100-41-4) in F344/N rats and B6C3F1 mice (inhalation studies) (1992)*** Inhalation toxicity of ethylbenzene was studied by exposing groups of seven-week-old F344/N rats and B6C3Fi mice of each sex to ethylbenzene vapor at chamber concentrations of 0, 100, 250, 500, 750, or 1000 ppm, 6 hper day, 5 days per week for 13 weeks.

Limitations: This study was not specifically designed to investigate an endocrine mode of action. Although a negative result supports the conclusion that the response is not indicative of the hypothesis, a positive result could be due to several mechanisms.

Results included in WOE: There were no histopathologic lesions and no chemically related histopathologic changes identified in the seminal vesicles of mice or rats compared with controls.

2.5 Rank 2: Repeat dose toxicity – Testis histopathology (atrophy)

[1] ***NTP (National Toxicology Program). (1999)*** Groups of 50 male and 50 female Fischer rats and B6C3F1 mice were exposed to ethylbenzene by inhalation at 0, 75, 250, and 750 ppm 6 h per day, 5 days per week, for 104 weeks (rats) and for 103 week (mice).

Limitations: This study was not specifically designed to investigate an endocrine mode of action. Although a negative result supports the conclusion that the response is not indicative of the hypothesis, a positive result could be due to several mechanisms.

Results included in WOE: There were no significant histological differences between the chamber control animals and treatment group animals, in either specie, for uterine, vaginal or ovarian tissues in females or for testes, prostate, epididymides or seminal vesicles for males.

[3] ***Mellert and colleagues (2007)*** Ethylbenzene was administered to groups of male and female Wistar rats by gavage for 4 (*n* = 5/ dose/sex) and 13 weeks (*n* = 10/dose/sex) (OECD 408) at doses of 0 (vehicle control), 75, 250, and 750 mg/kg bw/day, administered am/pm as half doses.

Limitations: This study was not specifically designed to investigate an endocrine mode of action. Although a negative result supports the conclusion that the response is not indicative of the hypothesis, a positive result could be due to several mechanisms.

Results included in WOE: Histopathological evaluation of the epididymis, mammary glands, ovaries, prostate, seminal vesicles, testes, thyroid, uterus, and vagina did not identify any adverse effects.

[9] ***NTP (National Toxicology Program). Toxicity studies of ethylbenzene (CAS No. 100-41-4) in F344/N rats and B6C3F1 mice (inhalation studies) (1992)*** Inhalation toxicity of ethylbenzene was studied by exposing groups of seven-week-old F344/N rats and B6C3Fi mice of each sex to ethylbenzene vapor at chamber concentrations of 0, 100, 250, 500, 750, or 1000 ppm, 6 h per day, 5 days per week for 13 weeks.

Limitations: This study was not specifically designed to investigate an endocrine mode of action. Although a negative result supports the conclusion that the response is not indicative of the hypothesis, a positive result could be due to several mechanisms.

Results included in WOE: There were no histopathologic lesions and no chemically related histopathologic changes identified in testes of mice or rats compared with controls.

[10] Cragg and colleagues (1989) Mice, rats and rabbits (five/sex/group) were exposed by inhalation to ethylbenzene vapors for 6 h/day, 5 days/week for 4 weeks (20 exposures). Rats and mice received 0, 99, 382 or 782 ppm EB while rabbits received 0, 382, 782 or 1610 ppm.

Limitations: This study was not specifically designed to investigate an endocrine mode of action. Although a negative result supports the conclusion that the response is not indicative of the hypothesis, a positive result could be due to several mechanisms.

Results included in WOE: The gonads (ovaries or testes with epididymides) and thyroids of high-exposure and controls animals of all species were subjected to histopathological examination. There were no gross or microscopic changes attributable to ethylbenzene in any of these tissues.

2.6 Rank 2: Repeat Dose Toxicity – Testis weight

[9] ***NTP (National Toxicology Program). Toxicity studies of ethylbenzene (CAS No. 100-41-4) in F344/N rats and B6C3F1 mice (inhalation studies) (1992)*** Inhalation toxicity of ethylbenzene was studied by exposing groups of seven-week-old F344/N rats and B6C3Fi mice of each sex to ethylbenzene vapor at chamber concentrations of 0, 100, 250, 500, 750, or 1000 ppm, 6 h per day, 5 days per week for 13 weeks.

Limitations: This study was not specifically designed to investigate an endocrine mode of action. Although a negative result supports the conclusion that the response is not indicative of the hypothesis, a positive result could be due to several mechanisms.

Results included in WOE: Weights of testes in mice and rats were not affected by ethylbenzene.

2.7 Rank 2: Developmental Toxicity – Corpora lutea

[5] ***Saillenfait and colleagues (2003)*** The developmental toxicity of ethylbenzene was studied in Sprague–Dawley rats after inhalation exposure. Animals were exposed to ethylbenzene at 100, 500, 1000 or 2000 ppm, for 6 h/day, during days 6–20 of gestation.

Limitations: This study was not specifically designed to investigate an endocrine mode of action. Although a negative result supports the conclusion that the response is not indicative of the hypothesis, a positive result could be due to several mechanisms.

Results included in WOE: The mean number of corpora lutea per dam did not differ between dams in any of the treatment groups and control dams.

[13] ***Andrew et al., (1981); Hardin et al. (1981)*** Groups of 29-33 Female Wistar rats and New Zealand White rabbits were exposed to 0, 100, or 1000 ppm ethylbenzene for 7 h/day, 5 days/week for 3 weeks, then mated with unexposed males. Pregnant females were further exposed to 0, 100, or 1000 ppm 7 h/day through Gestational Day 19 (rats) and 24 (rabbits).

Limitations: This study was not specifically designed to investigate an endocrine mode of action. Although a negative result supports the conclusion that the response is not indicative of the hypothesis, a positive result could be due to several mechanisms.

Results included in the WOE: The number of Corpora Lutea was unchanged by exposure to 100 and 1,000 ppm ethylbenzene relative to controls.

2.8 Rank 2: Reproductive Toxicity – Estrous cyclicity

[2] ***Faber and colleagues (2006)*** Four groups of Crl:CD(SD)IGS BR rats (30/sex/group for F_0 and 25/sex/group for F_1) were exposed to 0, 25, 100, and 500ppm EB for 6 h/day for at least 70 consecutive days before mating. Inhalation exposure for the F_0 and F_1 females continued throughout mating, gestation through gestation day (GD) 20, and lactation days (LD) 5–21. On LD 1–4, females received EB in corn oil via oral gavage at dose levels of 26, 90, and 342 mg/kg/day (divided into three equal doses, approximately 2 h apart). Pups were weaned on postnatal day (PND) 21 and exposure of the F_1 generation started on PND 22. Estimates of internal exposure were determined by measuring EB concentrations in blood collected from F_1 dams (4/group) and their culled pups 1 h after the last gavage dose on PND 4. On PND 22, blood was collected from these same F_1 dams and their weanlings for EB analysis 1 h after a 6-h inhalation exposure. The remainder of the F_2 generation was not directly exposed.

Limitations: This study was not specifically designed to investigate an endocrine mode of action. Although a negative result supports the conclusion that the response is not indicative of the hypothesis, a positive result could be due to several mechanisms.

Results included in WOE: The mean estrous cycle length (4.0 ± 0.3 days) was significantly reduced for the F_0, 500ppm group when compared to the F_0 control group value (4.4 ± 0.8 days). However, the authors felt this difference was not biologically important because all females in this group were cycling normally and this strain of rat normally exhibits 4-5 day estrous cycles. Mean estrous cycle length did not differ between control and experimental F_1 offspring.

2.9 Rank 2: Reproductive Toxicity – Fertility

[2] ***Faber and colleagues (2006)*** Four groups of Crl:CD(SD)IGS BR rats (30/sex/group for F_0 and 25/sex/group for F_1) were exposed to 0, 25, 100, and 500ppm EB for 6 h/day for at least 70 consecutive days before mating. Inhalation exposure for the F_0 and F_1 females continued throughout mating, gestation through gestation day (GD) 20, and lactation days (LD) 5–21. On LD 1–4, females received EB in corn oil via oral gavage at dose levels of 26, 90, and 342 mg/kg/day (divided into three equal doses, approximately 2 h apart). Pups were weaned on postnatal day (PND) 21 and exposure of the F_1 generation started on PND 22. Estimates of internal exposure were determined by measuring EB concentrations in blood collected from F_1 dams (4/group) and their culled pups 1 h after the last gavage dose on PND 4. On PND 22, blood was collected from these same F_1 dams and their weanlings for EB analysis 1 h after a 6-h inhalation exposure. The remainder of the F_2 generation was not directly exposed.

Limitations: This study was not specifically designed to investigate an endocrine mode of action. Although a negative result supports the conclusion that the response is not indicative of the hypothesis, a positive result could be due to several mechanisms.

Results included in WOE: There was no impairment of fertility or increased time to mating in the F_0 or F_1 animals.

2.10 Rank 2: Reproductive Toxicity – Litter size

[2] ***Faber and colleagues (2006)*** Four groups of Crl:CD(SD)IGS BR rats (30/sex/group for F_0 and 25/sex/group for F_1) were exposed to 0, 25, 100, and 500ppm EB for 6 h/day for at least 70 consecutive days before mating. Inhalation exposure for the F_0 and F_1 females continued throughout mating, gestation through gestation day (GD) 20, and lactation days (LD) 5–21. On LD 1–4, females received EB in corn oil via oral gavage at dose levels of 26, 90, and 342 mg/kg/day (divided into three equal doses, approximately 2 h apart). Pups were weaned on postnatal day (PND) 21 and exposure of the F_1 generation started on PND 22. Estimates of internal exposure were determined by measuring EB concentrations in blood collected from F_1 dams (4/group) and their culled pups 1 h after the last gavage dose on PND 4. On PND 22, blood was collected from these same F_1 dams and their weanlings for EB analysis 1 h after a 6-h inhalation exposure. The remainder of the F_2 generation was not directly exposed.

Limitations: This study was not specifically designed to investigate an endocrine mode of action. Although a negative result supports the conclusion that the response is not indicative of the hypothesis, a positive result could be due to several mechanisms.

Results included in WOE: The mean number of F_1 and F_2 pups born, live litter size, percentage of males per litter at birth, and postnatal survival were unaffected by ethylbenzene exposure.

2.11 Rank 2: Reproductive Toxicity – Sperm count

[2] ***Faber and colleagues (2006)*** Four groups of Crl:CD(SD)IGS BR rats (30/sex/group for F_0 and 25/sex/group for F_1) were exposed to 0, 25, 100, and 500ppm EB for 6 h/day for at least 70 consecutive days before mating. Inhalation exposure for the F_0 and F_1 females continued throughout mating, gestation through gestation day (GD) 20, and lactation days (LD) 5–21. On LD 1–4, females received EB in corn oil via oral gavage at dose levels of 26, 90, and 342 mg/kg/day (divided into three equal doses, approximately 2 h apart). Pups were weaned on postnatal day (PND) 21 and exposure of

the F_1 generation started on PND 22. Estimates of internal exposure were determined by measuring EB concentrations in blood collected from F_1 dams (4/group) and their culled pups 1 h after the last gavage dose on PND 4. On PND 22, blood was collected from these same F_1 dams and their weanlings for EB analysis 1 h after a 6-h inhalation exposure. The remainder of the F_2 generation was not directly exposed.

Limitations: This study was not specifically designed to investigate an endocrine mode of action. Although a negative result supports the conclusion that the response is not indicative of the hypothesis, a positive result could be due to several mechanisms.

Results included in WOE: The mean sperm number (millions/g tissue) in the left cauda epididymis for F_0 and F_1 males were not different between any treatment group and controls.

2.12 Rank 2: Reproductive Toxicity – Time to mating

[2] ***Faber and colleagues (2006)*** Four groups of Crl:CD(SD)IGS BR rats (30/sex/group for F_0 and 25/sex/group for F_1) were exposed to 0, 25, 100, and 500ppm EB for 6 h/day for at least 70 consecutive days before mating. Inhalation exposure for the F_0 and F_1 females continued throughout mating, gestation through gestation day (GD) 20, and lactation days (LD) 5–21. On LD 1–4, females received EB in corn oil via oral gavage at dose levels of 26, 90, and 342 mg/kg/day (divided into three equal doses, approximately 2 h apart). Pups were weaned on postnatal day (PND) 21 and exposure of the F_1 generation started on PND 22. Estimates of internal exposure were determined by measuring EB concentrations in blood collected from F_1 dams (4/group) and their culled pups 1 h after the last gavage dose on PND 4. On PND 22, blood was collected from these same F_1 dams and their weanlings for EB analysis 1 h after a 6-h inhalation exposure. The remainder of the F_2 generation was not directly exposed.

Limitations: This study was not specifically designed to investigate an endocrine mode of action. Although a negative result supports the conclusion that the response is not indicative of the hypothesis, a positive result could be due to several mechanisms.

Results included in WOE: There was no impairment of fertility or increased time to mating in the F_0 or F_1 animals.

2.13 Rank 2: Reproductive Toxicity – Time to vaginal patency in offspring

[2] ***Faber and colleagues (2006)*** Four groups of Crl:CD(SD)IGS BR rats (30/sex/group for F_0 and 25/sex/group for F_1) were exposed to 0, 25, 100, and 500ppm EB for 6 h/day for at least 70 consecutive days before mating. Inhalation exposure for the F_0 and F_1 females continued throughout mating, gestation through gestation day (GD) 20, and lactation days (LD) 5–21. On LD 1–4, females received EB in corn oil via oral gavage at dose levels of 26, 90, and 342 mg/kg/day (divided into three equal doses, approximately 2 h apart). Pups were weaned on postnatal day (PND) 21 and exposure of the F_1 generation started on PND 22. Estimates of internal exposure were determined by measuring EB concentrations in blood collected from F_1 dams (4/group) and their culled pups 1 h after the last gavage dose on PND 4. On PND 22, blood was collected from these same F_1 dams and their weanlings for EB analysis 1 h after a 6-h inhalation exposure. The remainder of the F_2 generation was not directly exposed.

Limitations: This study was not specifically designed to investigate an endocrine mode of action. Although a negative result supports the conclusion that the response is not indicative of the hypothesis, a positive result could be due to several mechanisms.

Results included in WOE: The mean age of acquisition of vaginal patency for all exposed groups (25, 100 and 500 ppm ethylbenzene) was statistically significantly lower than the mean for the concurrent control group value in F_1 female offspring; similar differences were not observed in the F_2 female pups. The authors felt these differences were not biologically important because the mean values were comparable to the historical control mean value.

2.14 Rank 3: Repeat Dose Toxicity – Gross Pathology

[3] ***Mellert and colleagues (2007)*** Ethylbenzene was administered to groups of male and female Wistar rats by gavage for 4 (*n* = 5/ dose/sex) and 13 weeks (*n* = 10/dose/sex) (OECD 408) at doses of 0 (vehicle control), 75, 250, and 750 mg/kg bw/day, administered am/pm as half doses.

Limitations: This study was not specifically designed to investigate an endocrine mode of action. Although a negative result supports the conclusion that the response is not indicative of the hypothesis, a positive result could be due to several mechanisms.

Results included in WOE: Gross pathological evaluation of the epididymis, mammary glands, ovaries, prostate, seminal vesicles, testes, thyroid, uterus, and vagina did not identify any adverse effects.

3. Androgen Agonist Hypothesis

3.1 Rank 2: Repeat Dose Toxicity – Ovary histopathology

[1] ***NTP (National Toxicology Program). (1999)*** Groups of 50 male and 50 female Fischer rats and B6C3F1 mice were exposed to ethylbenzene by inhalation at 0, 75, 250, and 750 ppm 6 h per day, 5 days per week, for 104 weeks (rats) and for 103 week (mice).

Limitations: This study was not specifically designed to investigate an endocrine mode of action. Although a negative result supports the conclusion that the response is not indicative of the hypothesis, a positive result could be due to several mechanisms.

Results included in WOE: There were no significant histological differences between the chamber control animals and treatment group animals, in either specie, for uterine, vaginal or ovarian tissues in females or for testes, prostate, epididymides or seminal vesicles for males.

[3] ***Mellert and colleagues (2007)*** Ethylbenzene was administered to groups of male and female Wistar rats by gavage for 4 (*n* = 5/ dose/sex) and 13 weeks (*n* = 10/dose/sex) (OECD 408) at doses of 0 (vehicle control), 75, 250, and 750 mg/kg bw/day, administered am/pm as half doses.

Limitations: This study was not specifically designed to investigate an endocrine mode of action. Although a negative result supports the conclusion that the response is not indicative of the hypothesis, a positive result could be due to several mechanisms.

Results included in WOE: Histopathological evaluation of the epididymis, mammary glands, ovaries, prostate, seminal vesicles, testes, thyroid, uterus, and vagina did not identify any adverse effects.

[9] ***NTP (National Toxicology Program). Toxicity studies of ethylbenzene (CAS No. 100-41-4) in F344/N rats and B6C3F1 mice (inhalation studies) (1992)*** Inhalation toxicity of ethylbenzene was studied by exposing groups of seven-week-old F344/N rats and B6C3Fi mice of each sex to ethylbenzene vapor at chamber concentrations of 0, 100, 250, 500, 750, or 1000 ppm, 6 h per day, 5 days per week for 13 weeks.

Limitations: This study was not specifically designed to investigate an endocrine mode of action. Although a negative result supports the conclusion that the response is not indicative of the hypothesis, a positive result could be due to several mechanisms.

Results included in WOE: There were no histopathologic lesions and no chemically related histopathologic changes identified in the ovaries of mice or rats compared with controls.

[10] Cragg and colleagues (1989) Mice, rats and rabbits (five/sex/group) were exposed by inhalation to ethylbenzene vapors for 6 h/day, 5 days/week for 4 weeks (20 exposures). Rats and mice received 0, 99, 382 or 782 ppm EB while rabbits received 0, 382, 782 or 1610 ppm.

Limitations: This study was not specifically designed to investigate an endocrine mode of action. Although a negative result supports the conclusion that the response is not indicative of the hypothesis, a positive result could be due to several mechanisms.

Results included in WOE: The gonads (ovaries or testes with epididymides) and thyroids of high-exposure and controls animals of all species were subjected to histopathological examination. There were no gross or microscopic changes attributable to ethylbenzene in any of these tissues.

3.2 Rank 2: Repeat Dose Toxicity – Sperm count

[9] ***NTP (National Toxicology Program). Toxicity studies of ethylbenzene (CAS No. 100-41-4) in F344/N rats and B6C3F1 mice (inhalation studies) (1992)*** Inhalation toxicity of ethylbenzene was studied by exposing groups of seven-week-old F344/N rats and B6C3Fi mice of each sex to ethylbenzene vapor at chamber concentrations of 0, 100, 250, 500, 750, or 1000 ppm, 6 h per day, 5 days per week for 13 weeks.

Limitations: This study was not specifically designed to investigate an endocrine mode of action. Although a negative result supports the conclusion that the response is not indicative of the hypothesis, a positive result could be due to several mechanisms.

Results included in WOE: Sperm counts (sperm count/gram testis) were not significantly different between control and ethylbenzene-exposed mice or rats.

3.3 Rank 2: Repeat dose toxicity – Testis histopathology (atrophy)

[1] ***NTP (National Toxicology Program). (1999)*** Groups of 50 male and 50 female Fischer rats and B6C3F1 mice were exposed to ethylbenzene by inhalation at 0, 75, 250, and 750 ppm 6 h per day, 5 days per week, for 104 weeks (rats) and for 103 week (mice).

Limitations: This study was not specifically designed to investigate an endocrine mode of action. Although a negative result supports the conclusion that the response is not indicative of the hypothesis, a positive result could be due to several mechanisms.

Results included in WOE: There were no significant histological differences between the chamber control animals and treatment group animals, in either specie, for uterine, vaginal or ovarian tissues in females or for testes, prostate, epididymides or seminal vesicles for males.

[3] ***Mellert and colleagues (2007)*** Ethylbenzene was administered to groups of male and female Wistar rats by gavage for 4 (*n* = 5/ dose/sex) and 13 weeks (*n* = 10/dose/sex) (OECD 408) at doses of 0 (vehicle control), 75, 250, and 750 mg/kg bw/day, administered am/pm as half doses.

Limitations: This study was not specifically designed to investigate an endocrine mode of action. Although a negative result supports the conclusion that the response is not indicative of the hypothesis, a positive result could be due to several mechanisms.

Results included in WOE: Histopathological evaluation of the epididymis, mammary glands, ovaries, prostate, seminal vesicles, testes, thyroid, uterus, and vagina did not identify any adverse effects.

[9] ***NTP (National Toxicology Program). Toxicity studies of ethylbenzene (CAS No. 100-41-4) in F344/N rats and B6C3F1 mice (inhalation studies) (1992)*** Inhalation toxicity of ethylbenzene was studied by exposing groups of seven-week-old F344/N rats and B6C3Fi mice of each sex to ethylbenzene vapor at chamber concentrations of 0, 100, 250, 500, 750, or 1000 ppm, 6 h per day, 5 days per week for 13 weeks.

Limitations: This study was not specifically designed to investigate an endocrine mode of action. Although a negative result supports the conclusion that the response is not indicative of the hypothesis, a positive result could be due to several mechanisms.

Results included in WOE: There were no histopathologic lesions and no chemically related histopathologic changes identified in testes of mice or rats compared with controls.

[10] Cragg and colleagues (1989) Mice, rats and rabbits (five/sex/group) were exposed by inhalation to ethylbenzene vapors for 6 h/day, 5 days/week for 4 weeks (20 exposures). Rats and mice received 0, 99, 382 or 782 ppm EB while rabbits received 0, 382, 782 or 1610 ppm.

Limitations: This study was not specifically designed to investigate an endocrine mode of action. Although a negative result supports the conclusion that the response is not indicative of the hypothesis, a positive result could be due to several mechanisms.

Results included in WOE: The gonads (ovaries or testes with epididymides) and thyroids of high-exposure and controls animals of all species were subjected to histopathological examination. There were no gross or microscopic changes attributable to ethylbenzene in any of these tissues.

3.4 Rank 2: Repeat Dose Toxicity – Testis weight

[9] ***NTP (National Toxicology Program). Toxicity studies of ethylbenzene (CAS No. 100-41-4) in F344/N rats and B6C3F1 mice (inhalation studies) (1992)*** Inhalation toxicity of ethylbenzene was studied by exposing groups of seven-week-old F344/N rats and B6C3Fi mice of each sex to ethylbenzene vapor at chamber concentrations of 0, 100, 250, 500, 750, or 1000 ppm, 6 h per day, 5 days per week for 13 weeks.

Limitations: This study was not specifically designed to investigate an endocrine mode of action.

Although a negative result supports the conclusion that the response is not indicative of the hypothesis, a positive result could be due to several mechanisms.

Results included in WOE: Weights of testes in mice and rats were not affected by ethylbenzene.

3.5 Rank 2: Developmental Toxicity – Implantations

[4] ***Saillenfait and colleagues (2007)*** The combined effects of EB and BA were investigated. Groups of 18 bred rats (15– 18 pregnant) were exposed to vapors of EB or BA, separately or in combination, 6 h day−1, on days 6–20 of gestation. There were nine experimental groups: Control; 250 or 1000 ppm EB; 500 or 1500 ppm BA or mixtures of 250 ppm EB + 500 ppm BA, 250 ppm EB + 1500 ppm BA, 1000 ppm EB + 500 ppm BA, or 1000 ppm EB + 1500 ppm BA

Limitations: This study was not specifically designed to investigate an endocrine mode of action. Although a negative result supports the conclusion that the response is not indicative of the hypothesis, a positive result could be due to several mechanisms.

Results included in WOE: There was no effect of treatment on the mean number of implantations and of live fetuses, and on the incidence of non-live implants and resorptions.

[5] ***Saillenfait and colleagues (2003)*** The developmental toxicity of ethylbenzene was studied in Sprague–Dawley rats after inhalation exposure. Animals were exposed to ethylbenzene at 100, 500, 1000 or 2000 ppm, for 6 h/day, during days 6–20 of gestation.

Limitations: This study was not specifically designed to investigate an endocrine mode of action. Although a negative result supports the conclusion that the response is not indicative of the hypothesis, a positive result could be due to several mechanisms.

Results included in WOE: The mean number of implantation sites per litter did not differ between any of the treatment groups and controls.

[6] ***Saillenfait and colleagues (2006)*** Pregnant Sprague–Dawley rats were exposed to ethylbenzene (EB; 0, 250, or 1000 ppm) and methylethylketone (MEK; 0, 1000, or 3000 ppm), alone and in combination, by inhalation, for 6 h/day, during days 6–20 of gestation.

Limitations: This study was not specifically designed to investigate an endocrine mode of action. Although a negative result supports the conclusion that the response is not indicative of the hypothesis, a positive result could be due to several mechanisms.

Results included in WOE: EB and MEK, alone or in combination, have no effect on the average number of implantations and live fetuses, and in the incidence of non-live implants and resorptions.

[13] ***Andrew et al., (1981); Hardin et al. (1981)*** Groups of 29-33 Female Wistar rats and New Zealand White rabbits were exposed to 0, 100, or 1000 ppm ethylbenzene for 7 h/day, 5 days/week for 3 weeks, then mated with unexposed males. Pregnant females were further exposed to 0, 100, or 1000 ppm 7 h/day through Gestational Day 19 (rats) and 24 (rabbits).

Limitations: This study was not specifically designed to investigate an endocrine mode of action. Although a negative result supports the conclusion that the response is not indicative of the hypothesis, a positive result could be due to several mechanisms.

Results included in the WOE: In both rats and rabbits, the number of implantations per doe and per corpora lutea was unaffected by exposure to 100 or to 1,000 ppm ethylbenzene relative to unexposed controls.

3.6 Rank 2: Developmental Toxicity – Litter size

[4] ***Saillenfait and colleagues (2007)*** The combined effects of EB and BA were investigated. Groups of 18 bred rats (15– 18 pregnant) were exposed to vapors of EB or BA, separately or in combination, 6 h day−1, on days 6–20 of gestation. There were nine experimental groups: Control; 250 or 1000 ppm EB; 500 or 1500 ppm BA or mixtures of 250 ppm EB + 500 ppm BA, 250 ppm EB + 1500 ppm BA, 1000 ppm EB + 500 ppm BA, or 1000 ppm EB + 1500 ppm BA

Limitations: This study was not specifically designed to investigate an endocrine mode of action. Although a negative result supports the conclusion that the response is not indicative of the hypothesis, a positive result could be due to several mechanisms.

Results included in WOE: There was no effect of treatment on the mean number of implantations and of live fetuses, and on the incidence of non-live implants and resorptions.

[5] ***Saillenfait and colleagues (2003)*** The developmental toxicity of ethylbenzene was studied in Sprague–Dawley rats after inhalation exposure. Animals were exposed to ethylbenzene at 100, 500, 1000 or 2000 ppm, for 6 h/day, during days 6–20 of gestation.

Limitations: Clinical signs of toxicity (ataxia, decreased motor activity) were seen at 2000 ppm. Maternal weight was significantly reduced on GD 21 at 1000 ppm and on GD 13 and 21 at 2000 ppm. Dams exposed to 1000 or 2000 ppm showed significant decreases in maternal weight gain and food consumption throughout exposure, and in corrected weight gain

Results included in WOE: The number of implantations was comparable among groups. Although the difference was not statistically significant, the incidence of non-live implants and resorptions was higher at 2000 ppm than in the control group. This was likely due to the 100% postimplantation loss seen in three of the 21 pregnant females exposed to 2000 ppm (0 in other groups).

[6] ***Saillenfait and colleagues (2006)*** Pregnant Sprague–Dawley rats were exposed to ethylbenzene (EB; 0, 250, or 1000 ppm) and methylethylketone (MEK; 0, 1000, or 3000 ppm), alone and in combination, by inhalation, for 6 h/day, during days 6–20 of gestation.

Limitations: This study was not specifically designed to investigate an endocrine mode of action. Although a negative result supports the conclusion that the response is not indicative of the hypothesis, a positive result could be due to several mechanisms.

Results included in WOE: EB and MEK, alone or in combination, have no effect on the average number of implantations and live fetuses, and in the incidence of non-live implants and resorptions.

[13] ***Andrew et al., (1981); Hardin et al. (1981)*** Groups of 29-33 Female Wistar rats and New Zealand White rabbits were exposed to 0, 100, or 1000 ppm ethylbenzene for 7 h/day, 5 days/week for 3 weeks, then mated with unexposed males. Pregnant females were further exposed to 0, 100, or 1000 ppm 7 h/day through Gestational Day 19 (rats) and 24 (rabbits).

Limitations: This study was not specifically designed to investigate an endocrine mode of action. Although a negative result supports the conclusion that the response is not indicative of the hypothesis, a positive result could be due to several mechanisms.

Results included in the WOE: Litter size, inferred from the number of live fetuses per litter, was slightly reduced in rabbits, but not in rats, exposed to ethylbenzene at 1,000 ppm, a concentration that produced some indications of maternal systemic effects. This finding is therefore unlikely to have been produced by and endocrine mode of action.

3.7 Rank 2: Developmental Toxicity – Sex ratio

[5] ***Saillenfait and colleagues (2003)*** The developmental toxicity of ethylbenzene was studied in Sprague–Dawley rats after inhalation exposure. Animals were exposed to ethylbenzene at 100, 500, 1000 or 2000 ppm, for 6 h/day, during days 6–20 of gestation.

Limitations: This study was not specifically designed to investigate an endocrine mode of action. Although a negative result supports the conclusion that the response is not indicative of the hypothesis, a positive result could be due to several mechanisms.

Results included in WOE: The percentage of males per litter did not differ between any of the treatments groups and controls.

[13] ***Andrew et al., (1981); Hardin et al. (1981)*** Groups of 29-33 Female Wistar rats and New Zealand White rabbits were exposed to 0, 100, or 1000 ppm ethylbenzene for 7 h/day, 5 days/week for 3 weeks, then mated with unexposed males. Pregnant females were further exposed to 0, 100, or 1000 ppm 7 h/day through Gestational Day 19 (rats) and 24 (rabbits).

Limitations: This study was not specifically designed to investigate an endocrine mode of action. Although a negative result supports the conclusion that the response is not indicative of the hypothesis, a positive result could be due to several mechanisms.

Results included in the WOE: Sex ratio in rats and rabbits was unaffected by exposure to 100 or to 1,000 ppm ethylbenzene relative to unexposed controls.

3.8 Rank 2: Reproductive Toxicity – Estrous cyclicity

[2] ***Faber and colleagues (2006)*** Four groups of Crl:CD(SD)IGS BR rats (30/sex/group for F_0 and 25/sex/group for F_1) were exposed to 0, 25, 100, and 500ppm EB for 6 h/day for at least 70 consecutive days before mating. Inhalation exposure for the F_0 and F_1 females continued throughout mating, gestation through gestation day (GD) 20, and lactation days (LD) 5–21. On LD 1–4, females received EB in corn oil via oral gavage at dose levels of 26, 90, and 342 mg/kg/day (divided into three equal doses, approximately 2 h apart).

Pups were weaned on postnatal day (PND) 21 and exposure of the F_1 generation started on PND 22. Estimates of internal exposure were determined by measuring EB concentrations in blood collected from F_1 dams (4/group) and their culled pups 1 h after the last gavage dose on PND 4. On PND 22, blood was collected from these same F_1 dams and their weanlings for EB analysis 1 h after a 6-h inhalation exposure. The remainder of the F_2 generation was not directly exposed.

Limitations: This study was not specifically designed to investigate an endocrine mode of action. Although a negative result supports the conclusion that the response is not indicative of the hypothesis, a positive result could be due to several mechanisms.

Results included in WOE: The mean estrous cycle length (4.0 ± 0.3days) was significantly reduced for the F_0 500ppm group when compared to the F_0 control group value (4.4 ± 0.8 days). However, the authors felt this difference was not biologically important because all females in this group were cycling normally and this strain of rat normally exhibits 4-5 day estrous cycles. Mean estrous cycle length did not differ between control and experimental F_1 offspring.

3.9 Rank 2: Reproductive Toxicity – Fertility

[2] ***Faber and colleagues (2006)*** Four groups of Crl:CD(SD)IGS BR rats (30/sex/group for F_0 and 25/sex/group for F_1) were exposed to 0, 25, 100, and 500ppm EB for 6 h/day for at least 70 consecutive days before mating. Inhalation exposure for the F_0 and F_1 females continued throughout mating, gestation through gestation day (GD) 20, and lactation days (LD) 5–21. On LD 1–4, females received EB in corn oil via oral gavage at dose levels of 26, 90, and 342 mg/kg/day (divided into three equal doses, approximately 2 h apart). Pups were weaned on postnatal day (PND) 21 and exposure of the F_1 generation started on PND 22. Estimates of internal exposure were determined by measuring EB concentrations in blood collected from F_1 dams (4/group) and their culled pups 1 h after the last gavage dose on PND 4. On PND 22, blood was collected from these same F_1 dams and their weanlings for EB analysis 1 h after a 6-h inhalation exposure. The remainder of the F_2 generation was not directly exposed.

Limitations: This study was not specifically designed to investigate an endocrine mode of action. Although a negative result supports the conclusion that the response is not indicative of the hypothesis, a positive result could be due to several mechanisms.

Results included in WOE: There was no impairment of fertility or increased time to mating in the F_0 or F_1 animals.

3.10 Rank 2: Reproductive Toxicity – Implantations

[2] ***Faber and colleagues (2006)*** Four groups of Crl:CD(SD)IGS BR rats (30/sex/group for F_0 and 25/sex/group for F_1) were exposed to 0, 25, 100, and 500ppm EB for 6 h/day for at least 70 consecutive days before mating. Inhalation exposure for the F_0 and F_1 females continued throughout mating, gestation through gestation day (GD) 20, and lactation days (LD) 5–21. On LD 1–4, females received EB in corn oil via oral gavage at dose levels of 26, 90, and 342 mg/kg/day (divided into three equal doses, approximately 2 h apart). Pups were weaned on postnatal day (PND) 21 and exposure of the F_1 generation started on PND 22. Estimates of internal exposure were determined by measuring EB concentrations in blood collected from F_1 dams (4/group) and their culled pups 1 hr after the last gavage dose on PND 4. On PND 22, blood was collected from these same F_1 dams and their weanlings for EB analysis 1 h after a 6-h inhalation exposure. The remainder of the F_2 generation was not directly exposed.

Limitations: This study was not specifically designed to investigate an endocrine mode of action. Although a negative result supports the conclusion that the response is not indicative of the hypothesis, a positive result could be due to several mechanisms.

Results included in WOE: No effects from ethylbenzene exposure of F_0 or F_1 rats were observed on reproductive performance parameters (mating and fertility indices, gestation lengths, former implantation sites and unaccounted sites).

3.11 Rank 2: Reproductive Toxicity – Litter size

[2] ***Faber and colleagues (2006)*** Four groups of Crl:CD(SD)IGS BR rats (30/sex/group for F_0 and 25/sex/group for F_1) were exposed to 0, 25, 100, and 500ppm EB for 6 h/day for at least 70 consecutive days before mating. Inhalation exposure for the F_0 and F_1 females continued throughout mating, gestation through gestation day (GD) 20, and lactation days (LD) 5–21. On LD 1–4, females received EB in corn oil via oral gavage at dose levels of 26, 90, and 342 mg/kg/day (divided into three equal doses, approximately 2 h apart). Pups were weaned on postnatal day (PND) 21 and exposure of the F_1 generation started on PND 22. Estimates of internal exposure were determined by measuring EB concentrations in blood collected from F_1 dams (4/group) and their culled pups 1 h after the last gavage dose on PND 4. On PND 22, blood was collected from these same F_1 dams and their weanlings for EB analysis 1 h after a 6-h inhalation exposure. The remainder of the F_2 generation was not directly exposed.

Limitations: This study was not specifically designed to investigate an endocrine mode of action. Although a negative result supports the conclusion that the response is not indicative of the hypothesis, a positive result could be due to several mechanisms.

Results included in WOE: The mean number of F_1 and F_2 pups born, live litter size, percentage of males per litter at birth, and postnatal survival were unaffected by ethylbenzene exposure.

3.12 Rank 2: Reproductive Toxicity – Mating index

[2] ***Faber and colleagues (2006)*** Four groups of Crl:CD(SD)IGS BR rats (30/sex/group for F_0 and 25/sex/group for F_1) were exposed to 0, 25, 100, and 500ppm EB for 6 h/day for at least 70 consecutive days before mating. Inhalation exposure for the F_0 and F_1 females continued throughout mating, gestation through gestation day (GD) 20, and lactation days (LD) 5–21. On LD 1–4, females received EB in corn oil via oral gavage at dose levels of 26, 90, and 342 mg/kg/day (divided into three equal doses, approximately 2 h apart). Pups were weaned on postnatal day (PND) 21 and exposure of the F_1 generation started on PND 22. Estimates of internal exposure were determined by measuring EB concentrations in blood collected from F_1 dams (4/group) and their culled pups 1 hr after the last gavage dose on PND 4. On PND 22, blood was collected from these same F_1 dams and their weanlings for EB analysis 1 h after a 6-h inhalation exposure. The remainder of the F_2 generation was not directly exposed.

Limitations: This study was not specifically designed to investigate an endocrine mode of action. Although a negative result supports the conclusion that the response is not indicative of the hypothesis, a positive result could be due to several mechanisms.

Results included in WOE: The male and female mating indices (%) were not different between the any of the treatment animals and controls.

3.13 Rank 2: Reproductive Toxicity – Prostate weight

[2] ***Faber and colleagues (2006)*** Four groups of Crl:CD(SD)IGS BR rats (30/sex/group for F_0 and 25/sex/group for F_1) were exposed to 0, 25, 100, and 500 ppm EB for 6 h/day for at least 70 consecutive days before mating. Inhalation exposure for the F_0 and F_1 females continued throughout mating, gestation through gestation day (GD) 20, and lactation days (LD) 5–21. On LD 1–4, females received EB in corn oil via oral gavage at dose levels of 26, 90, and 342 mg/kg/day (divided into three equal doses, approximately 2 h apart). Pups were weaned on postnatal day (PND) 21 and exposure of the F_1 generation started on PND 22. Estimates of internal exposure were determined by measuring EB concentrations in blood collected from F_1 dams (4/group) and their culled pups 1 h after the last gavage dose on PND 4. On PND 22, blood was collected from these same F_1 dams and their weanlings for EB analysis 1 h after a 6-hr inhalation exposure. The remainder of the F_2 generation was not directly exposed.

Limitations: This study was not specifically designed to investigate an endocrine mode of action. Although a negative result supports the conclusion that the response is not indicative of the hypothesis, a positive result could be due to several mechanisms.

Results included in WOE: There were statistically significant decreases in absolute prostate weights in the F_0 male 500 ppm group but not when these organ weights were expressed as relative to body weight. There was no significant difference in absolute or relative prostate weights in F_1 males.

3.14 Rank 2: Reproductive Toxicity – Sex ratio

[2] ***Faber and colleagues (2006)*** Four groups of Crl:CD(SD)IGS BR rats (30/sex/group for F_0 and 25/sex/group for F_1) were exposed to 0, 25, 100, and 500ppm EB for 6 h/day for at least 70 consecutive days before mating. Inhalation exposure for the F_0 and F_1 females continued throughout mating, gestation through gestation day (GD) 20, and lactation days (LD) 5–21. On LD 1–4, females received EB in corn oil via oral gavage at dose levels of 26, 90, and 342 mg/kg/day (divided into three equal doses, approximately 2 h apart). Pups were weaned on postnatal day (PND) 21 and exposure of the F_1 generation started on PND 22. Estimates of internal exposure were determined by measuring EB concentrations in blood collected from F_1 dams (4/group) and their culled pups 1 h after the last gavage dose on PND 4. On PND 22, blood was collected from these same F_1 dams and their weanlings for EB analysis 1 h after a 6-h inhalation exposure. The remainder of the F_2 generation was not directly exposed.

Limitations: This study was not specifically designed to investigate an endocrine mode of action. Although a negative result supports the conclusion that the response is not indicative of the hypothesis, a positive result could be due to several mechanisms.

Results included in WOE: The sex distribution, measured by the % males/litter, was not different in either F_1 or F_2 litters compared with control litters.

3.15 Rank 2: Reproductive Toxicity – Sperm count

[2] ***Faber and colleagues (2006)*** Four groups of Crl:CD(SD)IGS BR rats (30/sex/group for F_0 and 25/sex/group for F_1) were exposed to 0, 25, 100, and 500ppm EB for 6 h/day for at least 70 consecutive days before mating. Inhalation exposure for the F_0 and F_1 females continued throughout

mating, gestation through gestation day (GD) 20, and lactation days (LD) 5–21. On LD 1–4, females received EB in corn oil via oral gavage at dose levels of 26, 90, and 342 mg/kg/day (divided into three equal doses, approximately 2 h apart). Pups were weaned on postnatal day (PND) 21 and exposure of the F_1 generation started on PND 22. Estimates of internal exposure were determined by measuring EB concentrations in blood collected from F_1 dams (4/group) and their culled pups 1 h after the last gavage dose on PND 4. On PND 22, blood was collected from these same F_1 dams and their weanlings for EB analysis 1 h after a 6-h inhalation exposure. The remainder of the F_2 generation was not directly exposed.

Limitations: This study was not specifically designed to investigate an endocrine mode of action. Although a negative result supports the conclusion that the response is not indicative of the hypothesis, a positive result could be due to several mechanisms.

Results included in WOE: The mean sperm number (millions/g tissue) in the left cauda epididymis for F_0 and F_1 males were not different between any treatment group and controls.

3.16 Rank 2: Reproductive Toxicity – Time to balano-preputial separation

[2] ***Faber and colleagues (2006)*** Four groups of Crl:CD(SD)IGS BR rats (30/sex/group for F_0 and 25/sex/group for F_1) were exposed to 0, 25, 100, and 500 ppm EB for 6 h/day for at least 70 consecutive days before mating. Inhalation exposure for the F_0 and F_1 females continued throughout mating, gestation through gestation day (GD) 20, and lactation days (LD) 5–21. On LD 1–4, females received EB in corn oil via oral gavage at dose levels of 26, 90, and 342 mg/kg/day (divided into three equal doses, approximately 2 h apart). Pups were weaned on postnatal day (PND) 21 and exposure of the F_1 generation started on PND 22. Estimates of internal exposure were determined by measuring EB concentrations in blood collected from F_1 dams (4/group) and their culled pups 1 hr after the last gavage dose on PND 4. On PND 22, blood was collected from these same F_1 dams and their weanlings for EB analysis 1 h after a 6-h inhalation exposure. The remainder of the F_2 generation was not directly exposed.

Limitations: This study was not specifically designed to investigate an endocrine mode of action. Although a negative result supports the conclusion that the response is not indicative of the hypothesis, a positive result could be due to several mechanisms.

Results included in WOE: The mean age at acquisition of balanopreputial separation was significantly greater in the F_1 offspring in the 500-ppm treatment group compared with controls (PND 44.7± 2.0 vs. PND 43.5 ± 2.2). The mean value for the 500 ppm F_1 male group (PND 44.7) was similar to the value obtained in the F_2 generation control group (PND 45.3) and essentially equivalent to the mean historical control value (44.8 days) for the laboratory and as such, the authors stated that the significant finding was not considered biologically important. F_2 data for this measure were not published. However, the data is available in the full study report.

3.17 Rank 2: Reproductive Toxicity – Time to mating

[2] ***Faber and colleagues (2006)*** Four groups of Crl:CD(SD)IGS BR rats (30/sex/group for F_0 and 25/sex/group for F_1) were exposed to 0, 25, 100, and 500ppm EB for 6 h/day for at least 70 consecutive days before mating. Inhalation exposure for the F_0 and F_1 females continued throughout mating, gestation through gestation day (GD) 20, and lactation days (LD) 5–21. On LD 1–4, females received EB in corn oil via oral gavage at dose levels of 26, 90, and 342 mg/kg/day (divided into three equal doses, approximately 2 h apart). Pups were weaned on postnatal day (PND)

21 and exposure of the F_1 generation started on PND 22. Estimates of internal exposure were determined by measuring EB concentrations in blood collected from F_1 dams (4/group) and their culled pups 1 h after the last gavage dose on PND 4. On PND 22, blood was collected from these same F_1 dams and their weanlings for EB analysis 1 h after a 6-h inhalation exposure. The remainder of the F_2 generation was not directly exposed.

Limitations: This study was not specifically designed to investigate an endocrine mode of action. Although a negative result supports the conclusion that the response is not indicative of the hypothesis, a positive result could be due to several mechanisms.

Results included in WOE: There was no impairment of fertility or increased time to mating in the F_0 or F_1 offspring.

3.18 Rank 2: Reproductive Toxicity – Time to vaginal patency

[2] ***Faber and colleagues (2006)*** Four groups of Crl:CD(SD)IGS BR rats (30/sex/group for F_0 and 25/sex/group for F_1) were exposed to 0, 25, 100, and 500ppm EB for 6 h/day for at least 70 consecutive days before mating. Inhalation exposure for the F_0 and F_1 females continued throughout mating, gestation through gestation day (GD) 20, and lactation days (LD) 5–21. On LD 1–4, females received EB in corn oil via oral gavage at dose levels of 26, 90, and 342 mg/kg/day (divided into three equal doses, approximately 2 h apart). Pups were weaned on postnatal day (PND) 21 and exposure of the F_1 generation started on PND 22. Estimates of internal exposure were determined by measuring EB concentrations in blood collected from F_1 dams (4/group) and their culled pups 1 h after the last gavage dose on PND 4. On PND 22, blood was collected from these same F_1 dams and their weanlings for EB analysis 1 h after a 6-h inhalation exposure. The remainder of the F_2 generation was not directly exposed.

Limitations: This study was not specifically designed to investigate an endocrine mode of action. Although a negative result supports the conclusion that the response is not indicative of the hypothesis, a positive result could be due to several mechanisms.

Results included in WOE: The mean age of acquisition of vaginal patency for all exposed groups (25, 100 and 500ppm ethylbenzene) was statistically significantly lower than the mean for the concurrent control group value in F_1 female offspring; similar differences were not observed in the F_2 female pups. The authors felt these differences were not biologically important because the mean values were comparable to the historical control mean value.

3.19 Rank 3: Repeat Dose Toxicity – Gross pathology

[3] ***Mellert and colleagues (2007)*** Ethylbenzene was administered to groups of male and female Wistar rats by gavage for 4 (*n* = 5/ dose/sex) and 13 weeks (*n* = 10/dose/sex) (OECD 408) at doses of 0 (vehicle control), 75, 250, and 750 mg/ kg bodyweight/day (mg/kg bw/day), administered am/pm as half doses.

Limitations: This study was not specifically designed to investigate an endocrine mode of action. Although a negative result supports the conclusion that the response is not indicative of the hypothesis, a positive result could be due to several mechanisms.

Results included in WOE: Gross pathological evaluation of the epididymis, mammary glands, ovaries, prostate, seminal vesicles, testes, thyroid, uterus, and vagina did not identify any adverse effects.

4. Androgen Antagonist Hypothesis

4.1 Rank 2: Repeat Dose Toxicity – Epididymal weight

[9] ***NTP (National Toxicology Program). Toxicity studies of ethylbenzene (CAS No. 100-41-4) in F344/N rats and B6C3F1 mice (inhalation studies) (1992)*** Inhalation toxicity of ethylbenzene was studied by exposing groups of seven-week-old F344/N rats and B6C3Fi mice of each sex to ethylbenzene vapor at chamber concentrations of 0, 100, 250, 500, 750, or 1000 ppm, 6 h per day, 5 days per week for 13 weeks.

Limitations: This study was not specifically designed to investigate an endocrine mode of action. Although a negative result supports the conclusion that the response is not indicative of the hypothesis, a positive result could be due to several mechanisms. Additionally, high doses were used. It is unclear if the endpoint responses are secondary to general toxicity.

Results included in WOE: The tabular data indicate there was a significant decrease in the epididymal weight in <u>mice</u> exposed to ethylbenzene in the 1000-ppm group. The authors note that this was not considered biologically significant since spermatid counts, sperm motility, and caudal weight were normal. The narrative portion of the report states that this significant difference was found in the epididymal weight of rats, not mice – a likely error (p. 17). The tabular data show that there was no difference in the epididymal weight of <u>rats</u> at any ethylbenzene exposure level.

4.2 Rank 2: Repeat Dose Toxicity – Epididymis histopathology

[1] ***NTP (National Toxicology Program). (1999)*** Groups of 50 male and 50 female Fischer rats and B6C3F1 mice were exposed to ethylbenzene by inhalation at 0, 75, 250, and 750 ppm 6 h per day, 5 days per week, for 104 weeks (rats) and for 103 week (mice).

Limitations: This study was not specifically designed to investigate an endocrine mode of action. Although a negative result supports the conclusion that the response is not indicative of the hypothesis, a positive result could be due to several mechanisms.

Results included in WOE: There were no significant histological differences between the chamber control animals and treatment group animals, in either specie, for uterine, vaginal or ovarian tissues in females or for testes, prostate, epididymides or seminal vesicles for males.

[3] ***Mellert and colleagues (2007)*** Ethylbenzene was administered to groups of male and female Wistar rats by gavage for 4 (*n* = 5/ dose/sex) and 13 weeks (*n* = 10/dose/sex) (OECD 408) at doses of 0 (vehicle control), 75, 250, and 750 mg/kg bw/day, administered am/pm as half doses.

Limitations: This study was not specifically designed to investigate an endocrine mode of action. Although a negative result supports the conclusion that the response is not indicative of the hypothesis, a positive result could be due to several mechanisms.

Results included in WOE: Histopathological evaluation of the epididymis, mammary glands, ovaries, prostate, seminal vesicles, testes, thyroid, uterus, and vagina did not identify any adverse effects.

[9] ***NTP (National Toxicology Program). Toxicity studies of ethylbenzene (CAS No. 100-41-4) in F344/N rats and B6C3F1 mice (inhalation studies) (1992)*** Inhalation toxicity of ethylbenzene was

studied by exposing groups of seven-week-old F344/N rats and B6C3Fi mice of each sex to ethylbenzene vapor at chamber concentrations of 0, 100, 250, 500, 750, or 1000 ppm, 6 h per day, 5 days per week for 13 weeks.

Limitations: This study was not specifically designed to investigate an endocrine mode of action. Although a negative result supports the conclusion that the response is not indicative of the hypothesis, a positive result could be due to several mechanisms.

Results included in WOE: There were no histopathologic lesions and no chemically related histopathologic changes identified in the epididymides of mice or rats compared with controls.

[10] Cragg and colleagues (1989) Mice, rats and rabbits (five/sex/group) were exposed by inhalation to ethylbenzene vapors for 6 h/day, 5 days/week for 4 weeks (20 exposures). Rats and mice received 0, 99, 382 or 782 ppm EB while rabbits received 0, 382, 782 or 1610 ppm.

Limitations: This study was not specifically designed to investigate an endocrine mode of action. Although a negative result supports the conclusion that the response is not indicative of the hypothesis, a positive result could be due to several mechanisms.

Results included in WOE: The gonads (ovaries or testes with epididymides) and thyroids of high-exposure and controls animals of all species were subjected to histopathological examination. There were no gross or microscopic changes attributable to ethylbenzene in any of these tissues.

4.3 Rank 2: Repeat Dose Toxicity – Ovary histopathology

[1] ***NTP (National Toxicology Program). (1999)*** Groups of 50 male and 50 female Fischer rats and B6C3F1 mice were exposed to ethylbenzene by inhalation at 0, 75, 250, and 750 ppm 6 h per day, 5 days per week, for 104 weeks (rats) and for 103 week (mice).

Limitations: This study was not specifically designed to investigate an endocrine mode of action. Although a negative result supports the conclusion that the response is not indicative of the hypothesis, a positive result could be due to several mechanisms.

Results included in WOE: There were no significant histological differences between the chamber control animals and treatment group animals, in either specie, for uterine, vaginal or ovarian tissues in females or for testes, prostate, epididymides or seminal vesicles for males.

[3] ***Mellert and colleagues (2007)*** Ethylbenzene was administered to groups of male and female Wistar rats by gavage for 4 (*n* = 5/ dose/sex) and 13 weeks (*n* = 10/dose/sex) (OECD 408) at doses of 0 (vehicle control), 75, 250, and 750 mg/kg bw/day, administered am/pm as half doses.

Limitations: This study was not specifically designed to investigate an endocrine mode of action. Although a negative result supports the conclusion that the response is not indicative of the hypothesis, a positive result could be due to several mechanisms.

Results included in WOE: Histopathological evaluation of the epididymis, mammary glands, ovaries, prostate, seminal vesicles, testes, thyroid, uterus, and vagina did not identify any adverse effects.

[9] ***NTP (National Toxicology Program). Toxicity studies of ethylbenzene (CAS No. 100-41-4) in F344/N rats and B6C3F1 mice (inhalation studies) (1992)*** Inhalation toxicity of ethylbenzene was

studied by exposing groups of seven-week-old F344/N rats and B6C3Fi mice of each sex to ethylbenzene vapor at chamber concentrations of 0, 100, 250, 500, 750, or 1000 ppm, 6 h per day, 5 days per week for 13 weeks.

Limitations: This study was not specifically designed to investigate an endocrine mode of action. Although a negative result supports the conclusion that the response is not indicative of the hypothesis, a positive result could be due to several mechanisms.

Results included in WOE: There were no histopathologic lesions and no chemically related histopathologic changes identified in the ovaries of mice or rats compared with controls.

[10] Cragg and colleagues (1989) Mice, rats and rabbits (five/sex/group) were exposed by inhalation to ethylbenzene vapors for 6 h/day, 5 days/week for 4 weeks (20 exposures). Rats and mice received 0, 99, 382 or 782 ppm EB while rabbits received 0, 382, 782 or 1610 ppm.

Limitations: This study was not specifically designed to investigate an endocrine mode of action. Although a negative result supports the conclusion that the response is not indicative of the hypothesis, a positive result could be due to several mechanisms.

Results included in WOE: The gonads (ovaries or testes with epididymides) and thyroids of high-exposure and controls animals of all species were subjected to histopathological examination. There were no gross or microscopic changes attributable to ethylbenzene in any of these tissues.

4.4 Rank 2: Repeat Dose Toxicity – Prostate histopathology

[1] ***NTP (National Toxicology Program). (1999)*** Groups of 50 male and 50 female Fischer rats and B6C3F1 mice were exposed to ethylbenzene by inhalation at 0, 75, 250, and 750 ppm 6 h per day, 5 days per week, for 104 weeks (rats) and for 103 week (mice).

Limitations: This study was not specifically designed to investigate an endocrine mode of action. Although a negative result supports the conclusion that the response is not indicative of the hypothesis, a positive result could be due to several mechanisms.

Results included in WOE: There were no significant histological differences between the chamber control animals and treatment group animals, in either specie, for uterine, vaginal or ovarian tissues in females or for testes, prostate, epididymides or seminal vesicles for males.

[3] ***Mellert and colleagues (2007)*** Ethylbenzene was administered to groups of male and female Wistar rats by gavage for 4 (n = 5/ dose/sex) and 13 weeks (n = 10/dose/sex) (OECD 408) at doses of 0 (vehicle control), 75, 250, and mg/kg bw/day, administered am/pm as half doses.

Limitations: This study was not specifically designed to investigate an endocrine mode of action. Although a negative result supports the conclusion that the response is not indicative of the hypothesis, a positive result could be due to several mechanisms.

Results included in WOE: Histopathological evaluation of the epididymis, mammary glands, ovaries, prostate, seminal vesicles, testes, thyroid, uterus, and vagina did not identify any adverse effects.

[9] ***NTP (National Toxicology Program). Toxicity studies of ethylbenzene (CAS No. 100-41-4) in F344/N rats and B6C3F1 mice (inhalation studies) (1992)*** Inhalation toxicity of ethylbenzene was studied by exposing groups of seven-week-old F344/N rats and B6C3Fi mice of each sex to ethylbenzene vapor at chamber concentrations of 0, 100, 250, 500, 750, or 1000 ppm, 6 h per day, 5 days per week for 13 weeks.

Limitations: This study was not specifically designed to investigate an endocrine mode of action. Although a negative result supports the conclusion that the response is not indicative of the hypothesis, a positive result could be due to several mechanisms.

Results included in WOE: There were no histopathologic lesions and no chemically related histopathologic changes identified in the prostates of mice or rats compared with controls.

4.5 Rank 2: Repeat Dose Toxicity – Seminal vesicle histopathology

[1] ***NTP (National Toxicology Program). (1999)*** Groups of 50 male and 50 female Fischer rats and B6C3F1 mice were exposed to ethylbenzene by inhalation at 0, 75, 250, and 750 ppm 6 h per day, 5 days per week, for 104 weeks (rats) and for 103 week (mice).

Limitations: This study was not specifically designed to investigate an endocrine mode of action. Although a negative result supports the conclusion that the response is not indicative of the hypothesis, a positive result could be due to several mechanisms.

Results included in WOE: There were no significant histological differences between the chamber control animals and treatment group animals, in either specie, for uterine, vaginal or ovarian tissues in females or for testes, prostate, epididymides or seminal vesicles for males.

[3] ***Mellert and colleagues (2007)*** Ethylbenzene was administered to groups of male and female Wistar rats by gavage for 4 (*n* = 5/ dose/sex) and 13 weeks (*n* = 10/dose/sex) (OECD 408) at doses of 0 (vehicle control), 75, 250, and 750 mg/kg bw/day, administered am/pm as half doses.

Limitations: This study was not specifically designed to investigate an endocrine mode of action. Although a negative result supports the conclusion that the response is not indicative of the hypothesis, a positive result could be due to several mechanisms.

Results included in WOE: Histopathological evaluation of the epididymis, mammary glands, ovaries, prostate, seminal vesicles, testes, thyroid, uterus, and vagina did not identify any adverse effects.

[9] ***NTP (National Toxicology Program). Toxicity studies of ethylbenzene (CAS No. 100-41-4) in F344/N rats and B6C3F1 mice (inhalation studies) (1992)*** Inhalation toxicity of ethylbenzene was studied by exposing groups of seven-week-old F344/N rats and B6C3Fi mice of each sex to ethylbenzene vapor at chamber concentrations of 0, 100, 250, 500, 750, or 1000 ppm, 6 h per day, 5 days per week for 13 weeks.

Limitations: This study was not specifically designed to investigate an endocrine mode of action. Although a negative result supports the conclusion that the response is not indicative of the hypothesis, a positive result could be due to several mechanisms.

Results included in WOE: There were no histopathologic lesions and no chemically related histopathologic changes identified in the seminal vesicle of mice or rats compared with controls.

4.6 Rank 2: Repeat Dose – Testis histopathology (atrophy)

[1] ***NTP (National Toxicology Program). (1999)*** Groups of 50 male and 50 female Fischer rats and B6C3F1 mice were exposed to ethylbenzene by inhalation at 0, 75, 250, and 750 ppm 6 h per day, 5 days per week, for 104 weeks (rats) and for 103 week (mice).

Limitations: This study was not specifically designed to investigate an endocrine mode of action. Although a negative result supports the conclusion that the response is not indicative of the hypothesis, a positive result could be due to several mechanisms.

Results included in WOE: There were no significant histological differences between the chamber control animals and treatment group animals, in either specie, for uterine, vaginal or ovarian tissues in females or for testes, prostate, epididymides or seminal vesicles for males.

[3] ***Mellert and colleagues (2007)*** Ethylbenzene was administered to groups of male and female Wistar rats by gavage for 4 (*n* = 5/ dose/sex) and 13 weeks (*n* = 10/dose/sex) (OECD 408) at doses of 0 (vehicle control), 75, 250, and 750 mg/kg bw/day, administered am/pm as half doses.

Limitations: This study was not specifically designed to investigate an endocrine mode of action. Although a negative result supports the conclusion that the response is not indicative of the hypothesis, a positive result could be due to several mechanisms.

Results included in WOE: Histopathological evaluation of the epididymis, mammary glands, ovaries, prostate, seminal vesicles, testes, thyroid, uterus, and vagina did not identify any adverse effects.

[9] ***NTP (National Toxicology Program). Toxicity studies of ethylbenzene (CAS No. 100-41-4) in F344/N rats and B6C3F1 mice (inhalation studies) (1992)*** Inhalation toxicity of ethylbenzene was studied by exposing groups of seven-week-old F344/N rats and B6C3Fi mice of each sex to ethylbenzene vapor at chamber concentrations of 0, 100, 250, 500, 750, or 1000 ppm, 6 h per day, 5 days per week for 13 weeks.

Limitations: This study was not specifically designed to investigate an endocrine mode of action. Although a negative result supports the conclusion that the response is not indicative of the hypothesis, a positive result could be due to several mechanisms.

Results included in WOE: There were no histopathologic lesions and no chemically related histopathologic changes identified in testes of mice or rats compared with controls.

[10] Cragg and colleagues (1989) Mice, rats and rabbits (five/sex/group) were exposed by inhalation to ethylbenzene vapors for 6 h/day, 5 days/week for 4 weeks (20 exposures). Rats and mice received 0, 99, 382 or 782 ppm EB while rabbits received 0, 382, 782 or 1610 ppm.

Limitations: This study was not specifically designed to investigate an endocrine mode of action. Although a negative result supports the conclusion that the response is not indicative of the hypothesis, a positive result could be due to several mechanisms.

Results included in WOE: The gonads (ovaries or testes with epididymides) and thyroids of high-exposure and controls animals of all species were subjected to histopathological examination. There were no gross or microscopic changes attributable to ethylbenzene in any of these tissues.

4.7 Rank 2: Repeat Dose Toxicity – Testis weight

[9] ***NTP (National Toxicology Program). Toxicity studies of ethylbenzene (CAS No. 100-41-4) in F344/N rats and B6C3F1 mice (inhalation studies) (1992)*** Inhalation toxicity of ethylbenzene was studied by exposing groups of seven-week-old F344/N rats and B6C3Fi mice of each sex to ethylbenzene vapor at chamber concentrations of 0, 100, 250, 500, 750, or 1000 ppm, 6 h per day, 5 days per week for 13 weeks.

Limitations: This study was not specifically designed to investigate an endocrine mode of action. Although a negative result supports the conclusion that the response is not indicative of the hypothesis, a positive result could be due to several mechanisms.

Results included in WOE: Weights of testes in mice and rats were not affected by ethylbenzene.

4.8 Rank 2: Repeat Dose Toxicity – Uterus histopathology

[1] ***NTP (National Toxicology Program). (1999)*** Groups of 50 male and 50 female Fischer rats and B6C3F1 mice were exposed to ethylbenzene by inhalation at 0, 75, 250, and 750 ppm 6 h per day, 5 days per week, for 104 weeks (rats) and for 103 week (mice).

Limitations: This study was not specifically designed to investigate an endocrine mode of action. Although a negative result supports the conclusion that the response is not indicative of the hypothesis, a positive result could be due to several mechanisms.

Results included in WOE: There were no significant histological differences between the chamber control animals and treatment group animals, in either specie, for uterine, vaginal or ovarian tissues in females or for testes, prostate, epididymides or seminal vesicles for males.

[3] ***Mellert and colleagues (2007)*** Ethylbenzene was administered to groups of male and female Wistar rats by gavage for 4 (n = 5/ dose/sex) and 13 weeks (n = 10/dose/sex) (OECD 408) at doses of 0 (vehicle control), 75, 250, and 750 mg/kg bw/day, administered am/pm as half doses.

Limitations: This study was not specifically designed to investigate an endocrine mode of action. Although a negative result supports the conclusion that the response is not indicative of the hypothesis, a positive result could be due to several mechanisms.

Results included in WOE: Histopathological evaluation of the epididymis, mammary glands, ovaries, prostate, seminal vesicles, testes, thyroid, uterus, and vagina did not identify any adverse effects.

[9] ***NTP (National Toxicology Program). Toxicity studies of ethylbenzene (CAS No. 100-41-4) in F344/N rats and B6C3F1 mice (inhalation studies) (1992)*** Inhalation toxicity of ethylbenzene was studied by exposing groups of seven-week-old F344/N rats and B6C3Fi mice of each sex to ethylbenzene vapor at chamber concentrations of 0, 100, 250, 500, 750, or 1000 ppm, 6 h per day, 5 days per week for 13 weeks.

Limitations: This study was not specifically designed to investigate an endocrine mode of action. Although a negative result supports the conclusion that the response is not indicative of the hypothesis, a positive result could be due to several mechanisms.

Results included in WOE: There were no histopathologic lesions and no chemically related histopathologic changes identified in the uteri of mice or rats compared with controls.

[10] Cragg and colleagues (1989) Mice, rats and rabbits (five/sex/group) were exposed by inhalation to ethylbenzene vapors for 6 h/day, 5 days/week for 4 weeks (20 exposures). Rats and mice received 0, 99, 382 or 782 ppm EB while rabbits received 0, 382, 782 or 1610 ppm.

Limitations: This study was not specifically designed to investigate an endocrine mode of action. Although a negative result supports the conclusion that the response is not indicative of the hypothesis, a positive result could be due to several mechanisms.

Results included in WOE: The uterus of high-exposure and controls animals of all species was subjected to histopathological examination. There were no gross or microscopic changes attributable to ethylbenzene in this organ.

4.9 Rank 2: Reproductive Toxicity – Estrous cyclicity

[2] ***Faber and colleagues (2006)*** Four groups of Crl:CD(SD)IGS BR rats (30/sex/group for F_0 and 25/sex/group for F_1) were exposed to 0, 25, 100, and 500ppm EB for 6 h/day for at least 70 consecutive days before mating. Inhalation exposure for the F_0 and F_1 females continued throughout mating, gestation through gestation day (GD) 20, and lactation days (LD) 5–21. On LD 1–4, females received EB in corn oil via oral gavage at dose levels of 26, 90, and 342 mg/kg/day (divided into three equal doses, approximately 2 h apart). Pups were weaned on postnatal day (PND) 21 and exposure of the F_1 generation started on PND 22. Estimates of internal exposure were determined by measuring EB concentrations in blood collected from F_1 dams (4/group) and their culled pups 1 h after the last gavage dose on PND 4. On PND 22, blood was collected from these same F_1 dams and their weanlings for EB analysis 1 h after a 6-h inhalation exposure. The remainder of the F_2 generation was not directly exposed.

Limitations: This study was not specifically designed to investigate an endocrine mode of action. Although a negative result supports the conclusion that the response is not indicative of the hypothesis, a positive result could be due to several mechanisms.

Results included in WOE: The mean estrous cycle length (4.0 ± 0.3days) was significantly reduced for the F_0, 500ppm group when compared to the F_0 control group value (4.4 ± 0.8 days). However, the authors felt this difference was not biologically important because all females in this group were cycling normally and this strain of rat normally exhibits 4- to 5 day estrous cycles. Mean estrous cycle length did not differ between control and experimental F_1 offspring.

4.10 Rank 2: Reproductive Toxicity – Fertility

[2] ***Faber and colleagues (2006)*** Four groups of Crl:CD(SD)IGS BR rats (30/sex/group for F_0 and 25/sex/group for F_1) were exposed to 0, 25, 100, and 500ppm EB for 6 h/day for at least 70 consecutive days before mating. Inhalation exposure for the F_0 and F_1 females continued throughout mating, gestation through gestation day (GD) 20, and lactation days (LD) 5–21. On LD 1–4, females received EB in corn oil via oral gavage at dose levels of 26, 90, and 342 mg/kg/day (divided into three

equal doses, approximately 2 h apart). Pups were weaned on postnatal day (PND) 21 and exposure of the F_1 generation started on PND 22. Estimates of internal exposure were determined by measuring EB concentrations in blood collected from F_1 dams (4/group) and their culled pups 1 h after the last gavage dose on PND 4. On PND 22, blood was collected from these same F_1 dams and their weanlings for EB analysis 1 h after a 6-h inhalation exposure. The remainder of the F_2 generation was not directly exposed.

Limitations: This study was not specifically designed to investigate an endocrine mode of action. Although a negative result supports the conclusion that the response is not indicative of the hypothesis, a positive result could be due to several mechanisms.

Results included in WOE: There was no impairment of fertility or increased time to mating in the F_0 or F_1 animals.

4.11 Rank 2: Reproductive Toxicity – Gross pathology

[2] ***Faber and colleagues (2006)*** Four groups of Crl:CD(SD)IGS BR rats (30/sex/group for F_0 and 25/sex/group for F_1) were exposed to 0, 25, 100, and 500ppm EB for 6 h/day for at least 70 consecutive days before mating. Inhalation exposure for the F_0 and F_1 females continued throughout mating, gestation through gestation day (GD) 20, and lactation days (LD) 5–21. On LD 1–4, females received EB in corn oil via oral gavage at dose levels of 26, 90, and 342 mg/kg/day (divided into three equal doses, approximately 2 h apart). Pups were weaned on postnatal day (PND) 21 and exposure of the F_1 generation started on PND 22. Estimates of internal exposure were determined by measuring EB concentrations in blood collected from F_1 dams (4/group) and their culled pups 1 h after the last gavage dose on PND 4. On PND 22, blood was collected from these same F_1 dams and their weanlings for EB analysis 1 h after a 6-hr inhalation exposure. The remainder of the F_2 generation was not directly exposed.

Limitations: This study was not specifically designed to investigate an endocrine mode of action. Although a negative result supports the conclusion that the response is not indicative of the hypothesis, a positive result could be due to several mechanisms.

Results included in WOE: No adverse exposure-related macroscopic pathology was noted at any level.

4.12 Rank 2: Reproductive Toxicity – Litter size

[2] ***Faber and colleagues (2006)*** Four groups of Crl:CD(SD)IGS BR rats (30/sex/group for F_0 and 25/sex/group for F_1) were exposed to 0, 25, 100, and 500ppm EB for 6 h/day for at least 70 consecutive days before mating. Inhalation exposure for the F_0 and F_1 females continued throughout mating, gestation through gestation day (GD) 20, and lactation days (LD) 5–21. On LD 1–4, females received EB in corn oil via oral gavage at dose levels of 26, 90, and 342 mg/kg/day (divided into three equal doses, approximately 2 h apart). Pups were weaned on postnatal day (PND) 21 and exposure of the F_1 generation started on PND 22. Estimates of internal exposure were determined by measuring EB concentrations in blood collected from F_1 dams (4/group) and their culled pups 1 h after the last gavage dose on PND 4. On PND 22, blood was collected from these same F_1 dams and their weanlings for EB analysis 1 h after a 6-h inhalation exposure. The remainder of the F_2 generation was not directly exposed.

Limitations: This study was not specifically designed to investigate an endocrine mode of action. Although a negative result supports the conclusion that the response is not indicative of the hypothesis, a positive result could be due to several mechanisms.

Results included in WOE: The mean number of F_1 and F_2 pups born, live litter size, percentage of males per litter at birth, and postnatal survival were unaffected by ethylbenzene exposure.

4.13 Rank 2: Reproductive Toxicity – Prostate weight

[2] ***Faber and colleagues (2006)*** Four groups of Crl:CD(SD)IGS BR rats (30/sex/group for F_0 and 25/sex/group for F_1) were exposed to 0, 25, 100, and 500ppm EB for 6 h/day for at least 70 consecutive days before mating. Inhalation exposure for the F_0 and F_1 females continued throughout mating, gestation through gestation day (GD) 20, and lactation days (LD) 5–21. On LD 1–4, females received EB in corn oil via oral gavage at dose levels of 26, 90, and 342 mg/kg/day (divided into three equal doses, approximately 2 h apart). Pups were weaned on postnatal day (PND) 21 and exposure of the F_1 generation started on PND 22. Estimates of internal exposure were determined by measuring EB concentrations in blood collected from F_1 dams (4/group) and their culled pups 1 h after the last gavage dose on PND 4. On PND 22, blood was collected from these same F_1 dams and their weanlings for EB analysis 1 h after a 6-h inhalation exposure. The remainder of the F_2 generation was not directly exposed.

Limitations: This study was not specifically designed to investigate an endocrine mode of action. Although a negative result supports the conclusion that the response is not indicative of the hypothesis, a positive result could be due to several mechanisms.

Results included in WOE: There were statistically significant decreases in absolute prostate weights in the F_0 male 500ppm group but not when these organ weights were expressed relative to body weight. There was no significant difference in absolute or relative prostate weights in F_1 males.

4.14 Rank 2: Reproductive Toxicity – Sperm count

[2] ***Faber and colleagues (2006)*** Four groups of Crl:CD(SD)IGS BR rats (30/sex/group for F_0 and 25/sex/group for F_1) were exposed to 0, 25, 100, and 500ppm EB for 6 h/day for at least 70 consecutive days before mating. Inhalation exposure for the F_0 and F_1 females continued throughout mating, gestation through gestation day (GD) 20, and lactation days (LD) 5–21. On LD 1–4, females received EB in corn oil via oral gavage at dose levels of 26, 90, and 342 mg/kg/day (divided into three equal doses, approximately 2 h apart). Pups were weaned on postnatal day (PND) 21 and exposure of the F_1 generation started on PND 22. Estimates of internal exposure were determined by measuring EB concentrations in blood collected from F_1 dams (4/group) and their culled pups 1 h after the last gavage dose on PND 4. On PND 22, blood was collected from these same F_1 dams and their weanlings for EB analysis 1 h after a 6-h inhalation exposure. The remainder of the F_2 generation was not directly exposed.

Limitations: This study was not specifically designed to investigate an endocrine mode of action. Although a negative result supports the conclusion that the response is not indicative of the hypothesis, a positive result could be due to several mechanisms.

Results included in WOE: The mean sperm number (millions/g tissue) in the left cauda epididymis for F_0 and F_1 males were not significantly different between any treatment group and controls.

4.15 Rank 2: Reproductive Toxicity – Sperm motility

[2] ***Faber and colleagues (2006)*** Four groups of Crl:CD(SD)IGS BR rats (30/sex/group for F_0 and 25/sex/group for F_1) were exposed to 0, 25, 100, and 500ppm EB for 6 h/day for at least 70 consecutive days before mating. Inhalation exposure for the F_0 and F_1 females continued throughout mating, gestation through gestation day (GD) 20, and lactation days (LD) 5–21. On LD 1–4, females received EB in corn oil via oral gavage at dose levels of 26, 90, and 342 mg/kg/day (divided into three equal doses, approximately 2 h apart). Pups were weaned on postnatal day (PND) 21 and exposure of the F_1 generation started on PND 22. Estimates of internal exposure were determined by measuring EB concentrations in blood collected from F_1 dams (4/group) and their culled pups 1 h after the last gavage dose on PND 4. On PND 22, blood was collected from these same F_1 dams and their weanlings for EB analysis 1 h after a 6-h inhalation exposure. The remainder of the F_2 generation was not directly exposed.

Limitations: This study was not specifically designed to investigate an endocrine mode of action. Although a negative result supports the conclusion that the response is not indicative of the hypothesis, a positive result could be due to several mechanisms.

Results included in WOE: The mean percentage of motile sperm did not differ significantly between any of the treatment group animals compared with controls.

4.16 Rank 2: Reproductive Toxicity – Time to balano-preputial separation

[2] ***Faber and colleagues (2006)*** Four groups of Crl:CD(SD)IGS BR rats (30/sex/group for F_0 and 25/sex/group for F_1) were exposed to 0, 25, 100, and 500ppm EB for 6 hr/day for at least 70 consecutive days before mating. Inhalation exposure for the F_0 and F_1 females continued throughout mating, gestation through gestation day (GD) 20, and lactation days (LD) 5–21. On LD 1–4, females received EB in corn oil via oral gavage at dose levels of 26, 90, and 342 mg/kg/day (divided into three equal doses, approximately 2 hr apart). Pups were weaned on postnatal day (PND) 21 and exposure of the F_1 generation started on PND 22. Estimates of internal exposure were determined by measuring EB concentrations in blood collected from F_1 dams (4/group) and their culled pups 1 hr after the last gavage dose on PND 4. On PND 22, blood was collected from these same F_1 dams and their weanlings for EB analysis 1 hr after a 6-hr inhalation exposure. The remainder of the F_2 generation was not directly exposed.

Limitations: This study was not specifically designed to investigate an endocrine mode of action. Although a negative result supports the conclusion that the response is not indicative of the hypothesis, a positive result could be due to several mechanisms.

Results included in WOE: The mean age at acquisition of balanopreputial separation was significantly greater in the F_1 offspring in the 500ppm treatment group compared with controls (PND 44.7± 2.0 vs. PND 43.5 ± 2.2). The mean value for the 500ppm F_1 male group (PND 44.7) was similar to the value obtained in the F_2 generation control group (PND 45.3) and essentially equivalent to the mean historical control value (44.8 days) for the laboratory and as such, the authors stated that the significant finding was not considered biologically important. F_2 data for this measure were not published. However, the data is available in the full study report.

4.17 Rank 2: Reproductive Toxicity – Time to mating

[2] ***Faber and colleagues (2006)*** Four groups of Crl:CD(SD)IGS BR rats (30/sex/group for F_0 and 25/sex/group for F_1) were exposed to 0, 25, 100, and 500ppm EB for 6 h/day for at least 70 consecutive days before mating. Inhalation exposure for the F_0 and F_1 females continued throughout mating, gestation through gestation day (GD) 20, and lactation days (LD) 5–21. On LD 1–4, females received EB in corn oil via oral gavage at dose levels of 26, 90, and 342 mg/kg/day (divided into three equal doses, approximately 2 h apart). Pups were weaned on postnatal day (PND) 21 and exposure of the F_1 generation started on PND 22. Estimates of internal exposure were determined by measuring EB concentrations in blood collected from F_1 dams (4/group) and their culled pups 1 h after the last gavage dose on PND 4. On PND 22, blood was collected from these same F_1 dams and their weanlings for EB analysis 1 h after a 6-h inhalation exposure. The remainder of the F_2 generation was not directly exposed.

Limitations: This study was not specifically designed to investigate an endocrine mode of action. Although a negative result supports the conclusion that the response is not indicative of the hypothesis, a positive result could be due to several mechanisms.

Results included in WOE: There was no impairment of fertility or increased time to mating in the F_0 or F_1 animals.

4.18 Rank 3: Repeat Dose Toxicity – Gross pathology

[3] ***Mellert and colleagues (2007)*** Ethylbenzene was administered to groups of male and female Wistar rats by gavage for 4 (*n* = 5/ dose/sex) and 13 weeks (*n* = 10/dose/sex) (OECD 408) at doses of 0 (vehicle control), 75, 250, and 750 mg/kg bw/day, administered am/pm as half doses.

Limitations: This study was not specifically designed to investigate an endocrine mode of action. Although a negative result supports the conclusion that the response is not indicative of the hypothesis, a positive result could be due to several mechanisms.

Results included in WOE: Gross pathological evaluation of the epididymis, mammary glands, ovaries, prostate, seminal vesicles, testes, thyroid, uterus, and vagina did not identify any adverse effects.

5. Thyroid Inhibition Hypothesis

5.1 Rank 2: Repeat Dose Toxicity – Thyroid follicular cell histopathology

[1] ***NTP (National Toxicology Program). (1999)*** Groups of 50 male and 50 female Fischer rats and B6C3F1 mice were exposed to ethylbenzene by inhalation at 0, 75, 250, and 750 ppm 6 h per day, 5 days per week, for 104 weeks (rats) and for 103 week (mice).

Limitations: This study was not specifically designed to investigate an endocrine mode of action. Although a negative result supports the conclusion that the response is not indicative of the hypothesis, a positive result could be due to several mechanisms.

Results included in WOE: Positive trends in the incidences of thyroid follicular cell hyperplasia occurred in mice in both males (control: 21:50; 75 ppm: 21:50; 250 ppm: 29:50; 750 ppm: 32:50) and females (18:50, 23:50, 25:50, 35:50) with significant increases in incidences relative to chamber

controls in 750 ppm males and females. There were no significant differences between control and exposed rat thyroids upon histopathological examination.

[3] ***Mellert and colleagues (2007)*** Ethylbenzene was administered to groups of male and female Wistar rats by gavage for 4 (*n* = 5/ dose/sex) and 13 weeks (*n* = 10/dose/sex) (OECD 408) at doses of 0 (vehicle control), 75, 250, and 750 mg/kg bw/day, administered am/pm as half doses.

Limitations: This study was not specifically designed to investigate an endocrine mode of action. Although a negative result supports the conclusion that the response is not indicative of the hypothesis, a positive result could be due to several mechanisms.

Results included in WOE: Histopathological evaluation of the epididymis, mammary glands, ovaries, prostate, seminal vesicles, testes, thyroid, uterus, and vagina did not identify any adverse effects.

[9] ***NTP (National Toxicology Program). Toxicity studies of ethylbenzene (CAS No. 100-41-4) in F344/N rats and B6C3F1 mice (inhalation studies) (1992)*** Inhalation toxicity of ethylbenzene was studied by exposing groups of seven-week-old F344/N rats and B6C3Fi mice of each sex to ethylbenzene vapor at chamber concentrations of 0, 100, 250, 500, 750, or 1000 ppm, 6 h per day, 5 days per week for 13 weeks.

Limitations: This study was not specifically designed to investigate an endocrine mode of action. Although a negative result supports the conclusion that the response is not indicative of the hypothesis, a positive result could be due to several mechanisms.

Results included in WOE: There were no histopathologic changes seen in the thyroid glands of mice or rats.

[10] Cragg and colleagues (1989) Mice, rats and rabbits (five/sex/group) were exposed by inhalation to ethylbenzene vapors for 6 h/day, 5 days/week for 4 weeks (20 exposures). Rats and mice received 0, 99, 382 or 782 ppm EB while rabbits received 0, 382, 782 or 1610 ppm.

Limitations: This study was not specifically designed to investigate an endocrine mode of action. Although a negative result supports the conclusion that the response is not indicative of the hypothesis, a positive result could be due to several mechanisms.

Results included in WOE: The gonads (ovaries or testes with epididymides) and thyroids of high-exposure and controls animals of all species were subjected to histopathological examination. There were no gross or microscopic changes attributable to ethylbenzene in any of these tissues.

5.2 Rank 2: Developmental Toxicity – Fetal survival

[4] ***Saillenfait and colleagues (2007)*** The combined effects of EB and BA were investigated. Groups of 18 bred rats (15– 18 pregnant) were exposed to vapors of EB or BA, separately or in combination, 6 h day−1, on days 6–20 of gestation. There were nine experimental groups: Control; 250 or 1000 ppm EB; 500 or 1500 ppm BA or mixtures of 250 ppm EB + 500 ppm BA, 250 ppm EB + 1500 ppm BA, 1000 ppm EB + 500 ppm BA, or 1000 ppm EB + 1500 ppm BA

Limitations: This study was not specifically designed to investigate an endocrine mode of action. Although a negative result supports the conclusion that the response is not indicative of the hypothesis, a positive result could be due to several mechanisms.

Results included in WOE: There was no effect of treatment on the mean number of implantations and of live fetuses, and on the incidence of non-live implants and resorptions.

[5] ***Saillenfait and colleagues (2003)*** The developmental toxicity of ethylbenzene was studied in Sprague–Dawley rats after inhalation exposure. Animals were exposed to ethylbenzene at 100, 500, 1000 or 2000 ppm, for 6 h/day, during days 6–20 of gestation.

Limitations: This study was not specifically designed to investigate an endocrine mode of action. Although a negative result supports the conclusion that the response is not indicative of the hypothesis, a positive result could be due to several mechanisms.

Results included in WOE: The percent dead fetuses per litter was not significantly different between ethylbenzene treated and control dams.

[6] ***Saillenfait and colleagues (2006)*** Pregnant Sprague–Dawley rats were exposed to ethylbenzene (EB; 0, 250, or 1000 ppm) and methylethylketone (MEK; 0, 1000, or 3000 ppm), alone and in combination, by inhalation, for 6 h/day, during days 6–20 of gestation.

Limitations: This study was not specifically designed to investigate an endocrine mode of action. Although a negative result supports the conclusion that the response is not indicative of the hypothesis, a positive result could be due to several mechanisms.

Results included in WOE: There was no increase in embryolethality for fetuses whose mothers were exposed to ethylbenzene alone or in combination with methylethylketone.

[11] ***Ungváry, G. & Tátrai, E. (1985)*** Groups of CFY rats were exposed to inhalation of ethylbenzene at 0, 138, 276 or 553 ppm for 24 h/day from day 7 to day 15 of pregnancy. Fetuses were evaluated on pregnancy day 21. CFLP mice were exposed to inhalation of ethylbenzene at 0, 115 or 230 ppm for 24 h/day (no data provided for these groups) or for 3-4 h/day intermittently from day 6 to 15 of pregnancy. The fetuses were evaluated on pregnancy days 18. NZ rabbits were exposed to 0, 115, or 230 ppm ethylbenzene for 24 h/day from day 7 to day 20 gestation. Fetuses were examined on pregnancy day 30. The three rabbit does in the 230-ppm dose group aborted.

Limitations: The data for mice was only provided for the animals in the 115-ppm exposure group and maternal toxicity information was lacking. The authors mention that the maternal toxic effects of ethylbenzene in rats were “moderate and dose-dependent” but fail to describe or quantify these effects. The contribution of general toxicity effects to all study findings should be considered.

Results included in WOE: All rabbit dams (3/3) in the 230 ppm dose group aborted resulting in the loss of all fetuses.

[13] ***Andrew et al., (1981); Hardin et al. (1981)*** Groups of 29-33 Female Wistar rats and New Zealand White rabbits were exposed to 0, 100, or 1000 ppm ethylbenzene for 7 h/day, 5 days/week for 3 weeks, then mated with unexposed males. Pregnant females were further exposed to 0, 100, or 1000 ppm 7 h/day through Gestational Day 19 (rats) and 24 (rabbits).

Limitations: This study was not specifically designed to investigate an endocrine mode of action. Although a negative result supports the conclusion that the response is not indicative of the hypothesis, a positive result could be due to several mechanisms.

Results included in the WOE: The number of implantations was comparable among groups. The number of live fetuses was slightly reduced in rabbits, but not in rats, exposed to ethylbenzene at 1,000 ppm, a concentration that produced some indications of maternal systemic effects. This finding is therefore unlikely to have been produced by and endocrine mode of action.

5.3 Rank 2: Developmental Toxicity – Fetal weight

[4] ***Saillenfait and colleagues (2007)*** The combined effects of EB and BA were investigated. Groups of 18 bred rats (15– 18 pregnant) were exposed to vapors of EB or BA, separately or in combination, 6 h day−1, on days 6–20 of gestation. There were nine experimental groups: Control; 250 or 1000 ppm EB; 500 or 1500 ppm BA or mixtures of 250 ppm EB + 500 ppm BA, 250 ppm EB + 1500 ppm BA, 1000 ppm EB + 500 ppm BA, or 1000 ppm EB + 1500 ppm BA

Limitations: This study was not specifically designed to investigate an endocrine mode of action. Although a negative result supports the conclusion that the response is not indicative of the hypothesis, a positive result could be due to several mechanisms. Additionally, high doses were used. It is unclear if the endpoint responses are secondary to general toxicity.

Results included in WOE: Fetal body weight was significantly decreased after exposure to 1000 ppm EB alone.

[5] ***Saillenfait and colleagues (2003)*** The developmental toxicity of ethylbenzene was studied in Sprague–Dawley rats after inhalation exposure. Animals were exposed to ethylbenzene at 100, 500, 1000 or 2000 ppm, for 6 h/day, during days 6–20 of gestation.

Limitations: Clinical signs of toxicity (ataxia, decreased motor activity) were seen at 2000 ppm. Maternal weight was significantly reduced on GD 21 at 1000 ppm and on GD 13 and 21 at 2000 ppm. Dams exposed to 1000 or 2000 ppm showed significant decreases in maternal weight gain and food consumption throughout exposure, and in corrected weight gain

Results included in WOE: Ethylbenzene produced a concentration-related reduction in fetal weights that achieved statistical significance at 1000 ppm. These decreases amounted to 7 and 18% of the control values at 1000 and 2000 ppm, respectively.

[6] ***Saillenfait and colleagues (2006)*** Pregnant Sprague–Dawley rats were exposed to ethylbenzene (EB; 0, 250, or 1000 ppm) and methylethylketone (MEK; 0, 1000, or 3000 ppm), alone and in combination, by inhalation, for 6 h/day, during days 6–20 of gestation.

Limitations: This study was not specifically designed to investigate an endocrine mode of action. Although a negative result supports the conclusion that the response is not indicative of the hypothesis, a positive result could be due to several mechanisms. Additionally, high doses were used. It is unclear if the endpoint responses are secondary to general toxicity.

Results included in WOE: The body weight of the fetuses (all, males, females) was significantly lower than control after exposure to the high concentration of EB, 1000 ppm.

[11] ***Ungváry, G. & Tátrai, E. (1985)*** Groups of CFY rats were exposed to inhalation of ethylbenzene at 0, 138, 276 or 553 ppm for 24 h/day from day 7 to day 15 of pregnancy. Fetuses were evaluated on pregnancy day 21. CFLP mice were exposed to inhalation of ethylbenzene at 0, 115 or 230 ppm for 24 h/day (no data provided for these groups) or for 3-4 h/day intermittently from day 6 to 15 of pregnancy. The fetuses were evaluated on pregnancy days 18. NZ rabbits were exposed to 0, 115, or 230 ppm ethylbenzene for 24 h/day from day 7 to day 20 gestation. Fetuses were examined on pregnancy day 30. The three rabbit does in the 230-ppm dose group aborted.

Limitations: The data for mice was only provided for the animals in the 115-ppm exposure group and maternal toxicity information was lacking. The authors mention that the maternal toxic effects of ethylbenzene in rats were "moderate and dose-dependent" but fail to describe or quantify these effects. The contribution of general toxicity effects to all study findings should be considered.

Results included in WOE: The percentage of weight-retarded fetuses was significantly greater in the group of rats exposed to ethylbenzene at a concentration of 553 ppm and in female rabbit fetuses at 115 ppm compared with controls. There was not a significant difference in mean fetal weights in mice exposed to ethylbenzene 3-4 hours/day intermittently at 115 ppm.

[13] ***Andrew et al., (1981); Hardin et al. (1981)*** Groups of 29-33 Female Wistar rats and New Zealand White rabbits were exposed to 0, 100, or 1000 ppm ethylbenzene for 7 h/day, 5 days/week for 3 weeks, then mated with unexposed males. Pregnant females were further exposed to 0, 100, or 1000 ppm 7 h/day through Gestational Day 19 (rats) and 24 (rabbits).

Limitations: This study was not specifically designed to investigate an endocrine mode of action. Although a negative result supports the conclusion that the response is not indicative of the hypothesis, a positive result could be due to several mechanisms.

Results included in the WOE: Fetal weights were unchanged relative to controls in rats or rabbit exposed to 100 or to 1,000 ppm ethylbenzene.

5.4 Rank 2: Reproductive Toxicity – Pup growth

[2] ***Faber and colleagues (2006)*** Four groups of Crl:CD(SD)IGS BR rats (30/sex/group for F_0 and 25/sex/group for F_1) were exposed to 0, 25, 100, and 500ppm EB for 6 h/day for at least 70 consecutive days before mating. Inhalation exposure for the F_0 and F_1 females continued throughout mating, gestation through gestation day (GD) 20, and lactation days (LD) 5–21. On LD 1–4, females received EB in corn oil via oral gavage at dose levels of 26, 90, and 342 mg/kg/day (divided into three equal doses, approximately 2 h apart). Pups were weaned on postnatal day (PND) 21 and exposure of the F_1 generation started on PND 22. Estimates of internal exposure were determined by measuring EB concentrations in blood collected from F_1 dams (4/group) and their culled pups 1 hr after the last gavage dose on PND 4. On PND 22, blood was collected from these same F_1 dams and their weanlings for EB analysis 1 h after a 6-h inhalation exposure. The remainder of the F_2 generation was not directly exposed.

Limitations: This study was not specifically designed to investigate an endocrine mode of action. Although a negative result supports the conclusion that the response is not indicative of the hypothesis, a positive result could be due to several mechanisms.

Results included in WOE: The mean body weight gain of males and females in the F_1 and F_2 offspring, between postnatal days 1-4, did not differ significantly from the control animals.

5.5 Rank 2: Reproductive Toxicity – Pup survival

[2] ***Faber and colleagues (2006)*** Four groups of Crl:CD(SD)IGS BR rats (30/sex/group for F_0 and 25/sex/group for F_1) were exposed to 0, 25, 100, and 500ppm EB for 6 h/day for at least 70 consecutive days before mating. Inhalation exposure for the F_0 and F_1 females continued throughout mating, gestation through gestation day (GD) 20, and lactation days (LD) 5–21. On LD 1–4, females received EB in corn oil via oral gavage at dose levels of 26, 90, and 342 mg/kg/day (divided into three equal doses, approximately 2 h apart). Pups were weaned on postnatal day (PND) 21 and exposure of the F_1 generation started on PND 22. Estimates of internal exposure were determined by measuring EB concentrations in blood collected from F_1 dams (4/group) and their culled pups 1 hr after the last gavage dose on PND 4. On PND 22, blood was collected from these same F_1 dams and their weanlings for EB analysis 1 h after a 6-hinhalation exposure. The remainder of the F_2 generation was not directly exposed.

Limitations: This study was not specifically designed to investigate an endocrine mode of action. Although a negative result supports the conclusion that the response is not indicative of the hypothesis, a positive result could be due to several mechanisms.

Results included in WOE: The percentage of F_1 and F_2 pups surviving from birth to PND 4 and from PND 4-21 did not differ between treatment animals and controls.

5.6 Rank 2: Reproductive Toxicity – Thyroid weight

[2] ***Faber and colleagues (2006)*** Four groups of Crl:CD(SD)IGS BR rats (30/sex/group for F_0 and 25/sex/group for F_1) were exposed to 0, 25, 100, and 500ppm EB for 6 h/day for at least 70 consecutive days before mating. Inhalation exposure for the F_0 and F_1 females continued throughout mating, gestation through gestation day (GD) 20, and lactation days (LD) 5–21. On LD 1–4, females received EB in corn oil via oral gavage at dose levels of 26, 90, and 342 mg/kg/day (divided into three equal doses, approximately 2 h apart). Pups were weaned on postnatal day (PND) 21 and exposure of the F_1 generation started on PND 22. Estimates of internal exposure were determined by measuring EB concentrations in blood collected from F_1 dams (4/group) and their culled pups 1 h after the last gavage dose on PND 4. On PND 22, blood was collected from these same F_1 dams and their weanlings for EB analysis 1 h after a 6-h inhalation exposure. The remainder of the F_2 generation was not directly exposed.

Limitations: This study was not specifically designed to investigate an endocrine mode of action. Although a negative result supports the conclusion that the response is not indicative of the hypothesis, a positive result could be due to several mechanisms. The authors note that because this finding was only found in the F_1 males and not in the F_1 females or the F_2 treatment animals that the finding was considered to be the result of normal biological variation and not related to ethylbenzene exposure. However, we could not ascertain that a histopathologic examination of the thyroid tissue was carried out to rule out pathologic changes.

Results included in WOE: Increases (approximately 18–20% and statistically significant) in absolute and relative thyroid weights in the F_0 males in the 100 and 500 ppm groups were not replicated in the F_1 male group nor were they observed in the female groups exposed to these concentrations of ethylbenzene.

5.7 Rank 3: Repeat Dose Toxicity – Liver weight

[3] ***Mellert and colleagues (2007)*** Ethylbenzene was administered to groups of male and female Wistar rats by gavage for 4 (n = 5/ dose/sex) and 13 weeks (n = 10/dose/sex) (OECD 408) at doses of 0 (vehicle control), 75, 250, and 750 mg/kg bw/day, administered am/pm as half doses.

Limitations: This study was not specifically designed to investigate an endocrine mode of action. Although a negative result supports the conclusion that the response is not indicative of the hypothesis, a positive result could be due to several mechanisms. However, the histological examination of liver tissue demonstrated only centrilobular hypertrophy of hepatocytes suggesting an adaptive response.

Results included in WOE: Liver weight was increased in a dose-related fashion in both male and female rats in the mid and high dose exposure groups.

[6] ***Saillenfait and colleagues (2006)*** Pregnant Sprague–Dawley rats were exposed to ethylbenzene (EB; 0, 250, or 1000 ppm) and methylethylketone (MEK; 0, 1000, or 3000 ppm), alone and in combination, by inhalation, for 6 h/day, during days 6–20 of gestation.

Limitations: This study was not specifically designed to investigate an endocrine mode of action. Although a negative result supports the conclusion that the response is not indicative of the hypothesis, a positive result could be due to several mechanisms. High doses were used, and it is unclear if the endpoint responses are secondary to general toxicity. Additionally, histological evaluation of the liver revealed no pathological effects attributable to solvent exposures therefore we agree with the authors that the positive liver weight changes are likely an adaptive response.

Results included in WOE: Compared with control, both absolute and relative liver weight were significantly elevated in animals treated with 250 and 1000 ppm ethylbenzene.

[7] Li and colleagues (2010) In the neurotoxicity study, ethylbenzene was administered orally via gavage twice daily to Sprague-Dawley male and female rats at 0, 25, 125, or 250 mg/kg per dose (total daily dosages of 0, 50, 250, or 500 mg/kg bw/day) for 13 weeks and the functional observational battery (FOB), automated tests for motor activity and neuropathological examination were conducted.

Limitations: This study was not specifically designed to investigate an endocrine mode of action. Although a negative result supports the conclusion that the response is not indicative of the hypothesis, a positive result could be due to several mechanisms. In addition, there were no treatment-related microscopic lesions observed in the liver suggesting the increased relative liver weight finding represents an adaptive response rather than a pathological change.

Results included in WOE: The weights of the liver relative to terminal body weights were significantly increased ($p \leq 0.05$) in male rats at 250 and 500 mg/kg bw/day and in female rats at 500 mg/kg bw/day.

[8] ***Stott and colleagues (1999)*** Male and female Fischer 344 rats and B6C3F1 mice were exposed to 0 or 750 ppm ethylbenzene vapor 6 h/day for one or four weeks. Livers from 6 (one-week study) or 8 (four-week study) mice/sex/dose were examined and weighed.

Limitations: This study was not specifically designed to investigate an endocrine mode of action. Although a negative result supports the conclusion that the response is not indicative of the hypothesis,

a positive result could be due to several mechanisms. In addition, increased liver weights were not accompanied by histological changes suggesting an adaptive rather than a pathologic response.

Results included in WOE: The relative liver weight of male and female mice exposed to 750 ppm EB for one week and female mice exposed to 750 ppm for four weeks were significantly higher than those of control animals. There were no significant differences in liver weight between male mice and male controls in the four-week study.

[9] ***NTP (National Toxicology Program). Toxicity studies of ethylbenzene (CAS No. 100-41-4) in F344/N rats and B6C3F1 mice (inhalation studies) (1992)*** Inhalation toxicity of ethylbenzene was studied by exposing groups of seven-week-old F344/N rats and B6C3Fi mice of each sex to ethylbenzene vapor at chamber concentrations of 0, 100, 250, 500, 750, or 1000 ppm, 6 h per day, 5 days per week for 13 weeks.

Limitations: This study was not specifically designed to investigate an endocrine mode of action. Although a negative result supports the conclusion that the response is not indicative of the hypothesis, a positive result could be due to several mechanisms. Additionally, high doses were used. It is unclear if the endpoint responses are secondary to general toxicity. There were no treatment-related microscopic lesions associated with the increased liver weights, therefore this change is considered to be an adaptive response rather than a pathological finding.

Results included in WOE: Significant increases in liver weights were seen in male rats in the 250-, 500-, 750- and 1000 ppm exposure groups and in female rats in the 500-, 750- and 1000 ppm exposure groups; significant increases in liver weights were seen in male and female mice in the 750- and 1000-ppm exposure groups.

[10] Cragg and colleagues (1989) Mice, rats and rabbits (five/sex/group) were exposed by inhalation to ethylbenzene vapors for 6 h/day, 5 days/week for 4 weeks (20 exposures). Rats and mice received 0, 99, 382 or 782 ppm EB while rabbits received 0, 382, 782 or 1610 ppm.

Limitations: This study was not specifically designed to investigate an endocrine mode of action. Although a negative result supports the conclusion that the response is not indicative of the hypothesis, a positive result could be due to several mechanisms.

Results included in WOE: There were statistically significant increases in the liver/body weight ratios of male rats in the 782 ppm dose group and female rats in the 382 ppm and 782 ppm dose groups. Absolute liver weight was increased for female rats at 782 ppm and for male rats at 382 and 782 ppm. The absolute liver weight for female mice was also significantly increased at 782 ppm. We agree with the authors note that the absence of accompanying liver histopathology or abnormal clinical chemistry indicates that the increases were due to an adaptive induction of hepatic function rather than toxicity. Liver weights were unchanged in rabbits exposed to EB at any concentrations up to 1610 ppm.

5.8 Rank 3: Reproductive Toxicity – Liver weight

[2] ***Faber and colleagues (2006)*** Four groups of Crl:CD(SD)IGS BR rats (30/sex/group for F_0 and 25/sex/group for F_1) were exposed to 0, 25, 100, and 500ppm EB for 6 h/day for at least 70 consecutive days before mating. Inhalation exposure for the F_0 and F_1 females continued throughout mating, gestation through gestation day (GD) 20, and lactation days (LD) 5–21. On LD 1–4, females received EB in corn oil via oral gavage at dose levels of 26, 90, and 342 mg/kg/day (divided into three equal doses, approximately 2 h apart). Pups were weaned on postnatal day (PND) 21 and exposure of

the F_1 generation started on PND 22. Estimates of internal exposure were determined by measuring EB concentrations in blood collected from F_1 dams (4/group) and their culled pups 1 h after the last gavage dose on PND 4. On PND 22, blood was collected from these same F_1 dams and their weanlings for EB analysis 1 h after a 6-h inhalation exposure. The remainder of the F_2 generation was not directly exposed.

Limitations: This study was not specifically designed to investigate an endocrine mode of action. Although a negative result supports the conclusion that the response is not indicative of the hypothesis, a positive result could be due to several mechanisms. Additionally, histological evaluation of the liver revealed no pathological effects attributable to solvent exposures therefore we agree with the authors that the positive liver weight changes are likely an adaptive response.

Results included in WOE: Absolute and relative liver weights were slightly increased (3–7%) in the 500ppm groups compared to the control group. The increases in relative liver weight were statistically significant in the F_0 and F_1 females. These increases in the liver weights were considered related to ethylbenzene exposure but not a pathological finding.

5.9 Rank 3: Developmental Neurotoxicity – Auditory startle

[12] ***Faber and colleagues (2007)*** Four groups of Crl:CD(SD)IGS BR rats (30/sex/group for F_0 and 25/sex/group for F_1) were exposed to 0, 25, 100, and 500 ppm EB for 6 h/day for at least 70 consecutive days before mating. Inhalation exposure for the F_0 and F_1 females continued throughout mating, gestation through gestation day (GD) 20, and lactation days (LD) 5–21. On LD 1–4, females received EB in corn oil via oral gavage at dose levels of 26, 90, and 342 mg/kg/day (divided into three equal doses, approximately 2 h apart). Pups were weaned on postnatal day (PND) 21 and exposure of the F_1 generation started on PND 22. Estimates of internal exposure were determined by measuring EB concentrations in blood collected from F_1 dams (4/group) and their culled pups 1 hr after the last gavage dose on PND 4. On PND 22, blood was collected from these same F_1 dams and their weanlings for EB analysis 1 h after a 6-h inhalation exposure. The remainder of the F_2 generation was not directly exposed. Neurobehavioral development of one F2-generation treatment derived offspring/sex/litter was assessed in a functional observational battery (FOB; PND 4, 11, 22, 45, and 60), motor activity sessions (PND 13, 17, 21, and 61), acoustic startle testing (PND 20 and 60), a Biel water maze learning and memory task (initiated on PND 26 or 62), and in evaluations of whole-brain measurements and brain morphometric and histologic assessments (PND 21 and 72).

Limitations: This study was not specifically designed to investigate an endocrine mode of action. Although a negative result supports the conclusion that the response is not indicative of the hypothesis, a positive result could be due to several mechanisms.

Results included in WOE: There were no statistically significant differences in parameter of the acoustic startle test for the F_2 treatment derived offspring of either gender. On PND 20, the maximum startle amplitudes in the control group were much lower than the mean historical control values for this assessment age. Also on PND 60, a statistically significant main effect of treatment was obtained for maximum startle amplitude in the F_2 males, however there was an outlier response of three control males which inflated the mean. When these responses were removed, the within group distributions were not markedly different and closely matched historical control values. Therefore, the differences noted in males at this age were attributed to unusual control mean values and were not considered to be related to parental ethylbenzene exposure.

5.10 Rank 3: Developmental Neurotoxicity – Brain morphometry

[12] ***Faber and colleagues (2007)*** Four groups of Crl:CD(SD)IGS BR rats (30/sex/group for F_0 and 25/sex/group for F_1) were exposed to 0, 25, 100, and 500 ppm EB for 6 h/day for at least 70 consecutive days before mating. Inhalation exposure for the F_0 and F_1 females continued throughout mating, gestation through gestation day (GD) 20, and lactation days (LD) 5–21. On LD 1–4, females received EB in corn oil via oral gavage at dose levels of 26, 90, and 342 mg/kg/day (divided into three equal doses, approximately 2 h apart). Pups were weaned on postnatal day (PND) 21 and exposure of the F_1 generation started on PND 22. Estimates of internal exposure were determined by measuring EB concentrations in blood collected from F_1 dams (4/group) and their culled pups 1 hr after the last gavage dose on PND 4. On PND 22, blood was collected from these same F_1 dams and their weanlings for EB analysis 1 h after a 6-h inhalation exposure. The remainder of the F_2 generation was not directly exposed. Neurobehavioral development of one F2-generation treatment derived offspring/sex/litter was assessed in a functional observational battery (FOB; PND 4, 11, 22, 45, and 60), motor activity sessions (PND 13, 17, 21, and 61), acoustic startle testing (PND 20 and 60), a Biel water maze learning and memory task (initiated on PND 26 or 62), and in evaluations of whole-brain measurements and brain morphometric and histologic assessments (PND 21 and 72).

Limitations: This study was not specifically designed to investigate an endocrine mode of action. Although a negative result supports the conclusion that the response is not indicative of the hypothesis, a positive result could be due to several mechanisms.

Results included in WOE: No brain morphometric changes were noted at either age (PNDs 21, 72) in the measurements taken in the height of the hemisphere and vertical thickness of the cortex, the radial thickness of the cortex, vertical heights between hippocampal pyramidal neuron layers, vertical height of the dentate hilus, the length of the ventral limb of the dentate hilus or the vertical thickness of the brainstem and base of cerebellar lobule 9, in animals of either gender.

5.11 Rank 3: Developmental Neurotoxicity – Learning and memory

[12] ***Faber and colleagues (2007)*** Four groups of Crl:CD(SD)IGS BR rats (30/sex/group for F_0 and 25/sex/group for F_1) were exposed to 0, 25, 100, and 500 ppm EB for 6 h/day for at least 70 consecutive days before mating. Inhalation exposure for the F_0 and F_1 females continued throughout mating, gestation through gestation day (GD) 20, and lactation days (LD) 5–21. On LD 1–4, females received EB in corn oil via oral gavage at dose levels of 26, 90, and 342 mg/kg/day (divided into three equal doses, approximately 2 h apart). Pups were weaned on postnatal day (PND) 21 and exposure of the F_1 generation started on PND 22. Estimates of internal exposure were determined by measuring EB concentrations in blood collected from F_1 dams (4/group) and their culled pups 1 hr after the last gavage dose on PND 4. On PND 22, blood was collected from these same F_1 dams and their weanlings for EB analysis 1 h after a 6-h inhalation exposure. The remainder of the F_2 generation was not directly exposed. Neurobehavioral development of one F2-generation treatment derived offspring/sex/litter was assessed in a functional observational battery (FOB; PND 4, 11, 22, 45, and 60), motor activity sessions (PND 13, 17, 21, and 61), acoustic startle testing (PND 20 and 60), a Biel water maze learning and memory task (initiated on PND 26 or 62), and in evaluations of whole-brain measurements and brain morphometric and histologic assessments (PND 21 and 72).

Limitations: This study was not specifically designed to investigate an endocrine mode of action. Although a negative result supports the conclusion that the response is not indicative of the hypothesis, a positive result could be due to several mechanisms.

Results included in WOE: Assessments of straight-alley escape times and aspects of learning and memory in the Biel water maze task were initiated on PND 26 and PND 62. There were no biologically meaningful differences noted in animals of either gender at either testing age.

5.12 Rank 3: Developmental Neurotoxicity – Motor activity

[12] ***Faber and colleagues (2007)*** Four groups of Crl:CD(SD)IGS BR rats (30/sex/group for F_0 and 25/sex/group for F_1) were exposed to 0, 25, 100, and 500 ppm EB for 6 h/day for at least 70 consecutive days before mating. Inhalation exposure for the F_0 and F_1 females continued throughout mating, gestation through gestation day (GD) 20, and lactation days (LD) 5–21. On LD 1–4, females received EB in corn oil via oral gavage at dose levels of 26, 90, and 342 mg/kg/day (divided into three equal doses, approximately 2 h apart). Pups were weaned on postnatal day (PND) 21 and exposure of the F_1 generation started on PND 22. Estimates of internal exposure were determined by measuring EB concentrations in blood collected from F_1 dams (4/group) and their culled pups 1 h after the last gavage dose on PND 4. On PND 22, blood was collected from these same F_1 dams and their weanlings for EB analysis 1 h after a 6-h inhalation exposure. The remainder of the F_2 generation was not directly exposed. Neurobehavioral development of one F2-generation treatment derived offspring/sex/litter was assessed in a functional observational battery (FOB; PND 4, 11, 22, 45, and 60), motor activity sessions (PND 13, 17, 21, and 61), acoustic startle testing (PND 20 and 60), a Biel water maze learning and memory task (initiated on PND 26 or 62), and in evaluations of whole-brain measurements and brain morphometric and histologic assessments (PND 21 and 72).

Limitations: This study was not specifically designed to investigate an endocrine mode of action. Although a negative result supports the conclusion that the response is not indicative of the hypothesis, a positive result could be due to several mechanisms.

Results included in WOE: There was no statistically significant differences among the groups in cumulative session activity counts during the pre-weaning period (PND13, 17, 21). There was a significant main effect of treatment found in the repeated measure of analysis of variance for total activity counts for females on PND 61, but due to the relatively slight change in this behavior and the lack of any suggested dose-response relationship in either gender, this difference was not considered to be related to parental ethylbenzene exposure.

6. Interaction with Steroidogenesis Enzymes Hypothesis

6.1 Rank 2: Repeat Dose Toxicity – Ovary histopathology

[1] ***NTP (National Toxicology Program). (1999)*** Groups of 50 male and 50 female Fischer rats and B6C3F1 mice were exposed to ethylbenzene by inhalation at 0, 75, 250, and 750 ppm 6 h per day, 5 days per week, for 104 weeks (rats) and for 103 week (mice).

Limitations: This study was not specifically designed to investigate an endocrine mode of action. Although a negative result supports the conclusion that the response is not indicative of the hypothesis, a positive result could be due to several mechanisms.

Results included in WOE: There were no significant histological differences between the chamber control animals and treatment group animals, in either specie, for uterine, vaginal or ovarian tissues in females or for testes, prostate, epididymides or seminal vesicles for males.

[3] ***Mellert and colleagues (2007)*** Ethylbenzene was administered to groups of male and female Wistar rats by gavage for 4 (*n* = 5/ dose/sex) and 13 weeks (*n* = 10/dose/sex) (OECD 408) at doses of 0 (vehicle control), 75, 250, and 750 mg/kg bw/day, administered am/pm as half doses.

Limitations: This study was not specifically designed to investigate an endocrine mode of action. Although a negative result supports the conclusion that the response is not indicative of the hypothesis, a positive result could be due to several mechanisms.

Results included in WOE: Histopathological evaluation of the epididymis, mammary glands, ovaries, prostate, seminal vesicles, testes, thyroid, uterus, and vagina did not identify any adverse effects.

[9] ***(National Toxicology Program). Toxicity studies of ethylbenzene (CAS No. 100-41-4) in F344/N rats and B6C3F1 mice (inhalation studies) (1992)*** Inhalation toxicity of ethylbenzene was studied by exposing groups of seven-week-old F344/N rats and B6C3Fi mice of each sex to ethylbenzene vapor at chamber concentrations of 0, 100, 250, 500, 750, or 1000 ppm, 6 h per day, 5 days per week for 13 weeks.

Limitations: This study was not specifically designed to investigate an endocrine mode of action. Although a negative result supports the conclusion that the response is not indicative of the hypothesis, a positive result could be due to several mechanisms.

Results included in WOE: There were no histopathologic lesions and no chemically related histopathologic changes identified in the ovaries of mice or rats compared with controls.

[10] Cragg and colleagues (1989) Mice, rats and rabbits (five/sex/group) were exposed by inhalation to ethylbenzene vapors for 6 h/day, 5 days/week for 4 weeks (20 exposures). Rats and mice received 0, 99, 382 or 782 ppm EB while rabbits received 0, 382, 782 or 1610 ppm.

Limitations: This study was not specifically designed to investigate an endocrine mode of action. Although a negative result supports the conclusion that the response is not indicative of the hypothesis, a positive result could be due to several mechanisms.

Results included in WOE: The gonads (ovaries or testes with epididymides) and thyroids of high-exposure and controls animals of all species were subjected to histopathological examination. There were no gross or microscopic changes attributable to ethylbenzene in any of these tissues.

6.2 Rank 2: Repeat Dose toxicity – Testis histopathology

[1] ***NTP (National Toxicology Program). (1999)*** Groups of 50 male and 50 female Fischer rats and B6C3F1 mice were exposed to ethylbenzene by inhalation at 0, 75, 250, and 750 ppm 6 h per day, 5 days per week, for 104 weeks (rats) and for 103 week (mice).

Limitations: This study was not specifically designed to investigate an endocrine mode of action. Although a negative result supports the conclusion that the response is not indicative of the hypothesis, a positive result could be due to several mechanisms.

Results included in WOE: There were no significant histological differences between the chamber control animals and treatment group animals, in either specie, for uterine, vaginal or ovarian tissues in

females or for testes, prostate, epididymides or seminal vesicles for males.

[3] ***Mellert and colleagues (2007)*** Ethylbenzene was administered to groups of male and female Wistar rats by gavage for 4 (*n* = 5/ dose/sex) and 13 weeks (*n* = 10/dose/sex) (OECD 408) at doses of 0 (vehicle control), 75, 250, and 750 mg/kg bw/day, administered am/pm as half doses.

Limitations: This study was not specifically designed to investigate an endocrine mode of action. Although a negative result supports the conclusion that the response is not indicative of the hypothesis, a positive result could be due to several mechanisms.

Results included in WOE: Histopathological evaluation of the epididymis, mammary glands, ovaries, prostate, seminal vesicles, testes, thyroid, uterus, and vagina did not identify any adverse effects.

[9] ***NTP (National Toxicology Program). Toxicity studies of ethylbenzene (CAS No. 100-41-4) in F344/N rats and B6C3F1 mice (inhalation studies) (1992)*** Inhalation toxicity of ethylbenzene was studied by exposing groups of seven-week-old F344/N rats and B6C3Fi mice of each sex to ethylbenzene vapor at chamber concentrations of 0, 100, 250, 500, 750, or 1000 ppm, 6 h per day, 5 days per week for 13 weeks.

Limitations: This study was not specifically designed to investigate an endocrine mode of action. Although a negative result supports the conclusion that the response is not indicative of the hypothesis, a positive result could be due to several mechanisms.

Results included in WOE: There were no histopathologic lesions and no chemically related histopathologic changes identified in the testes of mice or rats compared with controls.

[10] Cragg and colleagues (1989) Mice, rats and rabbits (five/sex/group) were exposed by inhalation to ethylbenzene vapors for 6 h/day, 5 days/week for 4 weeks (20 exposures). Rats and mice received 0, 99, 382 or 782 ppm EB while rabbits received 0, 382, 782 or 1610 ppm.

Limitations: This study was not specifically designed to investigate an endocrine mode of action. Although a negative result supports the conclusion that the response is not indicative of the hypothesis, a positive result could be due to several mechanisms.

Results included in WOE: The gonads (ovaries or testes with epididymides) and thyroids of high-exposure and controls animals of all species were subjected to histopathological examination. There were no gross or microscopic changes attributable to ethylbenzene in any of these tissues.

6.3 Rank 2: Repeat Dose Toxicity – Uterus histopathology

[1] ***NTP (National Toxicology Program). (1999)*** Groups of 50 male and 50 female Fischer rats and B6C3F1 mice were exposed to ethylbenzene by inhalation at 0, 75, 250, and 750 ppm 6 h per day, 5 days per week, for 104 weeks (rats) and for 103 week (mice).

Limitations: This study was not specifically designed to investigate an endocrine mode of action. Although a negative result supports the conclusion that the response is not indicative of the hypothesis, a positive result could be due to several mechanisms.

Results included in WOE: There were no significant histological differences between the chamber

control animals and treatment group animals, in either specie, for uterine, vaginal or ovarian tissues in females or for testes, prostate, epididymides or seminal vesicles for males.

[3] ***Mellert and colleagues (2007)*** Ethylbenzene was administered to groups of male and female Wistar rats by gavage for 4 (*n* = 5/ dose/sex) and 13 weeks (*n* = 10/dose/sex) (OECD 408) at doses of 0 (vehicle control), 75, 250, and 750 mg/kg bw/day, administered am/pm as half doses.

Limitations: This study was not specifically designed to investigate an endocrine mode of action. Although a negative result supports the conclusion that the response is not indicative of the hypothesis, a positive result could be due to several mechanisms.

Results included in WOE: Histopathological evaluation of the epididymis, mammary glands, ovaries, prostate, seminal vesicles, testes, thyroid, uterus, and vagina did not identify any adverse effects.

[9] ***NTP (National Toxicology Program). Toxicity studies of ethylbenzene (CAS No. 100-41-4) in F344/N rats and B6C3F1 mice (inhalation studies) (1992)*** Inhalation toxicity of ethylbenzene was studied by exposing groups of seven-week-old F344/N rats and B6C3Fi mice of each sex to ethylbenzene vapor at chamber concentrations of 0, 100, 250, 500, 750, or 1000 ppm, 6 h per day, 5 days per week for 13 weeks.

Limitations: This study was not specifically designed to investigate an endocrine mode of action. Although a negative result supports the conclusion that the response is not indicative of the hypothesis, a positive result could be due to several mechanisms.

Results included in WOE: There were no histopathologic lesions and no chemically related histopathologic changes identified in the uteruses of mice or rats compared with controls.

[10] Cragg and colleagues (1989) Mice, rats and rabbits (five/sex/group) were exposed by inhalation to ethylbenzene vapors for 6 h/day, 5 days/week for 4 weeks (20 exposures). Rats and mice received 0, 99, 382 or 782 ppm EB while rabbits received 0, 382, 782 or 1610 ppm.

Limitations: This study was not specifically designed to investigate an endocrine mode of action. Although a negative result supports the conclusion that the response is not indicative of the hypothesis, a positive result could be due to several mechanisms.

Results included in WOE: The uterus of high-exposure and controls animals of all species was subjected to histopathological examination. There were no gross or microscopic changes attributable to ethylbenzene in this organ.

6.4 Rank 2: Developmental Toxicity – Sex ratio

[5] ***Saillenfait and colleagues (2003)*** The developmental toxicity of ethylbenzene was studied in Sprague–Dawley rats after inhalation exposure. Animals were exposed to ethylbenzene at 100, 500, 1000 or 2000 ppm, for 6 h/day, during days 6–20 of gestation.

Limitations: This study was not specifically designed to investigate an endocrine mode of action. Although a negative result supports the conclusion that the response is not indicative of the hypothesis, a positive result could be due to several mechanisms.

Results included in WOE: The percentage of males per litter did not differ between any of the treatments groups and controls.

[13] ***Andrew et al., (1981); Hardin et al. (1981)*** Groups of 29-33 Female Wistar rats and New Zealand White rabbits were exposed to 0, 100, or 1000 ppm ethylbenzene for 7 h/day, 5 days/week for 3 weeks, then mated with unexposed males. Pregnant females were further exposed to 0, 100, or 1000 ppm 7 h/day through Gestational Day 19 (rats) and 24 (rabbits).

Limitations: This study was not specifically designed to investigate an endocrine mode of action. Although a negative result supports the conclusion that the response is not indicative of the hypothesis, a positive result could be due to several mechanisms.

Results included in the WOE: Sex ratio in rats and rabbits was unaffected by exposure to 100 or to 1,000 ppm ethylbenzene relative to unexposed controls.

6.5 Rank 2: Reproductive Toxicity – Estrous cyclicity

[2] ***Faber and colleagues (2006)*** Four groups of Crl:CD(SD)IGS BR rats (30/sex/group for F_0 and 25/sex/group for F_1) were exposed to 0, 25, 100, and 500ppm EB for 6 h/day for at least 70 consecutive days before mating. Inhalation exposure for the F_0 and F_1 females continued throughout mating, gestation through gestation day (GD) 20, and lactation days (LD) 5–21. On LD 1–4, females received EB in corn oil via oral gavage at dose levels of 26, 90, and 342 mg/kg/day (divided into three equal doses, approximately 2 h apart). Pups were weaned on postnatal day (PND) 21 and exposure of the F_1 generation started on PND 22. Estimates of internal exposure were determined by measuring EB concentrations in blood collected from F_1 dams (4/group) and their culled pups 1 hr after the last gavage dose on PND 4. On PND 22, blood was collected from these same F_1 dams and their weanlings for EB analysis 1 h after a 6-h inhalation exposure. The remainder of the F_2 generation was not directly exposed.

Limitations: This study was not specifically designed to investigate an endocrine mode of action. Although a negative result supports the conclusion that the response is not indicative of the hypothesis, a positive result could be due to several mechanisms.

Results included in WOE: The mean estrous cycle length (4.0 ± 0.3days) was significantly reduced for the F_0, 500ppm group when compared to the F_0 control group value (4.4 ± 0.8 days). However, the authors felt this difference was not biologically important because all females in this group were cycling normally and this strain of rat normally exhibits 4-5 day estrous cycles. Mean estrous cycle length did not differ between control and experimental F_1 offspring.

6.6 Rank 2: Reproductive Toxicity – Fertility

[2] ***Faber and colleagues (2006)*** Four groups of Crl:CD(SD)IGS BR rats (30/sex/group for F_0 and 25/sex/group for F_1) were exposed to 0, 25, 100, and 500ppm EB for 6 h/day for at least 70 consecutive days before mating. Inhalation exposure for the F_0 and F_1 females continued throughout mating, gestation through gestation day (GD) 20, and lactation days (LD) 5–21. On LD 1–4, females received EB in corn oil via oral gavage at dose levels of 26, 90, and 342 mg/kg/day (divided into three equal doses, approximately 2 h apart). Pups were weaned on postnatal day (PND) 21 and exposure of the F_1 generation started on PND 22. Estimates of internal exposure were determined by measuring EB concentrations in blood collected from F_1 dams (4/group) and their culled pups 1 hr after the last

gavage dose on PND 4. On PND 22, blood was collected from these same F_1 dams and their weanlings for EB analysis 1 h after a 6-h inhalation exposure. The remainder of the F_2 generation was not directly exposed.

Limitations: This study was not specifically designed to investigate an endocrine mode of action. Although a negative result supports the conclusion that the response is not indicative of the hypothesis, a positive result could be due to several mechanisms.

Results included in WOE: There was no impairment of fertility or increased time to mating in the F_0 or F_1 offspring.

6.7 Rank 2: Reproductive Toxicity – Live births

[2] ***Faber and colleagues (2006)*** Four groups of Crl:CD(SD)IGS BR rats (30/sex/group for F_0 and 25/sex/group for F_1) were exposed to 0, 25, 100, and 500ppm EB for 6 h/day for at least 70 consecutive days before mating. Inhalation exposure for the F_0 and F_1 females continued throughout mating, gestation through gestation day (GD) 20, and lactation days (LD) 5–21. On LD 1–4, females received EB in corn oil via oral gavage at dose levels of 26, 90, and 342 mg/kg/day (divided into three equal doses, approximately 2 h apart). Pups were weaned on postnatal day (PND) 21 and exposure of the F_1 generation started on PND 22. Estimates of internal exposure were determined by measuring EB concentrations in blood collected from F_1 dams (4/group) and their culled pups 1 hr after the last gavage dose on PND 4. On PND 22, blood was collected from these same F_1 dams and their weanlings for EB analysis 1 h after a 6-h inhalation exposure. The remainder of the F_2 generation was not directly exposed.

Limitations: This study was not specifically designed to investigate an endocrine mode of action. Although a negative result supports the conclusion that the response is not indicative of the hypothesis, a positive result could be due to several mechanisms.

Results included in WOE: The mean number of F_1 and F_2 pups born, live litter size, percentage of males per litter at birth, and postnatal survival were unaffected by ethylbenzene exposure.

6.8 Rank 2: Reproductive Toxicity – Mating index

[2] ***Faber and colleagues (2006)*** Four groups of Crl:CD(SD)IGS BR rats (30/sex/group for F_0 and 25/sex/group for F_1) were exposed to 0, 25, 100, and 500ppm EB for 6 h/day for at least 70 consecutive days before mating. Inhalation exposure for the F_0 and F_1 females continued throughout mating, gestation through gestation day (GD) 20, and lactation days (LD) 5–21. On LD 1–4, females received EB in corn oil via oral gavage at dose levels of 26, 90, and 342 mg/kg/day (divided into three equal doses, approximately 2 h apart). Pups were weaned on postnatal day (PND) 21 and exposure of the F_1 generation started on PND 22. Estimates of internal exposure were determined by measuring EB concentrations in blood collected from F_1 dams (4/group) and their culled pups 1 h after the last gavage dose on PND 4. On PND 22, blood was collected from these same F_1 dams and their weanlings for EB analysis 1 h after a 6-h inhalation exposure. The remainder of the F_2 generation was not directly exposed.

Limitations: This study was not specifically designed to investigate an endocrine mode of action. Although a negative result supports the conclusion that the response is not indicative of the hypothesis, a positive result could be due to several mechanisms.

Results included in WOE: The male and female mating indices (%) were not different between the any of the treatment animals and controls.

6.9 Rank 2: Reproductive Toxicity – Sex ratio

[2] ***Faber and colleagues (2006)*** Four groups of Crl:CD(SD)IGS BR rats (30/sex/group for F_0 and 25/sex/group for F_1) were exposed to 0, 25, 100, and 500ppm EB for 6 h/day for at least 70 consecutive days before mating. Inhalation exposure for the F_0 and F_1 females continued throughout mating, gestation through gestation day (GD) 20, and lactation days (LD) 5–21. On LD 1–4, females received EB in corn oil via oral gavage at dose levels of 26, 90, and 342 mg/kg/day (divided into three equal doses, approximately 2 h apart). Pups were weaned on postnatal day (PND) 21 and exposure of the F_1 generation started on PND 22. Estimates of internal exposure were determined by measuring EB concentrations in blood collected from F_1 dams (4/group) and their culled pups 1 hr after the last gavage dose on PND 4. On PND 22, blood was collected from these same F_1 dams and their weanlings for EB analysis 1 h after a 6-h inhalation exposure. The remainder of the F_2 generation was not directly exposed.

Limitations: This study was not specifically designed to investigate an endocrine mode of action. Although a negative result supports the conclusion that the response is not indicative of the hypothesis, a positive result could be due to several mechanisms.

Results included in WOE: The sex distribution, measured by the % males/litter, was not different in either F_1 or F_2 litters compared with control litters.

6.10 Rank 2: Reproductive Toxicity –Sperm count

[2] ***Faber and colleagues (2006)*** Four groups of Crl:CD(SD)IGS BR rats (30/sex/group for F_0 and 25/sex/group for F_1) were exposed to 0, 25, 100, and 500ppm EB for 6 h/day for at least 70 consecutive days before mating. Inhalation exposure for the F_0 and F_1 females continued throughout mating, gestation through gestation day (GD) 20, and lactation days (LD) 5–21. On LD 1–4, females received EB in corn oil via oral gavage at dose levels of 26, 90, and 342 mg/kg/day (divided into three equal doses, approximately 2 h apart). Pups were weaned on postnatal day (PND) 21 and exposure of the F_1 generation started on PND 22. Estimates of internal exposure were determined by measuring EB concentrations in blood collected from F_1 dams (4/group) and their culled pups 1 h after the last gavage dose on PND 4. On PND 22, blood was collected from these same F_1 dams and their weanlings for EB analysis 1 h after a 6-h inhalation exposure. The remainder of the F_2 generation was not directly exposed.

Limitations: This study was not specifically designed to investigate an endocrine mode of action. Although a negative result supports the conclusion that the response is not indicative of the hypothesis, a positive result could be due to several mechanisms.

Results included in WOE: The mean sperm number (millions/g tissue) in the left cauda epididymis for F_0 and F_1 males were not different between any treatment group and controls.

6.11 Rank 3: Repeat Dose Toxicity – Gross Pathology

[3] ***Mellert and colleagues (2007)*** Ethylbenzene was administered to groups of male and female Wistar rats by gavage for 4 (n = 5/ dose/sex) and 13 weeks (n = 10/dose/sex) (OECD 408) at doses of 0 (vehicle control), 75, 250, and 750 mg/ kg bodyweight/day (mg/kg bw/day), administered am/pm as half doses.

Limitations: This study was not specifically designed to investigate an endocrine mode of action. Although a negative result supports the conclusion that the response is not indicative of the hypothesis, a positive result could be due to several mechanisms.

Results included in WOE: Gross pathological evaluation of the epididymis, mammary glands, ovaries, prostate, seminal vesicles, testes, thyroid, uterus, and vagina did not identify any adverse effects.

Supplemental Material C
Rationale for Excluding Studies

Gong et al., 2023.
Gong, X., Huang, Y., Duong, J., Leng, S., Zhan, F.B., Guo, Y., Lin, Y., Luo, L. 2023. Industrial air pollution and low birth weight in New Mexico, USA. Journal of Environmental Management, 348, part. no. 119236, DOI: 10.1016/j.jenvman.2023.119236.

This study evaluated the relationship between exposure to air pollution and Low Birth Weight (LBW) among 22,375 LBW cases and 233,340 controls in New Mexico, where the incidence of LBW has exceeded the national average in recent decades. Exposure focused on 14 common chemicals listed in the Toxic Release Inventory (TRI) and monitoring datasets, which have abundant monitoring samples. The Emission Weighted Proximity Model (EWPM) was used to calculate maternal air pollution exposure intensity. Adjusted odds ratios (adjORs) were calculated using binary logistic regressions to examine the association between maternal residential air pollution exposure and LBW, while controlling for potential confounders, such as the maternal age, race/ethnicity, gestational age, prenatal care, education level, consumption of alcohol during pregnancy, public health regions, child's sex, and the year of birth. Multiple comparison correction was applied using the False Discovery Rate approach. Maternal residential exposure to 1,2,4-trimethylbenzene, benzene, chlorine, ethylbenzene, and styrene was associated with LBW in offspring, with adjusted odds ratios ranging from 1.10 to 1.13. These five chemicals remained as significant risk factors after dividing the estimated exposure intensities into four categories. In addition, significant linear trends were found between LBW and maternal exposure to each of the five identified chemicals. Furthermore, 1,2,4-trimethylbenzene was identified as a risk factor to LBW for the first time.

Although statistically significant, the adjusted odds ratios reported in this study are of such low magnitude that their biological relevance is uncertain. Despite reporting significant linear trends for all five chemicals individually, the adjusted odds ratios are not quantifiable for ethylbenzene exposure alone, as the exposure was to air pollution generally rather than to specific chemicals. Exposures were estimated based on residential locations, excluded potential workplace exposures, and did not consider several potentially-important confounders for LBW, such as genetic factors, exposure to tobacco smoke, and exposure to other air pollutants, including those on the Criteria Air Pollutants (CAPs) list. As such, the results are not interpretable for use in the WoE evaluation.

Gong et al., 2018.
Gong, X., Lin, Y., Bell, M. L., & Zhan, F. B. (2018). Associations between maternal residential proximity to air emissions from industrial facilities and low birth weight in Texas, USA. Environ Int, 120, 181–198. https://doi.org/10.1016/j.envint.2018.07.045

Gong et al., (2018) is a forerunner to Gong et al., 2023 that investigated associations between maternal residential exposure to industrial air pollutants during pregnancy and low birth weight (LBW) in offspring using a case-control design that included 94,106 term LBW cases and 376,424 controls. The analysis covered 78 air pollutants common to both the Toxic Release Inventory and ground air quality monitoring databases in Texas during 1996 – 2008. The authors report an adjusted odds ratios for ethylbenzene of 1.05 (95% CI: 1.03 – 1.06). Although statistically significant, the biological significance of such a low odds ratio is questionable. Confounding exposures and inadequate control of other factors that could influence birth weight render the results uninterpretable for a WoE analysis of potential endocrine activity for ethylbenzene.

Supplemental Material C

Rationale for Excluding Studies

Harrath et al., 2022.

Harrath AH, Alrezaki A, Jalouli M, Aldawood N, Aldahmash W, Mansour L, & Alwasel S. (2022). Ethylbenzene exposure disrupts ovarian function in Wistar rats via altering folliculogenesis and steroidogenesis-related markers and activating autophagy and apoptosis. Ecotoxicol Environ Saf, 229, 113081. doi:10.1016/j.ecoenv.2021.113081.

In this repeat dose toxicity study, Harrath et al., (2022) exposed rats to EB for 30 minutes per day for 30 consecutive days to 2,000 ppm, 4,000 ppm, and 8,000 ppm EB. Ovary weight was slightly reduced at 2,000 ppm, but not at 4,000 or 8,000 ppm. Abnormal follicles were observed at 2,000 and 4,000 ppm but the effect was barely significant at 8,000 ppm. Circulating estradiol levels were increased at 4,000 but not at 2,000 or 8,000 ppm. Circulating testosterone was increased at 2,000 and 8,000 ppm, but not at 4,000 ppm and estrogen receptor numbers were also altered.

The lack of clear dose-response relationships for these various effects makes the results difficult to interpret, but this is the least of the problems with this study. All exposure levels produced significant apoptosis in the affected organs, which confounds the interpretation of an endocrine MoA as each endpoint was likely affected secondary to induction of apoptosis. The authors assert that apoptosis is the primary mechanism underlying various other effects observed in the study. Moreover, the reported levels of exposure strain credulity. The reported exposure concentrations are equivalent to 2.5X, 5X, and 10X the IDLH value* for human occupational exposures, and near within 1/4, 1/2, and 1X the explosive limit of the chemical. The lowest exposure level used in this study equals or exceeds the highest level used in other studies, and is tenfold above the KMD for EB (Burgoon et al., 2023). Even though the exposure durations were short (30 minutes), the degree of kinetic overload these would produce and the obvious confounding by apoptosis and other unknown mechanisms renders the effects reported by Harrath et al. (2022) unreliable and uninterpretable for the purposes of an endocrine WoE analysis.

Lei, T., Qian, H., Yang, J., Hu, Y.

Lei, T., Qian, H., Yang, J., & Hu, Y. (2023). The association analysis between exposure to volatile organic chemicals and obesity in the general USA population: A cross-sectional study from NHANES program. Chemosphere, 315, 137738. https://doi.org/10.1016/j.chemosphere.2023.137738

This study attempted to evaluate whether recent reports of an association between exposure to volatile organic chemical (VOC) pollutants, measured as urinary metabolites, and obesity are general, or associated specifically with abdominal obesity. Data from the 6 survey cycles (2005–2006, 2011–2018, 2017–2020) of the NHANES program were analyzed by 4 separate models in a cross-sectional study among a total of 17,524 participants (4965 obesity, 7317 abdominal obesity). Participants in the obesity or abdominal obesity groups showed higher VOCs in urine than were present in the control group. OR for obesity in the Q2 to Q4 of model 3 was 1.169 (Q2, $p < 0.05$), 1.306 (Q3, $p < 0.001$) and 1.217 (Q4, $p < 0.01$) respectively. The OR for abdominal obesity in the Q2 to Q4 of model 3 was 1.222 (Q2, $p < 0.01$), 1.448 (Q3, $p < 0.001$) and 1.208 (Q4, $p < 0.05$) respectively. A significantly positive association between urine levels of VOCs (Acrolein, Acrylamide, Acrylonitrile, 1,3-Butadiene, Crotonaldehyde, Cyanide, N,N-Dimethylformamide, Ethylbenzene, Styrene, Propylene oxide, Toluene and Xylene) and BMI and waist circumference was reported.

None of the analyses were specific to ethylbenzene, and the models employed were incapable of determining whether the direction of the associations, i.e., whether exposure begat obesity or obesity enhanced absorption of VOCs. The endpoints measured were unusable in the WoE evaluation due to confounding by multiple chemical exposures.

Supplemental Material C

Rationale for Excluding Studies

Nakhjirgan et al. 2019.

Nakhjirgan P, Kashani H, Naddafi K, Nabizadeh R, Amini H, & Yunesian M. (2019). Maternal exposure to air pollutants and birth weight in Tehran, Iran. J Environ Health Sci Eng, 17(2), 711-717. doi:10.1007/s40201-019-00386-7

Although the authors mention EB in the context of air pollutants in urban Tehran, Iran, the study evaluated potential associations between air pollutants broadly and health outcomes in pregnant women. Since exposures to EB were unspecified and uncertain and the results indicative only of potential associations with urban air generally, the results of the study are not informative regarding potential endocrine MoAs for EB.

Rouget et al., 2021.

Rouget F, Bihannic A, Cordier S, Multigner L, Meyer-Monath M, Mercier F, Pladys P, Garlantezec R. (2021). Petroleum and Chlorinated Solvents in Meconium and the Risk of Hypospadias: A Pilot Study. Front Pediatr, 9, 640064. doi:10.3389/fped.2021.640064

Rouget et al. (2021) conducted a pilot case-control study in the maternity unit of the University Hospital in Rennes, France to evaluate possible associations between the occurrence of hypospadias and fetal exposure to petroleum and chlorinated solvents measured in meconium. Since exposures to EB were unspecified and uncertain and the results indicative only of potential associations with petroleum and chlorinated solvents generally, the results of the study are not useful for an endocrine WoE evaluation.

Werder et al. 2019.

Werder EJ, Engel LS, Blair A, Kwok RK, McGrath JA, & Sandler DP. (2019). Blood BTEX levels and neurologic symptoms in Gulf states residents. Environ Res, 175, 100-107. doi:10.1016/j.envres.2019.05.004

Werder et al. (2019) evaluated potential associations between blood levels of BTEX chemicals (benzene, toluene, EB, and xylene) in Gulf coast residents of the United States who were transiently exposed to BTEX during the Deepwater Horizon oil spill and/or the response to it. Although the publication mentions endocrine disruptive effects of BTEX, the authors generated no data relevant to specific endocrine effects of EB.

Werder et al. 2020.

Werder EJ, Beier JI, Sandler DP, Falkner KC, Gripshover T, Wahlang B, . . . Cave MC. (2020). Blood BTEXS and heavy metal levels are associated with liver injury and systemic inflammation in Gulf states residents. Food Chem Toxicol, 139, 111242. doi:10.1016/j.fct.2020.111242.

Werder et al., 2020 conducted a clinical cross-sectional analysis to evaluate possible associations of biomarkers with serum livery injury and adipocytokine biomarkers in a sample of 214 men. No data relevant to specific endocrine effects of EB were reported. However, with respect to endocrine disruptive effects as speculated by the authors, their results suggest that rather than and endocrine mechanism, liver toxicity would be the likely mechanism that secondarily affects endocrine parameters.

Supplementary Table 1. Estrogen Agonist Hypothesis; Guideline Toxicity Studies					
Rank	Assay	Endpoint(s)	Expected Response	Response to Ethylbenzene	No Response to Ethylbenzene
1	FSTRA	Vitellogenin	↑ ♂		
	Uterotrophic	Uterus weight (blotted or wet)	↑		
2	Uterotrophic	Conversion to estrus	↑		
	ERTA	Reporter gene activation	↑		
	FSTRA	Behavioral (sexual, mating)	Δ ♂		
		Gonad histopathology	Δ ♂		
		Tubercle score	↓ ♂		
	Female Pubertal	Age and body weight at vaginal opening	↓		
		Age at first estrus	↓		
		Ovary histopathology	Δ		
		Ovary weight	↓		
	Male Pubertal	Testis histopathology (atrophy)	Δ		
		Testis weight	↓		
	Repeat Dose Toxicity	Epididymis histopathology	Δ		[1c,m,r] [3s,r] [9s,m,r] [10s,m,r,rb]
		Epididymis weight	↓	[9s,m]	[9s,r]
		Mammary histopathology	Δ		
		Ovary histopathology	Δ		[1c,m,r][3s,r] [9s,m,r] [10s,m,r,rb]
		Ovary weight	↓		
		Prostate weight	↓		
		Seminal vesicle weight	↓		
		Testis histopathology (atrophy)	↑		[1c,m,r] [9s,m,r]
		Testis weight	↓		[9s,m,r]
		Uterus histopathology	Δ		[1c,m,r] [3s,r] [9s,m,r] [10s,m,r,rb]
		Uterus weight	↑		
		Vaginal histopathology	Δ		[1c,m,r] [3s,r]
	Developmental Toxicity	Corpora lutea	↓		[5r][13r,rb]
		Post-implantation loss	↑	[11r] [13rb]	[4r] [5r] [6r] [11m,rb] [13r]

Supplementary Table 1. Estrogen Agonist Hypothesis; Guideline Toxicity Studies					
Rank	Assay	Endpoint(s)	Expected Response	Response to Ethylbenzene	No Response to Ethylbenzene
2	Developmental Toxicity	Pre-implantation loss	↑		[13r,rb]
		Time to vaginal patency	↓		
	Reproductive Toxicity	Anogenital distance	Δ ♂ , ♀		
		Corpora lutea	↓		
		Epididymis histopathology (atrophy)	Δ		
		Epididymis weight	↓		
		Estrous cyclicity	Δ	[2r,F_0]	[2r,F_1]
		Fertility	↓♂ , ♀		[2r,F_0,F_1]
		Gestational length	↓♀		[2r,F_0,F_1]
		Implantations	↓		[2r,F_0,F_1]
		Litter size	↓		[2r,F_0,F_1]
		Mammary histopathology	Δ ♀		
		Mating index	↓♂ , ♀		[2r,F_0,F_1]
		Ovarian follicle count in offspring	Δ		[2r,F_1]
		Ovary histopathology	Δ		
		Ovary weight in offspring	↓		
		Prostate histopathology (atrophy)	Δ		
		Prostate weight	↓		
		Seminal vesicle weight	↓		
		Sperm count	↓		[2r,F_0,F_1]
		Testis histopathology (atrophy)	Δ		
		Testis weight (absolute)	↓		
		Time to mating	↑♂ , ♀		[2r,F_0,F_1]
		Time to preputial separation	↑♂		
		Time to vaginal patency	↓	[2r,F_1]	[2r,F_2]
		Uterus histopathology	Δ		
		Uterus weight in offspring	↑		
		Vaginal histopathology	Δ		
3	ERBA	Displacement of Estradiol	↑		
	FSTRA	Behavior	Δ		
		Estradiol level	↓♀		
		Fecundity	↓		

Supplementary Table 1. Estrogen Agonist Hypothesis; Guideline Toxicity Studies					
Rank	Assay	Endpoint(s)	Expected Response	Response to Ethylbenzene	No Response to Ethylbenzene
3	FSTRA	Fertilization success	↓♀		
		Follicular atresia	↑		
		Gonad somatic index	↓♂, ↑♀		
		Testosterone level	↓♂		
	Female Pubertal	Estrous cyclicity	↑		
		Growth	↑		
	Male Pubertal	Epididymis histopathology	Δ		
		Growth	Δ		
		Ventral prostate weight	Δ		
	Steroidogenesis	Estradiol level	↑		
	Repeat Dose Toxicity	Gross pathology	Δ♂, ♀		[3s,r]

♂= males; ♀= females; ↑ = increase relative to controls; ↓ = decrease relative to controls; Δ = altered; ? = altered but not as expected. Numbers correspond to numbered studies in Appendix C; r = rat; m = mouse; rb = rabbit; s = subchronic; c = chronic; n = non-guideline; $F_0 = F_0$ generation; $F_1 = F_1$ generation; $F_2 = F_2$ generation.

Supplementary Table 2. Estrogen Antagonist Hypothesis; Guideline Toxicity Studies					
Rank	Assay	Endpoint(s)	Expected Response	Response to Ethylbenzene	No Response to Ethylbenzene
1	Uterotrophic	Uterus weight increase w E2	↓		
2	ERBA	Displacement of estradiol	↑		
	FSTRA	Gonad histopathology	Δ ♀		
		Vitellogenin	↓ ♀		
	Female Pubertal	Age and body weight at vaginal opening	↑		
		Age at first estrus	↑		
	Repeat Dose Toxicity	Epididymis histopathology	Δ		[1c,m,r] [3s,r] [9s,m,r] [10s,m,r,rb]
		Ovary histopathology	Δ		[1c,mr] [3s,r] [9s,m,r] [10s,m,r,rb]
		Prostate histopathology	Δ		[1c,m,r] [3s,r] [9s,m,r]
		Seminal vesicle histopathology	Δ		[1c,m,r] [3s,r] [9s,m,r]
		Testis histopathology (atrophy)	↑		[1c,m,r] [3s,r] [9s,m,r] [10s,m,r,rb]
		Testis weight	↓		[9s,m,r]
	Developmental Toxicity	Corpora lutea	↓		[5r] [13r,rb]
		Time to vaginal patency	↓		
	Reproductive Toxicity	Corpora lutea	↓		
		Epididymis histopathology (atrophy)	Δ		
		Estrous cyclicity	Δ	[2r,F_0]	[2r,F_1]
		Fertility	↓♂ , ♀		[2r,F_0,F_1]
		Litter size	↓		[2r,F_0,F_1]
		Ovary histopathology	Δ		
		Prostate histopathology (atrophy)	Δ		
		Seminal vesicle histopathology	Δ		

Supplementary Table 2. Estrogen Antagonist Hypothesis; Guideline Toxicity Studies					
Rank	Assay	Endpoint(s)	Expected Response	Response to Ethylbenzene	No Response to Ethylbenzene
2	Reproductive Toxicity	Sperm count	↓		[2r,F_0,F_1]
		Testis histopathology (atrophy)	Δ		
		Testis weight (absolute)	↓		
		Time to mating	↑♂, ♀		[2r,F_0,F_1]
		Time to vaginal patency	↑	[2r,F_1]	[2r,F_2]
3	Aromatase Inhibition	Aromatase inhibition	↓		
	FSTRA	Behavior	Δ♀		
		Estradiol level	↓♀		
		Fecundity	↓		
		Fertilization success	↓♀		
		Gonad somatic index	↓♀, ♂		
		Testosterone level	↓♂		
	Female Pubertal	Estrous cyclicity	Δ		
		Ovary histopathology (atrophy)	Δ		
		Ovary weight (with atrophy)	↓		
	Steroidogenesis	Estradiol level	↓		
	Repeat Dose Toxicity	Gross pathology	Δ♂, ♀		[3s,r]

♂= males; ♀= females; ↑ = increase relative to controls; ↓ = = decrease relative to controls; Δ = altered; ? = altered but not as expected. Numbers correspond to numbered studies in Appendix C; r = rat; m = mouse; rb = rabbit; s = subchronic; c = chronic; n = non-guideline; F_0 = F_0 generation; F_1 = F_1 generation; F_2 = F_2 generation.

Supplementary Table 3. Androgen Agonist Hypothesis; Guideline Toxicity Studies					
Rank	Assay	Endpoint(s)	Expected Response	Response to Ethylbenzene	No Response to Ethylbenzene
1	Hershberger	Concordance of 5 endpoints	↑		
	FSTRA	Secondary sexual characteristics: tubercles in females	↑		
2	ARBA	Displacement of testosterone	↑		
	FSTRA	Gonad histopathology	Δ		
		Vitellogenin	↓ ♀		
	Male Pubertal	Age & weight at preputial Separation: if accelerated	↓		
		Dorsolateral prostate weight	↑		
		Epididymis histopathology	Δ		
		Epididymis weight	↑		
		LABC weight	↑		
		Seminal vesicle + coagulating gland weight	↑		
		Testis histopathology (atrophy)	Δ		
		Testis weight	↑		
		Ventral prostate weight	↑		
	Hershberger	Concordance of 2 to 4 endpoints	↑		
	Repeat Dose Toxicity	Ovary histopathology	Δ		[1c,m,r] [3s,r] [9s,m,r] [10s,m,r,rb]
		Ovary weight	↑		
		Prostate weight	↓		
		Seminal vesicle weight	↓		
		Sperm count	↓		[9s,m,r]
		Testis histopathology (atrophy)	↑		[1c,m,r] [3s,r] [9s,m,r] [10s,m,r,rb]
		Testis weight	↓		[9s,m,r]
		Uterus weight	↑		
	Developmental Toxicity	Implantations	↓		[4r] [5r] [6r] [13r,rb]
		Litter size	↓	[13rb]	[4r] [5r] [6r] [13r]
		Masculinization of female offspring	↑		
		Sex ratio	Δ ♂ , ♀		[5r] [13r,rb]
		Time to balano-preputial separation	↓ ♂		
		Time to vaginal patency	↑		

Supplementary Table 3. Androgen Agonist Hypothesis; Guideline Toxicity Studies					
Rank	Assay	Endpoint(s)	Expected Response	Response to Ethylbenzene	No Response to Ethylbenzene
2	Reproductive Toxicity	Anogenital distance	↑♀		
		Estrous cyclicity	↓	[2r,F_0]	[2r,F_1]
		Fertility	↓♂, ♀		[2r,F_0,F_1]
		Implantations	↓		[2r,F_0,F_1]
		Litter size	↓		[2r,F_0,F_1]
		Masculinization of female offspring	↑♀		
		Mating index	↓♂, ♀		[2r,F_0,F_1]
		Nipple retention	↑♂		
		Ovarian follicle count	↓		
		Ovary histopathology	Δ		
		Ovary weight in offspring	↑		
		Prostate weight	↓		[2r,F_0,F_1]
		Seminal vesicle weight	↓		
		Sex ratio	Δ♂, ♀		[2r,F_1,F_2]
		Sperm count	↓		[2r,F_0,F_1]
		Testis histopathology (atrophy)	Δ		
		Testis weight	↓		
		Time to balano-preputial separation	↓♂	[2r,F_1]	[2r,F_2]
		Time to mating	↑♀		[2r,F_0,F_1]
		Time to vaginal patency	↑	[2r,F_1]	[2r,F_2]
3	Aromatase	Aromatase activity	↑		
	FSTRA	Behavior	Δ		
		Estradiol level	Δ		
		Fecundity	Δ		
		Fertilization success	Δ		
		Gonad somatic index	Δ		
		Testosterone level	Δ		
	Female Pubertal	Adrenals weight	↓		
		Age & weight at vaginal opening	↑		
		Growth	↑		
		Ovary histopathology	Δ		
		Ovary weight	↓		
		Uterus histopathology	Δ		
		Uterus weight	↑		
	Male Pubertal	Growth	↑		
		Testosterone level	↓		
	Steroidogenesis	Testosterone level	Δ		
	Hershberger	Concordance of 1 endpoint	↑		
	Repeat Dose Toxicity	Gross pathology	Δ♂, ♀		[3s,r]

♂= males; ♀= females; ↑ = increase relative to controls; ↓ = decrease relative to controls; Δ = altered; ? = altered but not as expected. Numbers correspond to numbered studies in Appendix C; r = rat; m = mouse; rb = rabbit; s = subchronic; c = chronic; n = non-guideline; F_0 = F_0 generation; F_1 = F_1 generation; F_2 = F_2 generation.

Supplementary Table 4. Androgen Antagonist Hypothesis; Guideline Toxicity Studies					
Rank	Assay	Endpoint(s)	Expected Response	Response to Ethylbenzene	No Response to Ethylbenzene
1	Hershberger	Concordance of 5 endpoints	↓		
2	ARBA	Displacement of testosterone	↑		
	FSTRA	Gonad histopathology	Δ ♂		
		Secondary sexual characteristics	↓ ♂		
		Vitellogenin	↑ ♀		
	Male Pubertal	Age & weight at preputial separation: if delayed	↑		
		Dorsolateral prostate weight	↓		
		Epididymis histopathology	Δ		
		Epididymis weight	↓		
		LABC weights	↓		
		Seminal vesicle + coagulating gland weight	↓		
		Testis histopathology (atrophy)	Δ		
		Testis weight	↓		
		Ventral prostate weight	↓		
	Hershberger	Concordance of 2 to 4 endpoints	↓		
	Repeat Dose Toxicity	Epididymal weight	↓	[9s,m]	[9s,r]
		Epididymis histopathology	Δ		[1c,m,r] [3s,r] [9s,m,r] [10s,m,r,rb]
		Ovary histopathology	Δ		[1c,m,r] [3s,r] [9s,m,r] [10s,m,r,rb]
		Prostate histopathology	Δ		[1c,m,r] [3s,r] [9s,m,r]
		Prostate weight	↓		
		Seminal vesicle histopathology	Δ		[1c,m,r] [3s,r] [9s,m,r]
		Seminal vesicle weight	↓		
		Testis histopathology (atrophy)	↑		[1c,m,r] [3s,r] [9s,m,r] [10s,m,r,rb]
		Testis weight	Δ		[9s,m,r]
		Uterus histopathology	Δ		[1c,m,r] [3s,r] [9s,m,r] [10s,m,r,rb]

Supplementary Table 4. Androgen Antagonist Hypothesis; Guideline Toxicity Studies

Rank	Assay	Endpoint(s)	Expected Response	Response to Ethylbenzene	No Response to Ethylbenzene
2	Developmental Toxicity	Time to balano-preputial separation	↑♂		
	Reproductive Toxicity	Anogenital distance	↓♂		
		Epididymis weight	↓		
		Epididymis histopathology	Δ		
		Estrous cyclicity	↓	[2r,F_0]	[2r,F_1]
		Fertility	↓♂,♀		[2r,F_0,F_1]
		Gross pathology	Δ♂,♀		[2r,F_0,F_1]
		Litter size	↓		[2r,F_0,F_1]
		Nipple retention	↑♂		
		Ovary histopathology	Δ		
		Prostate histopathology	Δ		
		Prostate weight	↓		[2r,F_0,F_1]
		Seminal vesicle histopathology	Δ		
		Seminal vesicle weight	↓		
		Sperm count	↓		[2r,F_0,F_1]
		Sperm motility	↓		[2r,F_0,F_1]
		Testis histopathology (atrophy)	Δ		
		Testis weight	↓		
		Time to balano-preputial separation	↑♂	[2r,F_1]	[2r,F_2]
		Time to mating	↑♀		[2r,F_0,F_1]
		Uterus histopathology	Δ		
3	FSTRA	Behavior	Δ		
		Estradiol level	Δ		
		Fecundity	↓		
		Fertilization success	↓		
		Gonad-somatic index	↓		
		Testosterone level	↑♂		
	Male Pubertal	Testosterone level	↑		
	Steroidogenesis	Testosterone level	Δ		
	Hershberger	Concordance of 1 endpoint	↓		
	Repeat Dose Toxicity	Gross pathology	Δ♂,♀		[3s,r]

♂= males; ♀= females; ↑ = increase relative to controls; ↓ = decrease relative to controls; Δ = altered; ? = altered but not as expected. Numbers correspond to numbered studies in Appendix C; r = rat; m = mouse; rb = rabbit; s = subchronic; c = chronic; n = non-guideline; F_0 = F_0 generation; F_1 = F_1 generation; F_2 = F_2 generation.

Supplementary Table 5. Thyroid Inhibition Hypothesis; Guideline Toxicity Studies

Rank	Assay	Endpoint(s)	Expected Response	Response to Ethylbenzene	No Response to Ethylbenzene
1	Male Pubertal	Thyroid histopathology	Δ		
		Thyroid weight	↑		
	Female Pubertal	Thyroid (colloid area & follicular cell height)	↑		
		Thyroid weight	↑		
	AMA	Asynchronous development	↑		
		Thyroid histopathology	Δ		
2	Female Pubertal	Age & weight at vaginal opening	↑		
		T4 level	↑		
		TSH level	↑		
	Male Pubertal	Liver weight	Δ		
		T4 level	↑		
		TSH level	↑		
	Repeat Dose Toxicity	Thyroid follicular cell histopathology	Δ ♂ , ♀	[1c,m]	[1c,r][3s,r] [9s,m,r] [10s,m,r,rb]
		Thyroid hormones	Δ ♂ , ♀		
		Thyroid weight	↑ ♂ , ♀		
	Developmental Toxicity	Fetal survival	↓ ♂ , ♀	[11rb] [13rb]	[4r] [5r] [6r] [11r] [13r]
		Fetal weight	↓ ♂ , ♀	[4r] [5r] [6r] [11r,rb]	[11m] [13r,rb]
		Thyroid follicular cell histopathology	Δ ♂ , ♀		
		Thyroid hormones	Δ ♂ , ♀		
		Thyroid weight	↑ ♂ , ♀		
	Reproductive Toxicity	Fetal weight	↓ ♂ , ♀		
		Pup growth	↓ ♂ , ♀		[2r,F_1,F_2]
		Pup survival	↓ ♂ , ♀		[2r,F_1,F_2]
		Pup weight	↓ ♂ , ♀		
		Thyroid follicular cell histopathology	Δ ♂ , ♀		
		Thyroid hormones	Δ ♂ , ♀		
		Thyroid weight	↑ ♂ , ♀	[2r,F_0♂]	[2r,F_0♀,F_1]
3	Male Pubertal	Age & weight at preputial separation	↑		
		Growth	↓		
		Pituitary weight	↓		

Supplementary Table 5. Thyroid Inhibition Hypothesis; Guideline Toxicity Studies					
Rank	Assay	Endpoint(s)	Expected Response	Response to Ethylbenzene	No Response to Ethylbenzene
3	Female Pubertal	Estrous cyclicity (diestrus)	Δ		
		Ovary histopathology	Δ		
		Ovary weight	↓		
	AMA	Delayed development	↑		
		Hind limb length	↓		
		Snout-vent length	Δ		
		Wet weight	↑		
	Repeat Dose Toxicity	Liver weight	↑♂, ♀	[3s,r] [6r] [7s,r] [8s,m] [9s,m,r] [10s,m,r]	[10s,rb]
	Reproductive Toxicity	Liver weight	↑♂, ♀	[2r,F_0,F_1]	
	Developmental Neurotoxicity	Auditory Startle	↓♂, ♀		[12,F_2]
		Behavioral Ontogeny	↓♂, ♀		
		Brain Morphometry	Δ♂, ♀		[12F_2]
		Learning and Memory	↓♂, ♀		[12F_2]
		Liver weight	↑♂, ♀		
		Motor Activity	Δ♂, ♀		[12F_2]
		Myelination	Δ♂, ♀		
		Pup growth	↓♂, ♀		
		Pup survival	↓♂, ♀		

♂= males; ♀= females; ↑ = increase relative to controls; ↓ = decrease relative to controls; Δ = altered; ? = altered but not as expected. Numbers correspond to numbered studies in Appendix C; r = rat; m = mouse; rb = rabbit; s = subchronic; c = chronic; n = non-guideline; F_0 = F_0 generation; F_1 = F_1 generation; F_2 = F_2 generation.

Supplementary Table 6. Interaction with Steroidogenesis Enzymes Hypothesis; Guideline Toxicity Studies					
Rank	Assay	Endpoint(s)	Expected Response	Response to Ethylbenzene	No Response to Ethylbenzene
1	Female Pubertal	Uterus weight	↓		
2	Female Pubertal	Ovary weight	↓		
	FSTRA	Gonad histopathology: males	Δ		
		Vitellogenin	↓♀		
	Steroidogenesis	Estradiol level	↓		
		Testosterone level	↓		
	Repeat Dose Toxicity	Ovary histopathology	Δ		[1c,m,r] [3s,r] [9s,m,r] [10s,m,r,rb]
		Ovary weight	↓		
		Testis histopathology (atrophy)	↑		[1c,m,r] [3s,r] [9s,m,r] [10s,m,r,rb]
		Uterus histopathology	Δ		[1,c,m,r] [3s,r] [9s,m,r] [10s,m,r,rb]
		Uterus weight	↓		
	Developmental Toxicity	Sex ratio	Δ♂, ♀		[5r] [13r,rb]
	Reproductive Toxicity	Estrous cyclicity	Δ	[2r,F_0]	[2r,F_1]
		Fertility	↓♂, ♀		[2r,F_0,F_1]
		Live births	↓♀		[2rF_0,F_1]
		Mating index	↓♂		[2r,F_0,F_1]
		Ovary histopathology	Δ		
		Parturition	↓		
		Post-implantation loss	↑		
		Resorptions	↑		
		Sex ratio	Δ♂, ♀		[2r,F_0,F_1]
		Sexual behavior	Δ♂		
		Sperm count	↓		[2r,F_0,F_1]
		Testicular histopathology (atrophy)	Δ		
		Uterus histopathology	Δ		
		Uterus weight	↓		

Supplementary Table 6. Interaction with Steroidogenesis Enzymes Hypothesis; Guideline Toxicity Studies					
Rank	Assay	Endpoint(s)	Expected Response	Response to Ethylbenzene	No Response to Ethylbenzene
3	Aromatase	Aromatase activity	↓		
	Female Pubertal	Age & weight at vaginal opening	↑		
		Age at first estrus	↑		
	Male Pubertal	Testosterone level	Δ		
	FSTRA	Behavior	Δ		
		Estradiol level	Δ		
		Fecundity	Δ		
		Fertilization success	Δ		
		Gonad-somatic index	Δ		
		Testosterone level	Δ		
	Repeat Dose Toxicity	Gross pathology	Δ ♂ , ♀		[3s,r]

♂= males; ♀= females; ↑ = increase relative to controls; ↓ = decrease relative to controls; Δ = altered; ? = altered but not as expected. Numbers correspond to numbered studies in Appendix C; r = rat; m = mouse; rb = rabbit; s = subchronic; c = chronic; n = non-guideline; F_0 = F_0 generation; F_1 = F_1 generation; F_2 = F_2 generation.

Supplementary Table 7. Summary of Endpoints from all Tables

MoA	Fraction of Rank 1 Endpoints Tested	# of Rank 1 Endpoints Responding to Ethylbenzene	# of Rank 1 Endpoints Showing No Response to Ethylbenzene	Fraction of Rank 2 Endpoints Tested	# of Rank 2 Endpoints Responding to Ethylbenzene	# of Rank 2 Endpoints Showing No Response to Ethylbenzene	Fraction of Rank 3 Endpoints Tested	# of Rank 3 Endpoints Responding to Ethylbenzene	# of Rank 3 Endpoints Showing No Response to Ethylbenzene
Estrogen Agonist - Table 1	0 (2)	0	0	20 (53)	4	20	1 (15)	0	1
Estrogen Antagonist - Table 2	0 (1)	0	0	13 (26)	2	13	1 (12)	0	1
Androgen Agonist - Table 3	0 (2)	0	0	18 (47)	4	18	1 (19)	0	1
Androgen Antagonist - Table 4	0 (1)	0	0	17 (45)	3	17	1 (10)	0	1
Thyroid Inhibition - Table 5	0 (6)	0	0	6 (21)	4	6	6 (21)	2	5
Steroidogenesis - Table 6	0 (1)	0	0	10 (25)	1	10	1 (11)	0	1
Hershberger endpoints counted as one concordance response									
